# Isolation of cationic and neutral (allenylidene)(carbene) and bis(allenylidene)gold complexes†
†Electronic supplementary information (ESI) available. CCDC 1418631–1418633. For ESI and crystallographic data in CIF or other electronic format see DOI: 10.1039/c5sc03654b



**DOI:** 10.1039/c5sc03654b

**Published:** 2015-11-16

**Authors:** Liqun Jin, Mohand Melaimi, Arseni Kostenko, Miriam Karni, Yitzhak Apeloig, Curtis E. Moore, Arnold L. Rheingold, Guy Bertrand

**Affiliations:** a UCSD-CNRS Joint Research Chemistry Laboratory (UMI 3555) , Department of Chemistry and Biochemistry , University of California San Diego , La Jolla , CA 92093-0358 , USA . Email: guybertrand@ucsd.edu; b Schulich Faculty of Chemistry , Technion - Israel Institute of Technology , Haifa 32000 , Israel

## Abstract

The one-electron reduction of a cationic (allenylidene)[cyclic(alkyl) (amino)carbene]gold(i) complex leads to the corresponding neutral, paramagnetic, formally gold(0) complex. DFT calculations reveal that the spin density of this highly robust coinage metal complex is mainly located on the allenylidene fragment, with only 1.8 and 3.1% on the gold center and the CAAC ligand, respectively. In addition, the first homoleptic bis(allenylidene)gold(i) complex has been prepared and fully characterized.

## Abstract 2

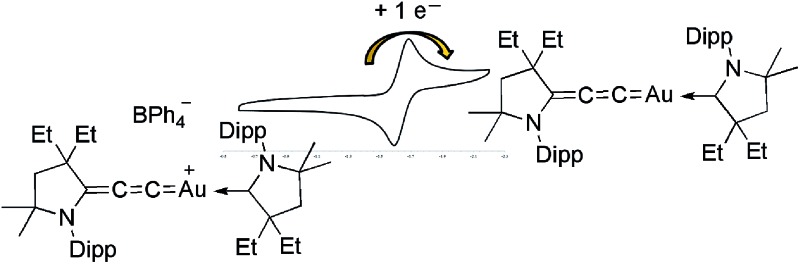
The one-electron reduction of a cationic (allenylidene)[cyclic(alkyl)(amino)carbene]gold(i) complex leads to the corresponding neutral, paramagnetic, formally gold(0) complex.

## Introduction

**Chart 1:**
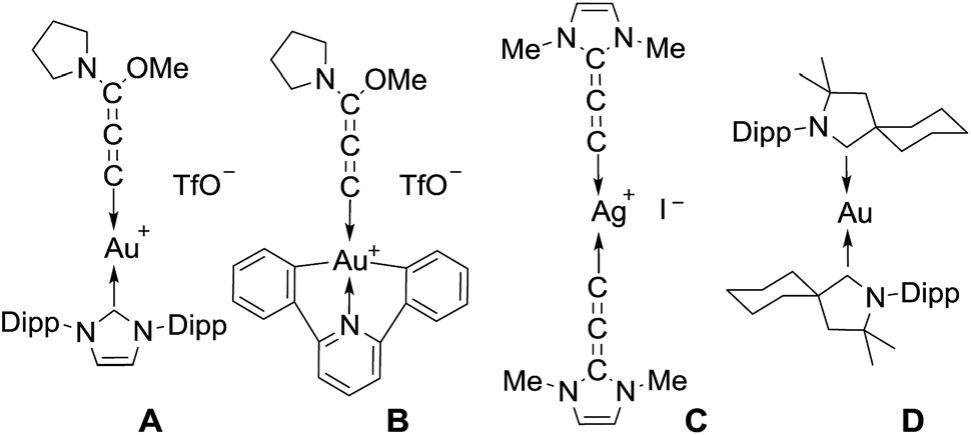


Allenylidene transition metal complexes were first isolated in 1976 by Fischer and Berke.^1^ The π interactions between the metal ion and the rigid, linear allenylidene framework make these complexes of interest for molecular wires and electronic materials; they have also been postulated as key intermediates in catalysis.^2^ The chemistry of metal allenylidene complexes is largely dominated by Cr, W, Mn, Ru, Os, Ir, Pd and Pt,^3^ while studies dealing with coinage metals are limited. Hashmi *et al.* ^4^ and Che *et al.* ^5^ prepared the Au(i) and Au(iii) complexes **A** and **B**, respectively, featuring an (alkoxy) (amino)allenylidene, while we isolated a silver(i) complex **C**, featuring the (diamino)allenylidene^6^ (Chart 1). Recently, we and others reported that cyclic (alkyl) (amino)carbene (CAAC)^7^ ligands allow for the isolation of complexes featuring a metal in the formal zero oxidation state,^8,9^ including the neutral complex **D**, the first example of a compound with a gold(0) center.^10^ The unusual stability of **D** is due to the π-accepting properties of CAAC ligands.^11^ Theoretical calculations have clearly established that allenylidenes are likewise σ-donor and π-acceptor ligands with a dominant contribution of the latter component to the bonding, and thus are attractive ligands for stabilizing gold(0) complexes.^11,12^

Herein, we report the preparation of several stable allenylidene gold complexes, including the first neutral (allenylidene)(carbene)gold and cationic bis(allenylidene)gold complexes.

## Results and discussion

**Scheme 1:**
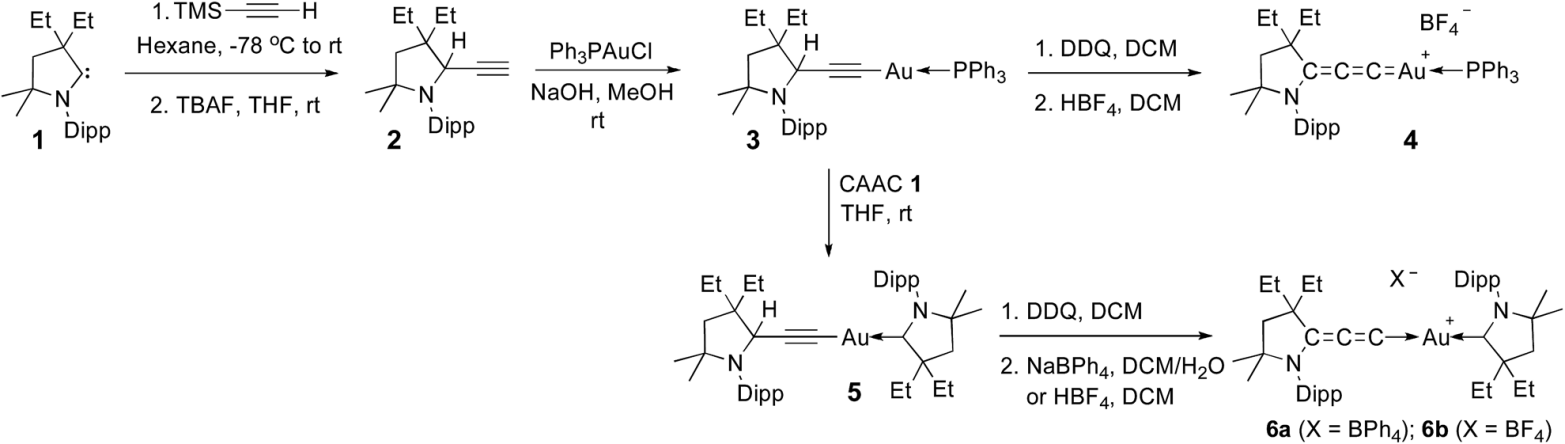
Synthesis of allenylidene gold(i) complexes **4** and **6a,b**. Dipp = 2,6-diisopropylphenyl, TBAF = tetrabutylammonium fluoride.

**Fig. 1:**
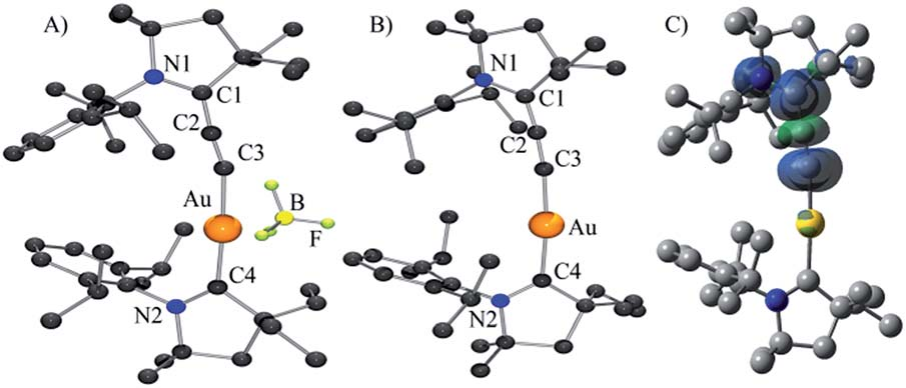
Solid-state structure of complexes **6b** (A), **7** (B) (H-atoms omitted for clarity), and calculated spin-density for complex **7** (C).

Although aryl- or alkyl-substituted allenylidenes have stronger π-accepting properties, their complexes are usually much less stable than those featuring a π-donating heteroatom.^4^ Therefore we chose to target allenylidenes featuring one amino substituent. As shown in Scheme 1, alkyne **2** was prepared by formal insertion of a CAAC^13,14^ into the CH-bond of trimethylsilylacetylene, followed by treatment with tetrabutylammonium fluoride. In the presence of sodium hydroxide, alkyne **2** reacts with Ph_3_PAuCl in methanol, affording the gold–acetylide complex **3** in 84% yield. Lastly, a hydride abstraction^15^ with 2,3-dichloro-5,6-dicyano-1,4-benzoquinone (DDQ), followed by anion exchange with HBF_4_, led to the cationic allenylidene gold complex **4** as a white solid, but in only 37% yield. Moreover, this complex appeared to decompose rapidly in solution (*vide infra*). Thus, we decided to replace the triphenylphosphine ligand with a CAAC. Addition at room temperature of CAAC **1** to the acetylide complex **3** readily afforded complex **5**, which was obtained in 78% yield. Using the same procedure as for **3**, the desired cationic gold allenylidene complexes **6a,b** with BPh_4_ or BF_4_ counter-anion were isolated as light yellow, air and water stable solids in good yields. The ^13^C NMR signal of the C3 and C2 nuclei of the allenylidene fragment are at 185.9 and 97.6 ppm, downfield and highfield shifted by 62.5 and 8.4 ppm, respectively, compared to those of **5**. A similar trend has been observed for other late transition metal allenylidenes *versus* acetylide complexes.^16^ Single crystals suitable for X-ray diffraction were obtained by slow diffusion of diethyl ether into a dichloromethane solution of **6b** (Fig. 1A).^17^

The cyclic voltammetry of (allenylidene) (CAAC)Au(i) **6b** was carried out in THF using 0.1 M *n*Bu_4_NPF_6_ as supporting electrolyte. A reversible one electron reduction at *E*_1/2_ = –1.73 V *versus* Fc^+^/Fc was observed. This reduction potential is shifted towards a more positive value compared to that observed for bis(CAAC)Au(i) complex (*E*_1/2_ = –2.24 V),^10^ demonstrating the superior π-accepting properties of allenylidenes compared to CAACs. Encouraged by these results, the chemical reduction of complex **6b** was carried out with one equivalent of KC_8_ in THF at room temperature. A dark yellow solution was obtained, which was NMR silent and EPR active. The room temperature EPR spectrum of **7** in benzene (see ESI†) shows a broad signal centered at *g* = 1.983. The highly shifted *g* factor, compared with free electron *g* = 2.002, is typical to heavy-element-containing radicals due to strong spin–orbit coupling.

The DFT calculated *g* factor (at ZORA/B3LYP/TZVP//M05-2X/SDD/def2-SVP level of theory) of 1.985 (for full details, see the “Computational methods” section below) is in excellent agreement with experiment. Yellow single crystals of the neutral complex **7** were grown from a concentrated THF solution at –20 °C (Fig. 1B). Detailed comparison of the geometric parameters of **6b** and **7** is not possible because two superimposed molecules are present in the unit cell of **6b**. At the M05-2X/SDD/def2-SVP level of theory the C2–C3 and C1–C2 bonds slightly elongates and shortens, respectively, upon reduction [C2–C3: **6b** 1.229, **7** 1.243; C1–C2: **6b** 1.399, **7** 1.380]. The calculations also predict a significant elongation of the C1–N1 bond upon reduction [**6b**: 1.304; **7**: 1.386]. These data suggest that the electron is added to the allenylidene ligand. This hypothesis is supported by Mulliken spin density analysis of **7** computed at the M05-2X/SDD/def2-SVP//M05-2X/SDD/def2-SVP level of theory. Indeed, 93.9% of the spin density resides on the CCC fragment, while only 3.8 and 1.8% are located on the CAAC ligand and the Au atom, respectively (Fig. 1C). This is also reflected in the charges. Upon reduction the gold atom and the CAAC moiety gain only 0.02 el. and 0.10 el., respectively, while the calculated charge in the allenylidene moiety increases by 0.88 el. Consequently, **7** has only a very weak gold(0) character, even weaker than complex **D**, in which 17% of the spin density is located at gold;^10^ in other words it can be viewed as a gold(i) complex featuring a paramagnetic anionic ligand.

**Scheme 2:**
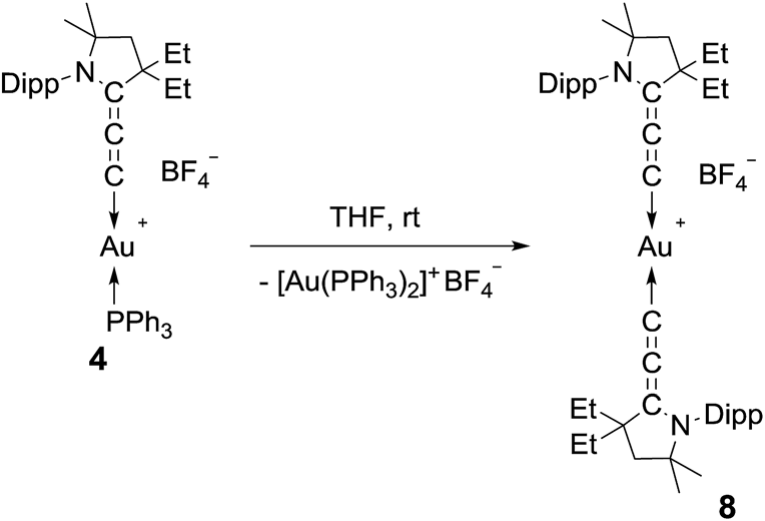
Dismutation of **4** into the homoleptic bis(allenylidene)gold(i) complex **8**.

We then turned our attention to the decomposition process of the (allenylidene) (triphenylphosphine)gold(i) complex **4**. To our delight, this complex undergoes a dismutation reaction, affording bis(triphenylphosphine)gold(i) along with the bis(allenylidene)gold(i) complex **8**, which was isolated in 11% yield as a moisture and air stable solid (Scheme 2). So far, only bis(allenylidene) complexes of Ag,^6^ Pd,^6,18^ Pt,^18^ and Ru^16^ have been isolated. ^1^H and ^13^C NMR spectroscopy of **8** clearly shows a symmetrical structure. Only three different signals ascribed to the cumulenic carbon nuclei were displayed in the ^13^C NMR spectrum (185.8, 177.7 and 95.9 ppm). Because of the poor yield and rather difficult separation procedure, we looked for a more efficient synthetic route leading to bis(allenylidene) gold complex **8**.

**Scheme 3:**
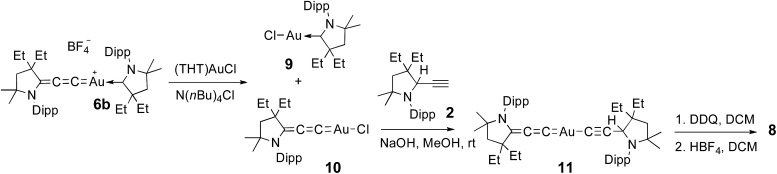
Synthesis of bis(allenylidene)gold(i) complex **8**. THT = tetrahydrothiophene.

**Fig. 2:**
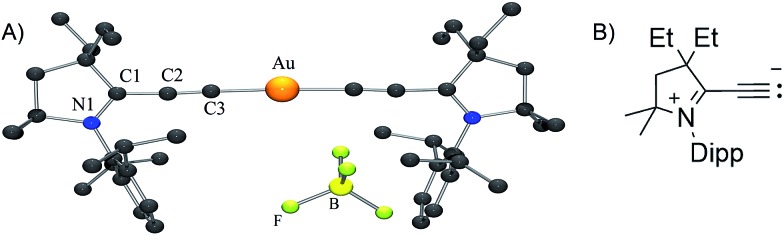
Solid-state structure of complex **8** (H-atoms omitted for clarity).

No dismutation was observed with the (allenylidene) (CAAC)gold(i) complex **6a**. However, we found that it reacts with one equivalent of (THT)AuCl in the presence of a chloride source to afford (CAAC)AuCl **9** and (allenylidene)AuCl **10** which were isolated in 74 and 82% yields, respectively (Scheme 3). Both compounds were characterized by ^1^H and ^13^C NMR spectroscopy, as well as by high-resolution mass spectrometry. By subjecting complex **10** to the same synthetic sequence as for **3**, the corresponding gold–acetylide complex **11** was obtained in 76% yield. Subsequent hydride abstraction by DDQ followed by anion exchange led to the bis(allenylidene) gold complex **8** in 76% isolated yield. Single crystals were obtained by slow evaporation of a saturated THF solution, and subjected to an X-ray diffraction study (Fig. 2A). The C1–C2 [1.427(8) Å] and C2–C3 [1.176(8) Å] bond distances are close to standard values for carbon–carbon single and triple bonds, respectively, and therefore the ligands in complex **8** are best described by the resonance form depicted in Fig. 2B.

The cyclic voltammogram of a THF solution of bis(allenylidene)gold(i) complex **8** showed two well defined one-electron reversible reductions at –1.695 and –2.045 V *versus* Fc^+^/Fc. The first reduction potential is shifted to an even more positive value than that of the (allenylidene)(CAAC)gold(i) complex **6b**, further supporting that the allenylidene behaves as a stronger π-accepting ligand than CAACs. Chemical reduction of **8** with CoCp_2_* resulted in a dark green solution, which was NMR silent and EPR active, but all attempts to grow single crystals led to decomposition.

## Conclusions

Although a variety of cumulene transition metal complexes are known, compounds **6a,b**, **7**, **8**, **10** and **11** are rare examples in the coinage metal series.^4–6,19^ Because of their π-accepting properties, the allenylidenes, derived from CAACs, proved to be excellent ligands for electron-rich paramagnetic complexes, featuring a formal gold(0) center. The physical properties^5^ of these novel robust complexes are under active investigation.

## Experimental section

### Synthesis and characterization

#### Preparation of cationic (allenylidene)(CAAC)Au complex **6a**

Dichloromethane (30 mL) was added to a mixture of **5** (594 mg, 0.7 mmol) and 2,3-dichloro-5,6-dicyano-1,4-benzoquinone (159 mg, 0.7 mmol). The reaction mixture was stirred for 30 minutes at room temperature. A solution of sodium tetraphenylborate (342 mg, 1.0 mmol) in water (30 mL) was added to the reaction mixture. After vigorous stirring for 30 minutes, the organic layer was separated and dried over MgSO_4_. After filtration, the solvent was removed under minutes, the organic layer was separated and dried over MgSO_4_. After filtration, the solvent was removed under vacuum. The solid residue was washed with diethyl ether (3 × 10 mL), affording complex **6** as a pale yellow solid. Yield 83% (678 mg). mp 202.1 °C (dec.). ^13^C{1H} NMR (CDCl_3_, 125 MHz): *δ* = 250.8 (C_carbene_), 185.9 and 177.0 (*C*Au and *C*CCAu), 164.4 (*C*_i-BPh4_), 145.1 (C_q_), 145.0 (C_q_), 136.4 (C_*o*-BPh4_), 133.8 (C_q_), 131.1 (CH_Ar_), 129.9 (CH_Ar_), 129.9 (CH_Ar_), 125.6 (C_*m*-BPh4_), 125.3 (CH_Ar_), 124.9 (CH_Ar_), 121.6 (C_*p*-BPh4_), 97.6 (*C*CAu), 83.8 (C_q_), 81.5 (C_q_), 62.8 (C_q_), 58.0 (C_q_), 42.1 (CH_2_), 41.9 (CH_2_), 31.5, 31.3, 29.8, 29.3, 29.1, 28.8, 26.9, 26.5, 23.0, 22.9, 9.66, 8.97; HRMS (ESI-TOFMS): *m*/*z* calculated for [C_46_H_70_AuN_2_]^+^ 847.5199, found 847.5197. Complex **6** (BF_4_) was synthesized following the same procedure but using tetrafluoroboric acid instead of NaBPh_4_. Single crystals suitable for X-ray diffraction analysis were obtained by diffusion of diethyl ether in a dichloromethane solution of **6** (BF_4_).

#### Preparation of neutral (allenylidene)(CAAC)Au complex **7**

THF (10 mL) was added at room temperature to a mixture of KC_8_ (22 mg, 0.16 mmol) and **6b** (150 mg, 0.16 mmol). The dark mixture was stirred at room temperature for 2 hours. The solvent was removed under vacuum, and the residue was extracted with benzene. Evaporation of the filtrate under vacuum led to **7** as a dark green solid. Yield 53% (72 mg). Single crystals suitable for X-ray diffraction analysis were obtained from a concentrated tetrahydrofuran solution at –20 °C.

#### Preparation of cationic bis(allenylidene)Au complex **8**

Dichloromethane (20 mL) was added to a mixture of **11** (1.0 g, 1.1 mmol) and 2,3-dichloro-5,6-dicyano-1,4-benzoquinone (275 mg, 1.2 mmol). The reaction mixture was stirred for 30 minutes at room temperature. Tetrafluoroboric acid diethyl ether complex was added dropwise until the dark red color faded to pale yellow. After filtration, the volatiles were removed under vacuum and the residue was washed with benzene (10 mL). The resulting solid was further washed with THF (3 × 10 mL) to afford **8** as an off-white solid. Yield 76% (830 mg). mp 185.1 °C (dec.). ^13^C NMR (CDCl_3_, 125 MHz): *δ* = 185.8 and 177.7 (*C*Au and *C*CAu), 145.3 (*C*_q_), 131.2 (*C*H_Ar_), 129.7 (*C*_q_), 125.4 (*C*H_Ar_), 95.9 (C*C*CAu), 77.6 (*C*_q_), 58.6 (*C*_q_), 41.6 (*C*H_2_), 31.7 (*C*H_2_), 29.9, 28.8, 26.7, 22.9, 9.0. HRMS (ESI-TOFMS): *m*/*z* calculated for [C_48_H_70_AuN_2_]^+^ 871.5199, found 871.5200. Single crystals suitable for X-ray diffraction analysis were obtained by slow evaporation of a saturated THF solution of **8** at room temperature.

### Computational methods

Geometry optimizations of **6** and **7** were carried out using the M05-2X method^20^ (global hybrid functional with 52% HF exchange) with Ahlrichs' def2-SVP basis set.^21^ The Au relativistic effect was accounted for by the Stuttgart-Dresden ECP.^22^ This method is referred to as M05-2X/SDD/def2-SVP. NBO analysis was carried out using the NBO 6.0 software.^23^ The *g* factor of **7** at its optimized geometry was calculated using the B3LYP (ref. 24) (hybrid functional with 20% HF exchange) functional with the TZVP basis set^25^ and the zero-order regular approximation (ZORA)^26^ for the relativistic effect in Au. The method is referred to as ZORA/B3LYP/TZVP. The calculations were performed using the Gaussian 09 (ref. 27) and ORCA 3.0.1 (ref. 28) programs. Full details of the calculations are given in the ESI.†
