# Supplementary material for: Isolation of cationic and neutral (allenylidene)(carbene) and bis(allenylidene)gold complexes
Source: Chem Sci. 2015 Nov 16;7(1):150–4. doi: 10.1039/c5sc03654b (PMC5580305; doi:10.1039/c5sc03654b)
Supplement: Supplementary file 1 [file SC-007-C5SC03654B-s001.pdf]

Electronic Supplementary Material (ESI) for Chemical Science.
This journal is © The Royal Society of Chemistry 2015

Supporting Information

Isolation of cationic and neutral (allenylidene)(carbene) and bis(allenylidene)gold Complexes

Liqun Jin,[a] Mohand Melaimi,[a] Arseni Kostenko,[b] Miriam Karni,[b] Yitzhak Apeloig,[b] Curtis E. Moore,[a] Arnold. L. Rheingold,[a] and Guy Bertrand[a]

[a] UCSD-CNRS Joint Research Chemistry Laboratory (UMI 3555), Department of Chemistry and Biochemistry, University of California - San Diego, La Jolla, California 92093-0358, United States

[b] Technion - Israel Institute of Technology, Schulich Faculty of Chemistry, Haifa 32000 (Israel)

E-mail: guybertrand@ucsd.edu

CONTENTS

1. **General remarks** **2**

2. **Experimental details** **3**

3. **Electrochemistry details** **18**

4. **EPR Spectroscopy** **18**

5. **Crystallographic details** **19**

6. **Computational details** **34**

1. General remarks

^{1}H and ^{13}C NMR spectra were recorded on Jeol ECA 500 and Varian Inova 500 spectrometers. ^{31}P NMR spectra were recorded on Bruker Avance 300. NMR multiplicities are abbreviated as follows: *s* = singlet, *d* = doublet, *t* = triplet, *q* = quartet, *sept* = septet, *m* = multiplet, *br* = broad signal. Chemical shifts are given in ppm and are referenced to $SiMe_4$ (^{1}H, ^{13}C) and H_3PO_4 (^{31}P). All spectra were obtained in the solvent indicated at 25 °C. Coupling constants *J* are given in Hz. Mass spectra were performed at the UC San Diego Mass Spectrometry Laboratory. Melting points were measured with an electrothermal MEL-TEMP apparatus. Single crystal X-ray diffraction data were collected on a Bruker Apex II-CCD detector using Mo-Kα radiation (λ = 0.71073 Å). Crystals were selected under oil, mounted on nylon loops, then immediately placed in a cold stream of N_2. Structures were solved and refined using Olex2 and SHELXTL. $CAAC_{Et}$ (**1**) and $AuCl(PPh_3)$, were prepared following literature procedures while all other starting materials were purchased from commercial sources.

2. Experimental details

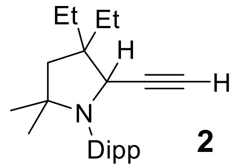

2

Trimethylsilylacetylene (490 mg, 5.0 mmol) was added to a solution of **1** (1.3 g, 4.1 mmol) in hexane (20 mL) at -78 °C. After stirring overnight at room temperature, the volatiles were removed under vacuum. The resulting solid (1.5 g) was dissolved in THF, and added to solution of TBAF·$3H_2O$ (1.4 g, 4.4 mmol) in THF (20 mL). The mixture was stirred for 3 hours at room temperature. The volatiles were removed under vacuum and the residue was dissolved in diethyl ether (20 mL) and water (30 mL). The organic layer was separated and the aqueous layer was extracted with diethyl ether (2 x 15 mL). The combined organic layer was dried with $MgSO_4$. After filtration, the solvent was evaporated under vacuum. The residue was purified by flash chromatography on silica gel with hexanes as eluant to yield the alkyne **2** as a white solid. Yield 93% (1.3 g). m.p. 89 °C.

^{1}H NMR ($CDCl_3$, 500 MHz): δ = 7.22 (t, J = 7.8 Hz, 1 H, *p*-H), 7.13 (d, J = 7.8 Hz, 1 H, *m*-H), 7.11 (d, J = 7.8 Hz, 1 H, *m*-H), 4.51 (d, J = 2.3 Hz, 1H, C*H*CCH), 3.82 (sept, J = 6.9 Hz, 1 H, C*H*Me$_2$), 3.26 (sept, J = 6.9 Hz, 1 H, C*H*Me$_2$), 2.03 (d, J = 2.3 Hz, CC*H*), 2.02-1.98 (m, 2 H, C*H*$_2$), 1.85-1.74 (m, 1 H, C*H*$_2$CH$_3$), 1.69-1.55 (m, 2 H, C*H*$_2$CH$_3$), 1.55-1.44 (m, 1 H, C*H*$_2$CH$_3$), 1.28-1.19 (m, 9 H, CH(C*H*$_3$)$_2$), 1.17-1.13 (m, 6 H, CH(C*H*$_3$)$_2$, CH$_2$C*H*$_3$), 1.06 (s, 3 H, C*H*$_3$), 0.96 (t, J = 7.5 Hz, 6 H, CH$_2$C*H*$_3$); ^{13}C{^{1}H} NMR ($CDCl_3$, 125 MHz): δ = 153.2 (C_q), 151.5 (C_q), 137.2 (C_q), 126.8 (CH$_{Ar}$), 124.0 (CH$_{Ar}$), 123.9 (CH$_{Ar}$), 84.6 (C_q), 73.9 (CCH), 64.4 (CH), 61.7, 50.5 (CH$_2$), 46.4, 31.3 (CH$_2$), 30.4 (CH$_2$), 29.7, 28.2, 27.7, 26.6, 26.5, 25.8, 24.0, 23.7, 9.5, 8.8. HRMS (ESI-TOFMS): m/z calculated for $[C_{24}H_{38}N]^+$ 340.2999, found 340.2995;

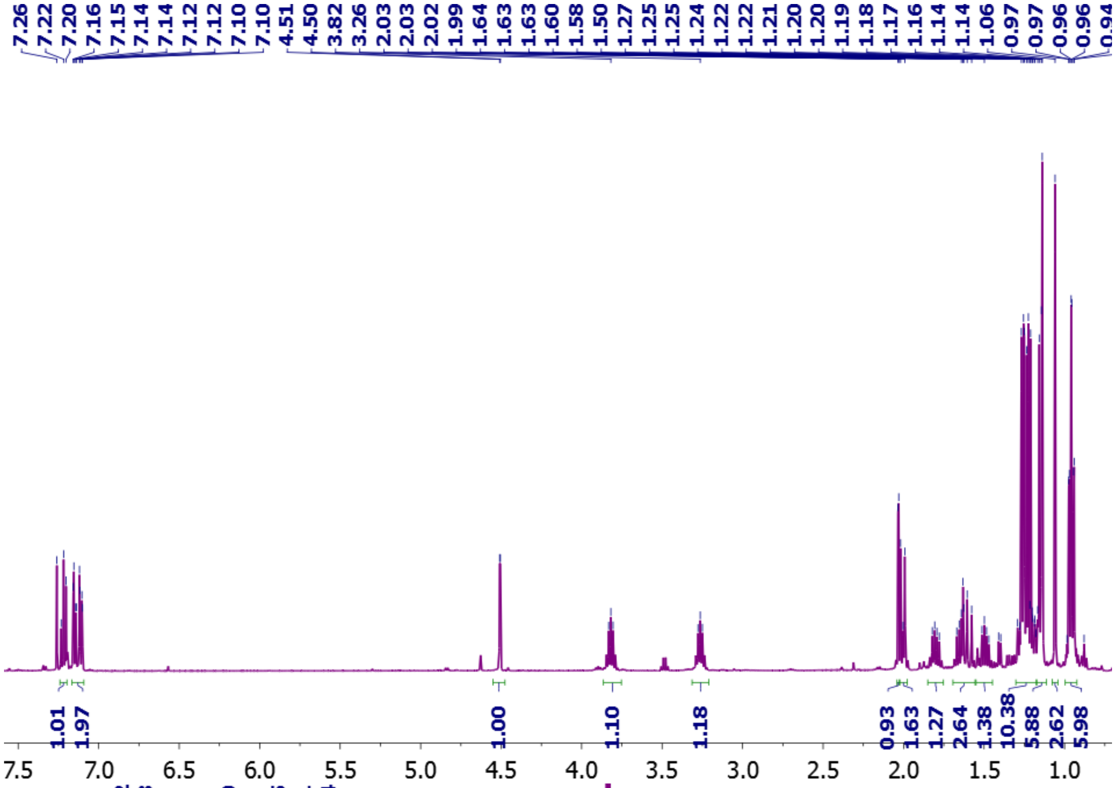
7.26
7.22
7.20
7.16
7.15
7.14
7.14
7.12
7.10
7.10
4.51
4.50
3.82
3.26
2.03
2.03
2.02
1.99
1.64
1.63
1.63
1.60
1.58
1.50
1.27
1.25
1.25
1.24
1.22
1.22
1.21
1.20
1.20
1.19
1.18
1.17
1.16
1.14
1.14
1.06
0.97
0.97
0.96
0.96
0.94
1.01
1.97
1.00
1.10
1.18
0.93
1.63
1.27
2.64
1.38
10.38
5.88
2.62
5.98
7.5 7.0 6.5 6.0 5.5 5.0 4.5 4.0 3.5 3.0 2.5 2.0 1.5 1.0

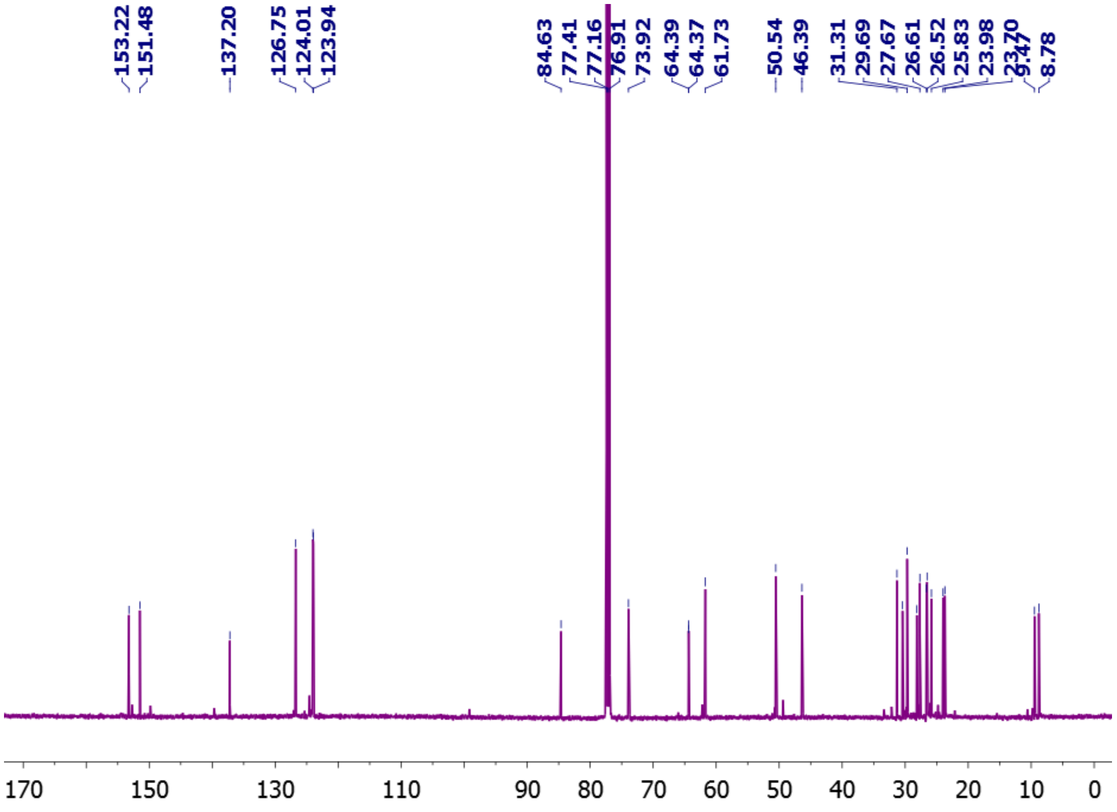
153.22
151.48
137.20
126.75
124.01
123.94
84.63
77.41
77.16
76.91
73.92
64.39
64.37
61.73
50.54
46.39
31.31
29.69
27.67
26.61
26.52
25.83
23.98
23.70
9.47
8.78
170 150 130 110 90 80 70 60 50 40 30 20 10 0

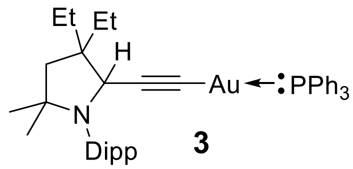

3

$AuCl(PPh_3)$ (400 mg, 0.8 mmol) was added to a solution of **2** (305 mg, 0.9 mmol) and NaOH (323 mg, 8 mmol) in methanol (10 mL). The mixture was stirred for 2 hours at room temperature while a white solid precipitated. After filtration, the white solid was extracted with dichloromethane (10 mL). After removal of volatiles under vacuum, complex **3** was obtained as a white solid. Yield: 84% (540 mg). m.p. 189.4 ^{o}C (dec.)

^{31}P NMR ($CDCl_3$, 121 MHz): δ = 28.2; ^{1}H NMR ($CDCl_3$, 500 MHz): δ = 7.56-7.30 (m, 15 H, CH_{PPh3}), 7.15 (t, J = 7.8 Hz, 2 H, *m*-H), 7.09 (d, J = 7.8 Hz, 1 H, *p*-H), 4.66 (s, 1H, C*H*CC), 3.99 (sept, J = 6.9 Hz, 1 H, C*H*Me$_2$), 3.32 (sept, J = 6.9 Hz, 1 H, C*H*Me$_2$), 2.20-2.07 (m, 1 H, C*H*$_2$CH$_3$), 2.06-1.93 (m, 2 H, C*H*$_2$CH$_3$, C*H*$_2$), 1.851-1.58 (m, 2 H, C*H*$_2$CH$_3$, C*H*$_2$), 1.57-1.47 (m, 1 H, C*H*$_2$CH$_3$), 1.41 (d, J = 6.9 Hz, 3 H, CHC*H*$_3$), 1.30 (d, J = 6.9 Hz, 3 H, CHC*H*$_3$), 1.26 (d, J = 6.9 Hz, 3 H, CHC*H*$_3$), 1.22 (d, J = 6.9 Hz, 3 H, CHC*H*$_3$), 1.18 (s, 3 H, C*H*$_3$), 1.05-0.92 (m, 9 H, C*H*$_3$, CH$_2$C*H*$_3$); ^{13}C{^{1}H} NMR ($CDCl_3$, 125 MHz): δ = 153.6 (C_q), 151.7 (C_q), 138.6 (C_q), 134.4 (d, J_{CP} = 11.0 Hz, $C_{m\text{-PPh3}}$,), 131.4 (br, $C_{p\text{-PPh3}}$), 130.3 (d, J_{CP} = 54.2 Hz, $C_{ipso\text{-PPh3}}$), 129.0 (d, J_{CP} = 13.7 Hz, $C_{o\text{-PPh3}}$), 126.1 (CH$_{Ar}$), 126.0 (d, J_{CP} = 143.6 Hz, CAu), 123.78 (CH$_{Ar}$), 123.5 (CH$_{Ar}$), 104.0 (d, J_{CP} = 26.5 Hz, CCAu), 65.9 (CH), 61.5, 49.3 (CH$_2$), 46.1, 31.2 (CH$_2$), 31.0 (CH$_2$), 29.7, 28.8, 27.8, 27.6, 27.4, 26.4, 24.5, 23.5, 9.8, 9.1; HRMS (ESI-TOFMS): m/z calculated for $[C_{42}H_{52}AuNP]^+$ 798.3497, found 798.3498;

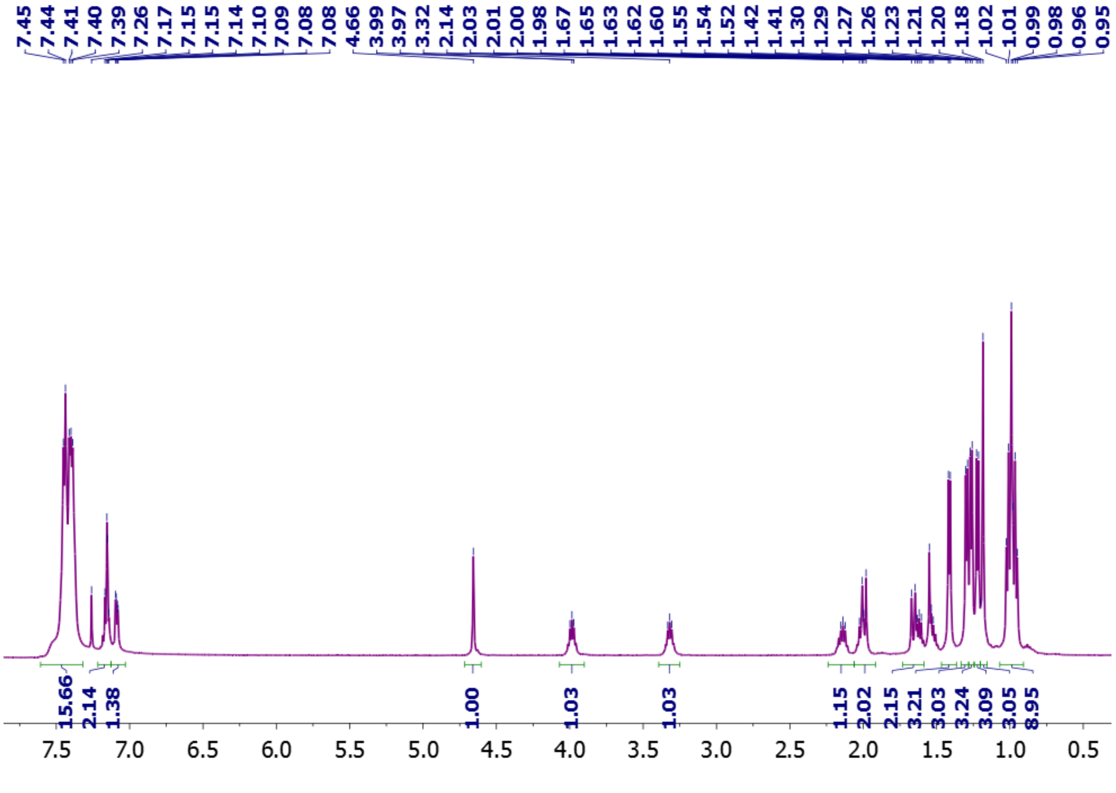

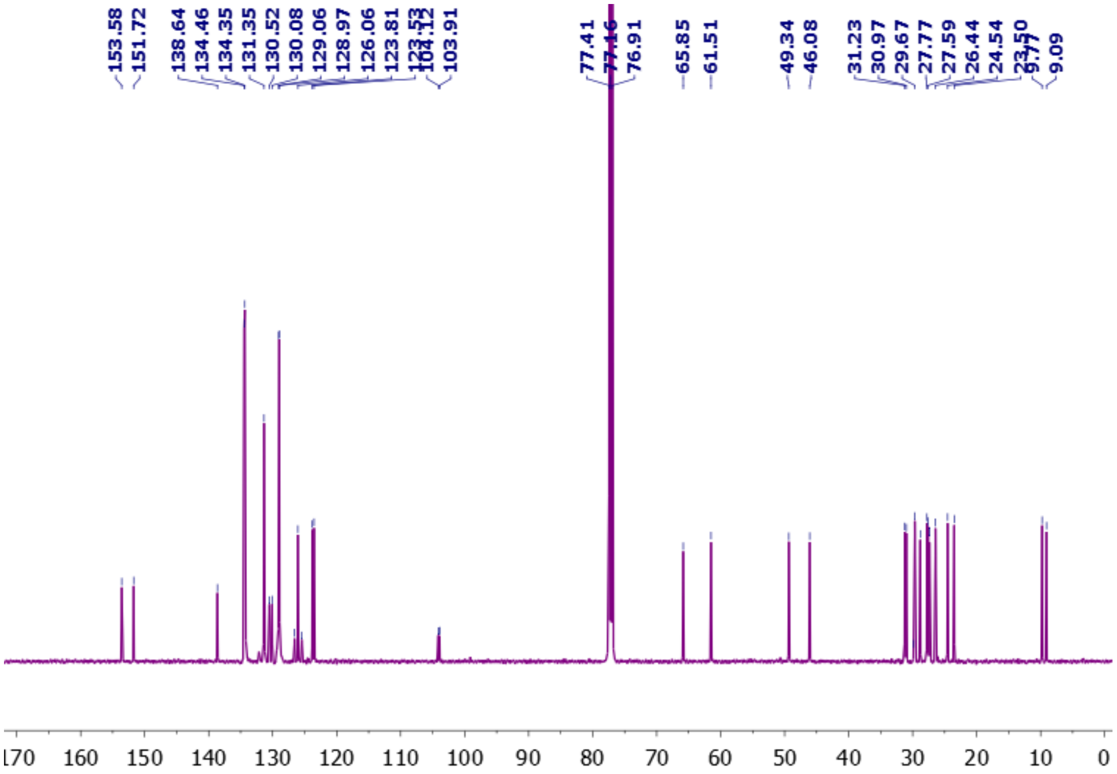

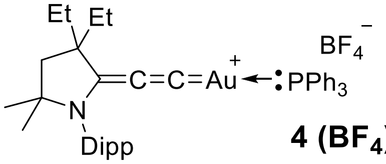

4 (BF_4)

Dichloromethane (20 mL) was added to a mixture of **3** (1.5 g, 1.9 mmol) and 2,3-dichloro-5,6-dicyano-1,4-benzoquinone (470 mg, 2.0 mmol). The reaction mixture was stirred for 30 minutes at room temperature. Tetrafluoroboric acid diethyl ether complex was added drop by drop until the dark red color faded to pale yellow. After filtration, the volatiles were removed under vacuum and the residue was washed with benzene (10 mL). The resulting solid was further washed with THF (3 x 10 mL) to afford **6** as an off-white solid. Yield 37 % (620 mg). m.p. 198.3 ^{o}C (dec.).

^{31}P NMR ($CDCl_3$, 121 MHz): δ = 45.1; ^{1}H NMR ($CDCl_3$, 500 MHz): δ = 7.60-7.52 (m, 4 H, CH_{PPh3}, *p*-Dipp), 7.51-7.45 (m, 6 H, CH_{PPh3}), 7.44-7.34 (m, 8 H, CH_{PPh3}, *m*-Dipp), 2.67 (sept, J = 6.9 Hz, 2 H, C*H*Me$_2$), 2.45 (s, 2 H, CH_2), 2.08-1.89 (m, 4 H, CH_2CH$_3$), 1.51 (s, 6 H, CH_3), 1.34 (d, J = 6.9 Hz, 6 H, CHCH_3), 1.26 (d, J = 6.9 Hz, 6 H, CHCH_3), 1.14 (t, J = 7.5 Hz, 6 H, CH$_2$CH_3); ^{13}C{^{1}H} NMR ($CDCl_3$, 125 MHz): δ = 183.8 (d, J_{CP} = 138.8 Hz, *C*Au), 178.4 (d, J_{CP} = 2.7 Hz, *C*CCAu), 145.2 (C_q), 134.2 (d, J_{CP} = 13.8 Hz, CH_{PPh3},), 132.3 (d, J_{CP} = 2.4 Hz, CH_{PPh3}), 131.5 (CH_{Ar}), 129.6 (C_q), 129.6 (d, J_{CP} = 11.8 Hz, CH_{PPh3}), 128.3 (d, J_{CP} = 60.4 Hz, C_{PPh3}), 125.6 (CH_{Ar}), 94.2 (d, J_{CP} = 25.4 Hz, C*C*CAu), 78.9 (C_q) 58.9 (C_q), 41.4 (CH_2), 31.7 (CH_2), 31.0 (CH_2), 30.0, 28.8, 26.8, 23.0, 9.2. HRMS (ESI-TOFMS): m/z calculated for $[C_{42}H_{52}AuNP]^+$ 796.3341,

found 796.3345.

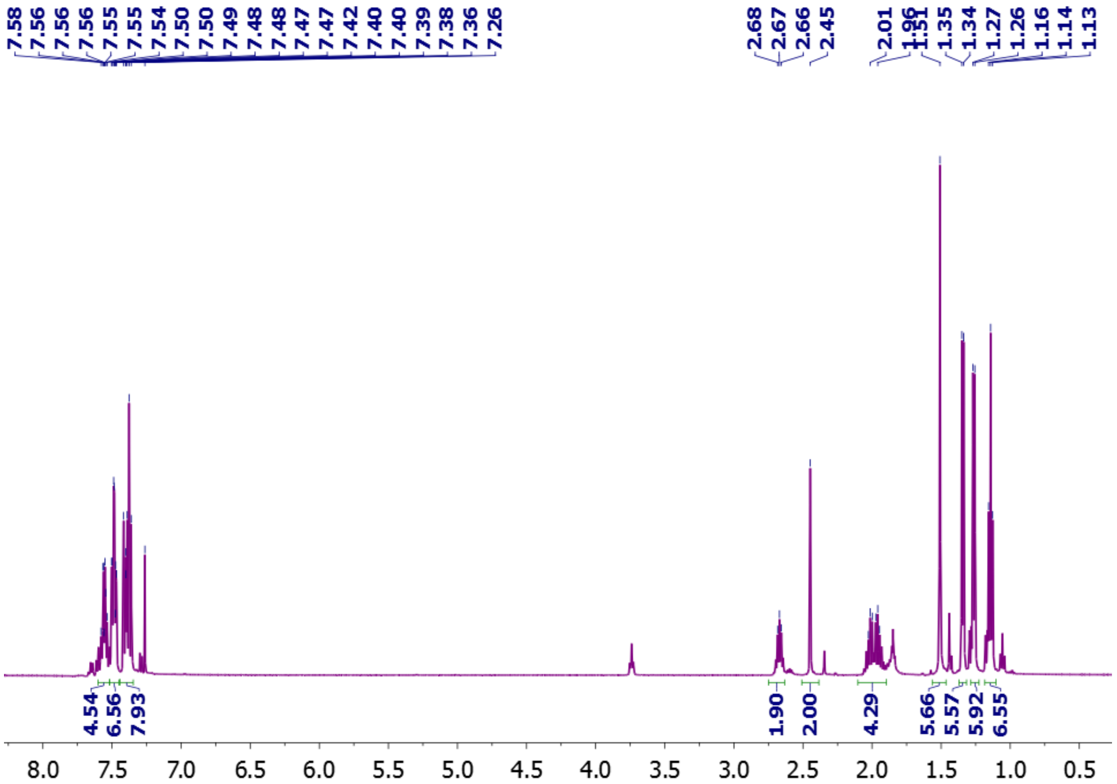

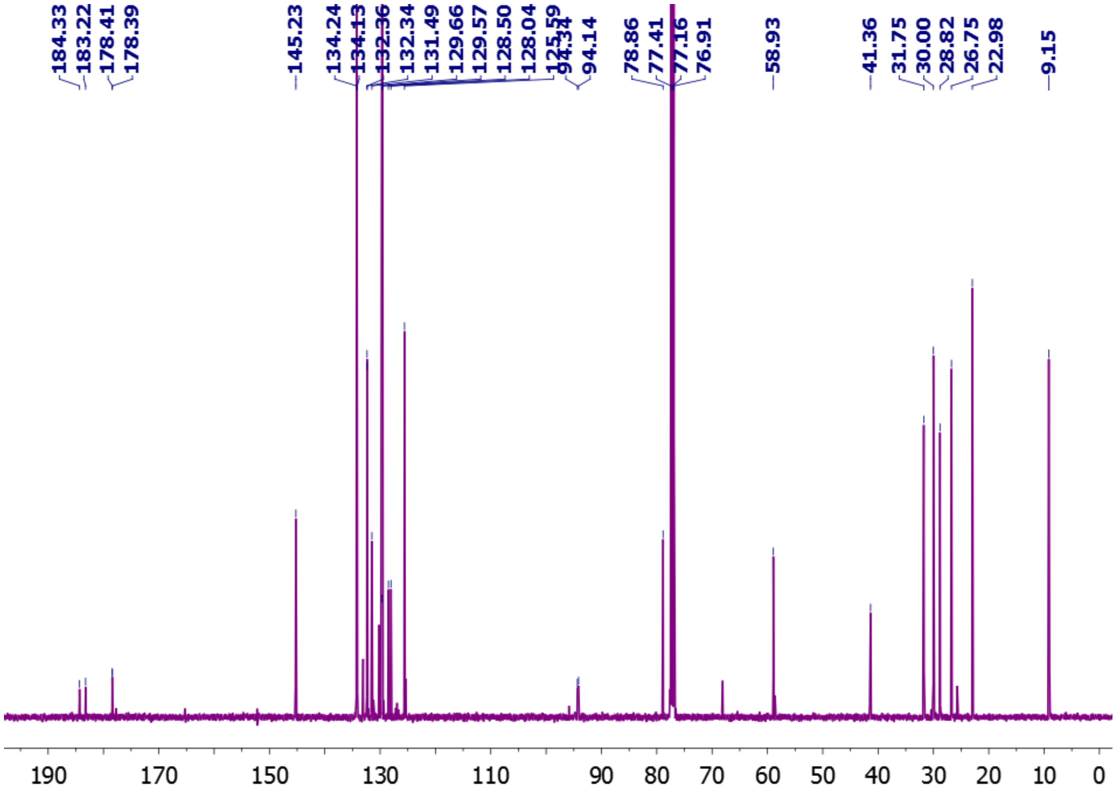

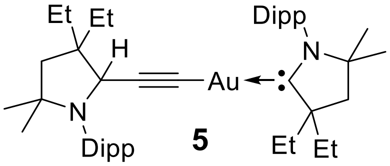

5

THF (10 mL) was added to a mixture of **3** (400 mg, 0.50 mmol) and **1** (170 mg, 0.54 mmol) at room temperature and was stirred for 30 minutes at room temperature. The solvent was removed under vacuum. The residue was washed with hexanes (3 x 5 mL). Complex **5** was isolated as a white solid. Yield 78% (330 mg). m.p. 199.5 ^{o}C (dec.)

^{1}H NMR ($CDCl_3$, 500 MHz): δ = 7.39 (t, J = 7.8 Hz, 1 H, p-H), 7.20-7.13 (m, 2 H, m-H), 7.09 (t, J = 7.8 Hz, 1 H, m-H), 6.97 (d, J = 7.8 Hz, 2 H, p-H), 4.57 (s, 1H, CHCC), 3.76 (sept, J = 6.9 Hz, 1 H, CHMe$_2$), 3.24 (sept, J = 6.9 Hz, 1 H, CHMe$_2$), 2.79-2.54 (m, 2 H, CHMe$_2$), 1.92 (s, 2 H, CH_2), 1.87-1.65 (m, 6 H, CH_2CH$_3$, CH_2), 1.54-1.36 (m, 4 H, CH_2CH$_3$, CH_2), 1.28 (br, 6 H, CH_3), 1.26-1.19 (m, 9 H, CHCH_3, CH_3), 1.20-1.08 (m, 12 H, CHCH_3), 1.02 (s, 3 H, CH_3), 0.97-0.81 (m, 18 H, CH_3, CH$_2$CH_3); ^{13}C{^{1}H} NMR ($CDCl_3$, 125 MHz): δ = 255.6 ($C_{carbene}$), 153.6 (C_q), 151.9 (C_q), 145.3 (C_q), 145.2 (C_q), 138.3 (C_q), 129.3 (CH$_{Ar}$), 128.9 (CH$_{Ar}$), 125.7 (CH$_{Ar}$), 124.8 (CH$_{Ar}$), 123.4 (CCAu), 123.2 (CH$_{Ar}$), 106.0 (CCAu), 80.2 (C_q), 65.5 (CH), 63.2, 60.6, 49.7(CH$_2$), 46.2, 41.8 (CH$_2$), 31.5 (CH$_2$), 30.7(CH$_2$), 30.5, 29.6 (CH$_2$), 29.3, 28.4, 27.6, 27.3, 27.2, 27.0, 26.4, 23.9, 23.6, 23.0, 22.9, 9.84, 9.67, 9.10. HRMS (ESI-TOFMS): m/z calculated for $[C_{46}H_{72}AuN_2]^+$ 849.5356, found 849.5354.

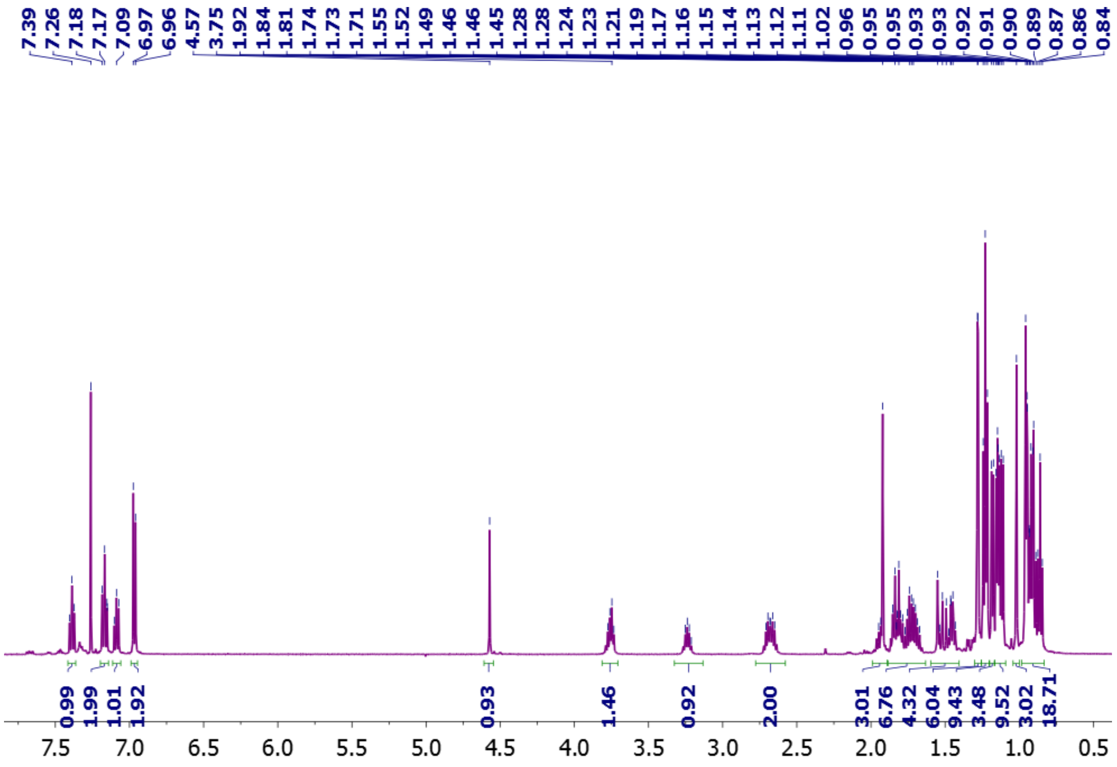

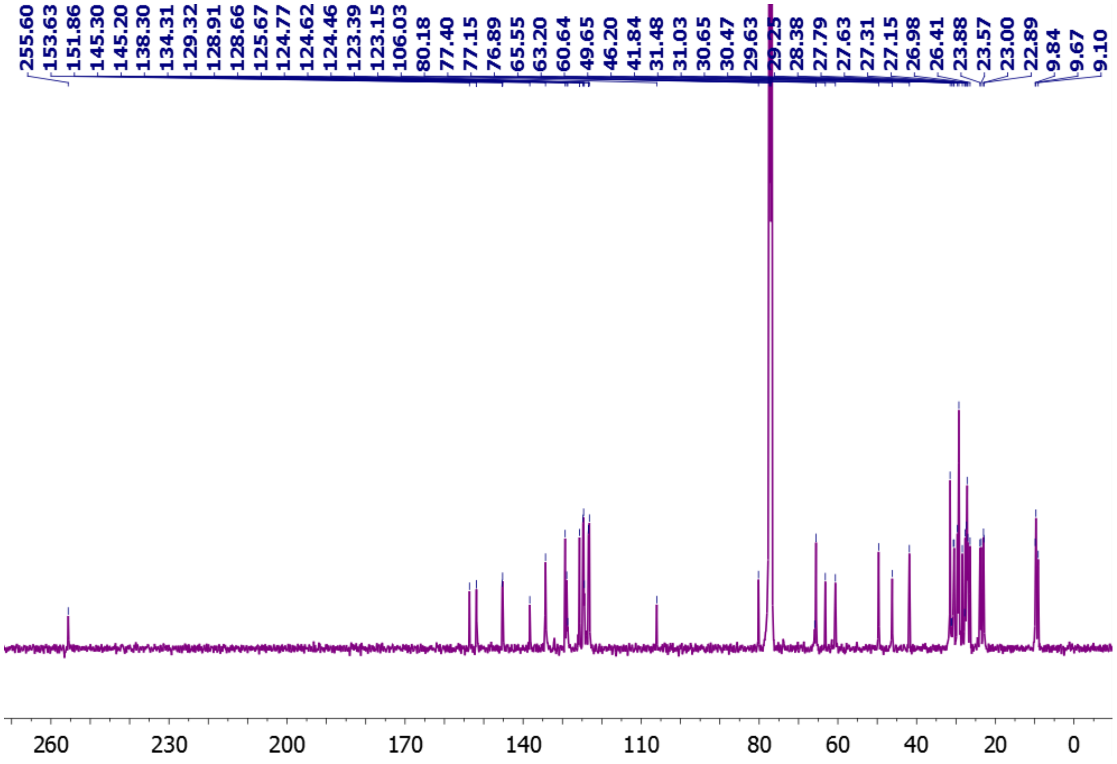

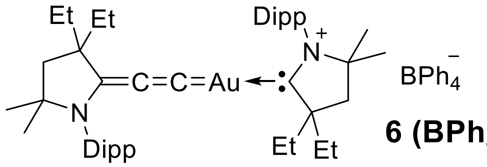

6 (BPh$_4$)

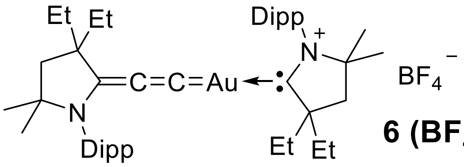

6 (BF$_4$)

Dichloromethane (30 mL) was added to a mixture of **5** (594 mg, 0.7 mmol) and 2,3-dichloro-5,6-dicyano-1,4-benzoquinone (159 mg, 0.7 mmol). The reaction mixture was stirred for 30 minutes at room temperature. A solution of sodium tetraphenylborate (342 mg, 1.0 mmol) in water (30 mL) was added to the crude reaction mixture and was stirred vigorously for 5 minutes. The organic layer was separated and dried with MgSO4. After filtration, the solvent was removed under vacuum. Finally, the solid residue was washed with diethyl ether (3 x 10 mL). Complex **6a** was isolated as a pale yellow solid. Yield 83 % (678 mg). m.p. 202.1 ^{o}C (dec.). Complex **6b** was synthesized following the same procedure using tetrafluoroboric acid instead of sodium tetraphenylborate. Single crystals suitable for X-ray diffraction analysis were obtained by diffusion of diethyl ether in a dichloromethane solution of **6b**.

^{1}H NMR ($CDCl_3$, 500 MHz): δ = 7.48-7.37 (m, 10 H, H_{BPh4} and *p*-H), 7.22 (d, *J* = 7.8 Hz, 2 H, *m*-H), 7.17 (d, *J* = 7.8 Hz, 2 H, *m*-H), 7.06 (t, *J* = 7.5 Hz, 8 H, H_{BPh4}), 6.91 (t, *J* = 7.5 Hz, 4 H, *p*-H_{BPh4}), 2.63 (sept, *J* = 6.9 Hz, 2 H, C*H*Me_2), 2.44 (sept, *J* = 6.9 Hz, 2 H, C*H*Me_2), 2.11 (s, 2 H, CH_2), 1.99 (s, 2 H, CH_2), 1.87-1.61 (m, 8 H, C$H_2$$CH_3$), 1.32 (s, 6 H, C$H_3$), 1.28 (s, 6 H, C$H_3$), 1.27-1.23 (m, 12 H, CHCH_3), 1.06 (d, 6 H, *J* = 6.9 Hz, CHCH_3), 1.02-0.90 (m, 18 H, CHCH_3, CH_2CH_3); ^{13}C{^{1}H} NMR ($CDCl_3$, 125 MHz): δ = 250.8 ($C_{carbene}$), 185.9 and 177.0 (*C*Au and *C*CCAu), 164.4 ($C_{i\text{-}BPh4}$), 145.1 (C_q), 145.0 (C_q), 136.4 ($C_{o\text{-}BPh4}$), 133.8 (C_q), 131.1 (CH_{Ar}), 129.9

(CH$_{Ar}$), 129.9 (CH$_{Ar}$), 125.6 ($C_{m\text{-BPh4}}$), 125.3 (CH$_{Ar}$), 124.9 (CH$_{Ar}$), 121.6 ($C_{p\text{-BPh4}}$), 97.6 (CCAu), 83.8 (C_q), 81.5 (C_q), 62.8 (C_q), 58.0 (C_q), 42.1 (CH$_2$), 41.9 (CH$_2$), 31.5, 31.3, 29.8, 29.3, 29.1, 28.8, 26.9, 26.5, 23.0, 22.9, 9.66, 8.97. HRMS (ESI-TOFMS): m/z calculated for $[C_{46}H_{70}AuN_2]^+$ 847.5199, found 847.5197.

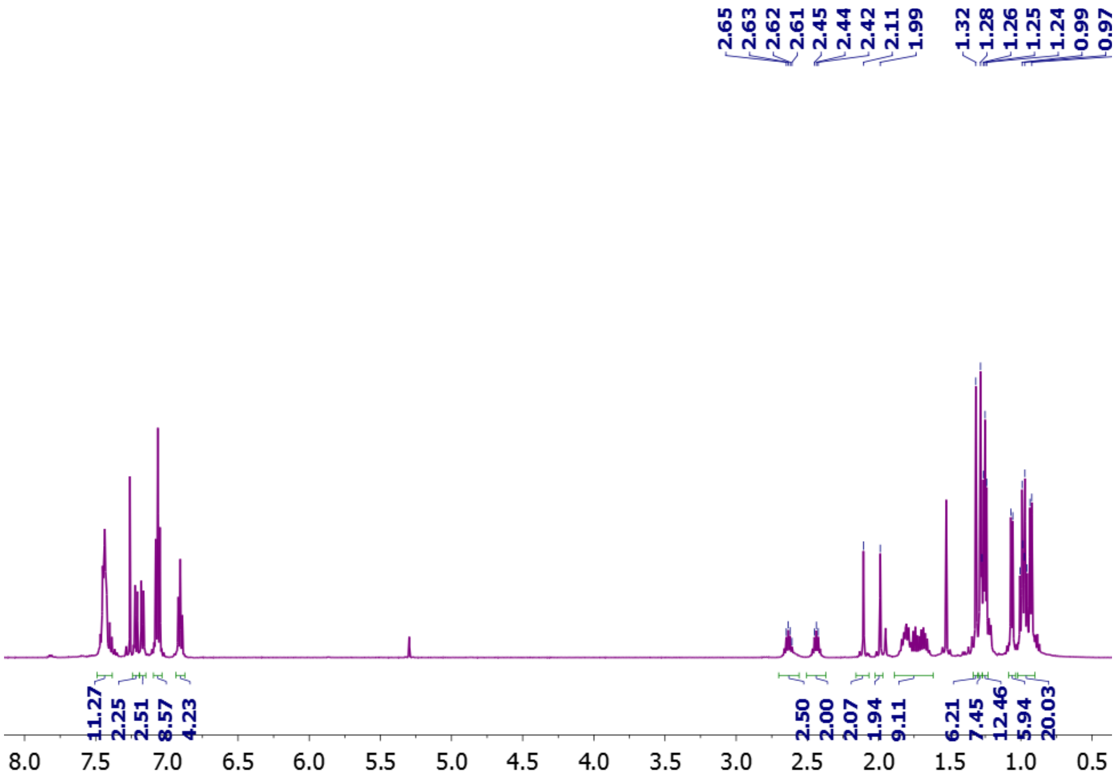

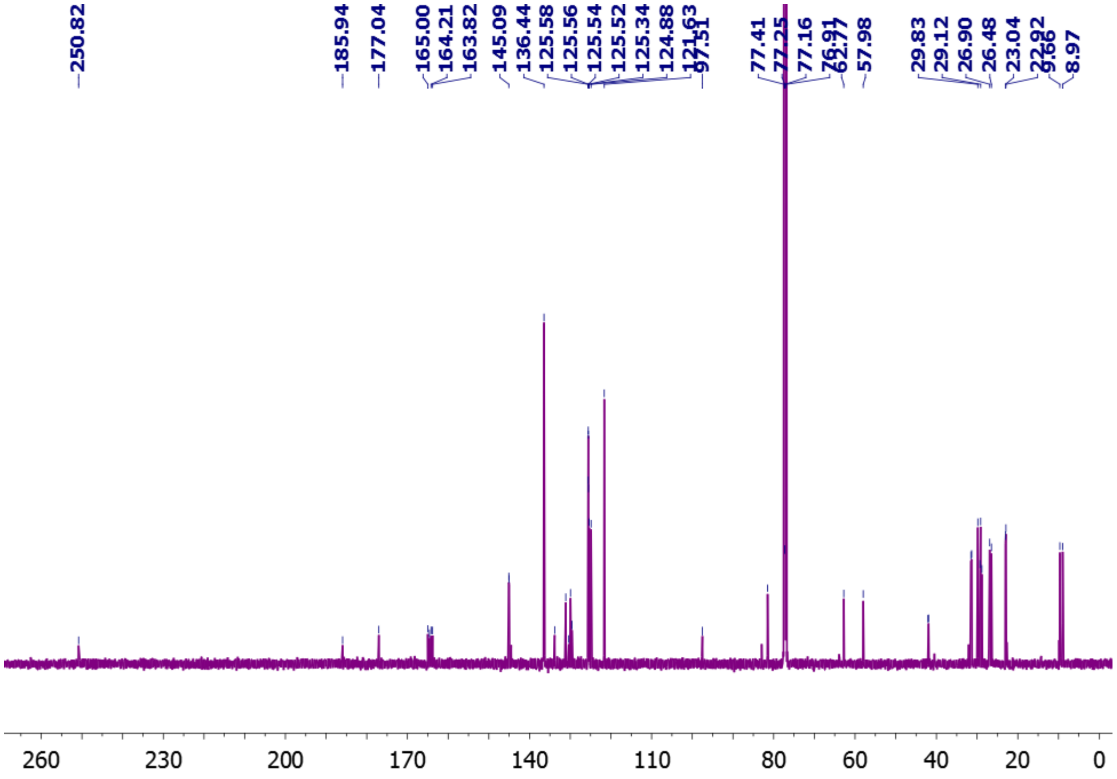

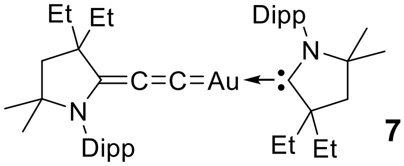

7

THF (10 mL) was added to a mixture of KC_8 (22 mg, 0.16 mmol) and **6b** (150 mg, 0.16 mmol) at room temperature. The dark mixture was stirred at room temperature for 2 hours. The solvent was evaporated under vacuum, and the residue was extracted with benzene. Evaporation of the filtrate under vacuum led to the isolation of **7** as a dark green solid. Yield 53 % (72 mg). Single crystals suitable for X-ray diffraction analysis were obtained from a concentrated tetrahydrofuran solution at -20 ^{o}C.

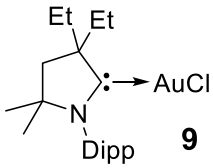

9

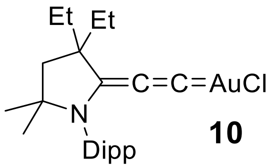

10

Dichloromethane (20 mL) was added to a mixture of **6a** (1.0 g, 1.1 mmol), AuCl(THT) (343 mg, 1.1 mmol), and tetrabutylammonium chloride (300 mg, 2.2 mmol). The reaction mixture was stirred for 15 minutes at room temperature. The volatiles were removed under vacuum and the residue was washed with diethyl ether (3 x 10 mL) and benzene (3 x 10 mL), affording complex **10** as an off-white solid. Yield 74 % (430 mg). m.p. 132.4 °C (dec.). Evaporation of the diethyl ether/benzene fraction and washing with hexanes afforded complex **9** as colorless solid 82 % (478mg). m.p. 153.0 °C.

Complex 9: ^{1}H NMR ($CDCl_3$, 500 MHz): δ = 7.2 (t, *J* = 7.6 Hz, 1 H, *p*-H), 7.26 (d, *J* = 7.6 Hz, 2 H, *m*-H), 2.79 (sept, *J* = 6.8 Hz, 2 H, C*H*Me$_2$), 2.07 (s, 2 H, C*H*$_2$), 1.92 (m, 2 H, C*H*$_2$CH$_3$), 1.87 (m, 2 H, C*H*$_2$CH$_3$), 1.40 (d, *J* = 6.8 Hz, 6 H, CHC*H*$_3$), 1.37 (s, 6 H, C*H*$_3$), 1.30 (d, *J* = 6.8 Hz, 6 H, CHC*H*$_3$), 1.07 (t, *J* = 7.7 Hz, 6 H, CH$_2$C*H*$_3$); ^{13}C NMR ($CDCl_3$, 125 MHz): δ = 237.2 (*C*Au), 145.1($C_{q\text{-}Ar}$), 134.2 ($C_{q\text{-}Ar}$), 130.0 (CH_{Ar}), 125.1 (CH_{Ar}), 80.6 (C_q), 62.4 (C_q), 41.3 (CH_2), 31.8 (CH_2), 29.4, 29.3, 27.3, 22.8, 9.6. HRMS (ESI-TOFMS): m/z calculated for $[C_{22}H_{36}NAuCl]^+$ 546.2196, found 546.2192.

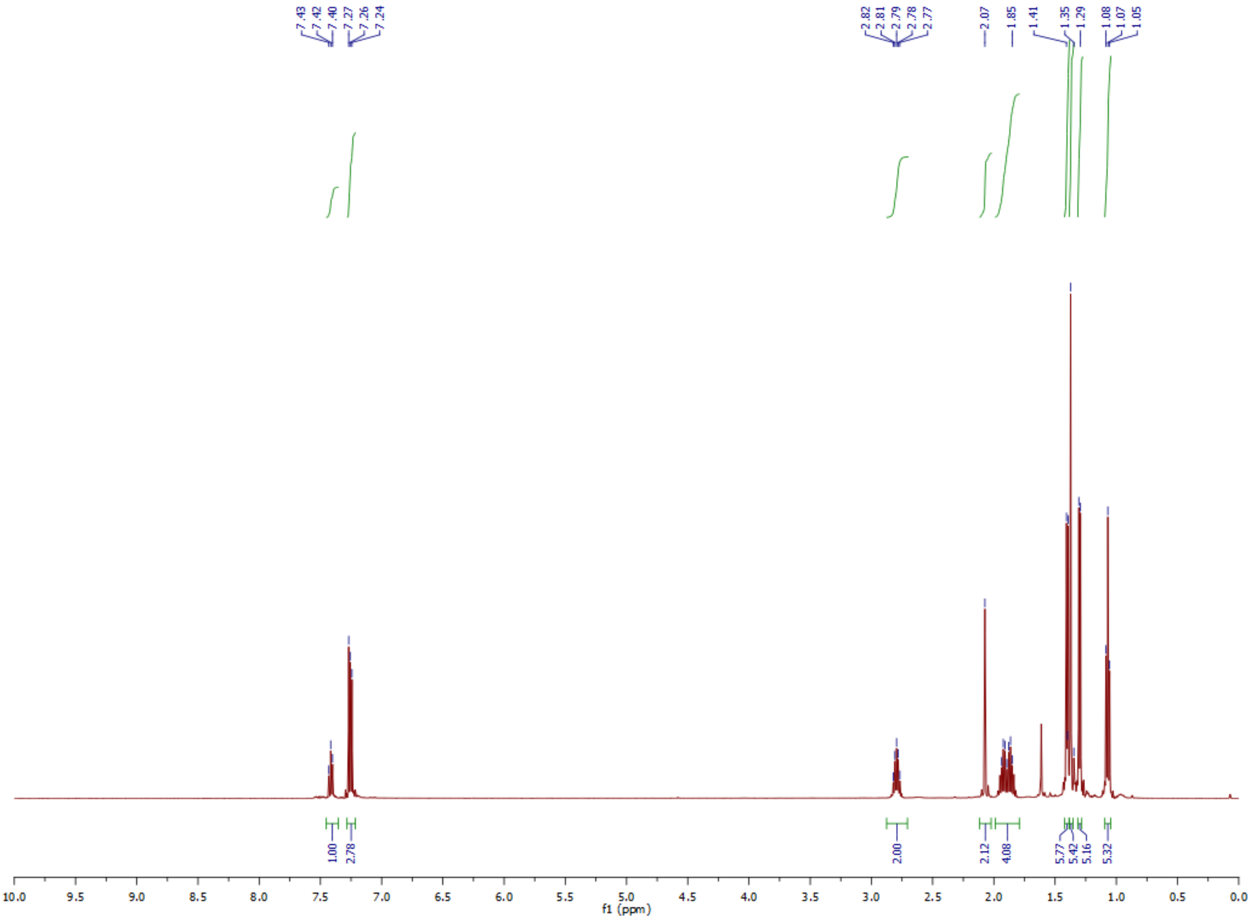

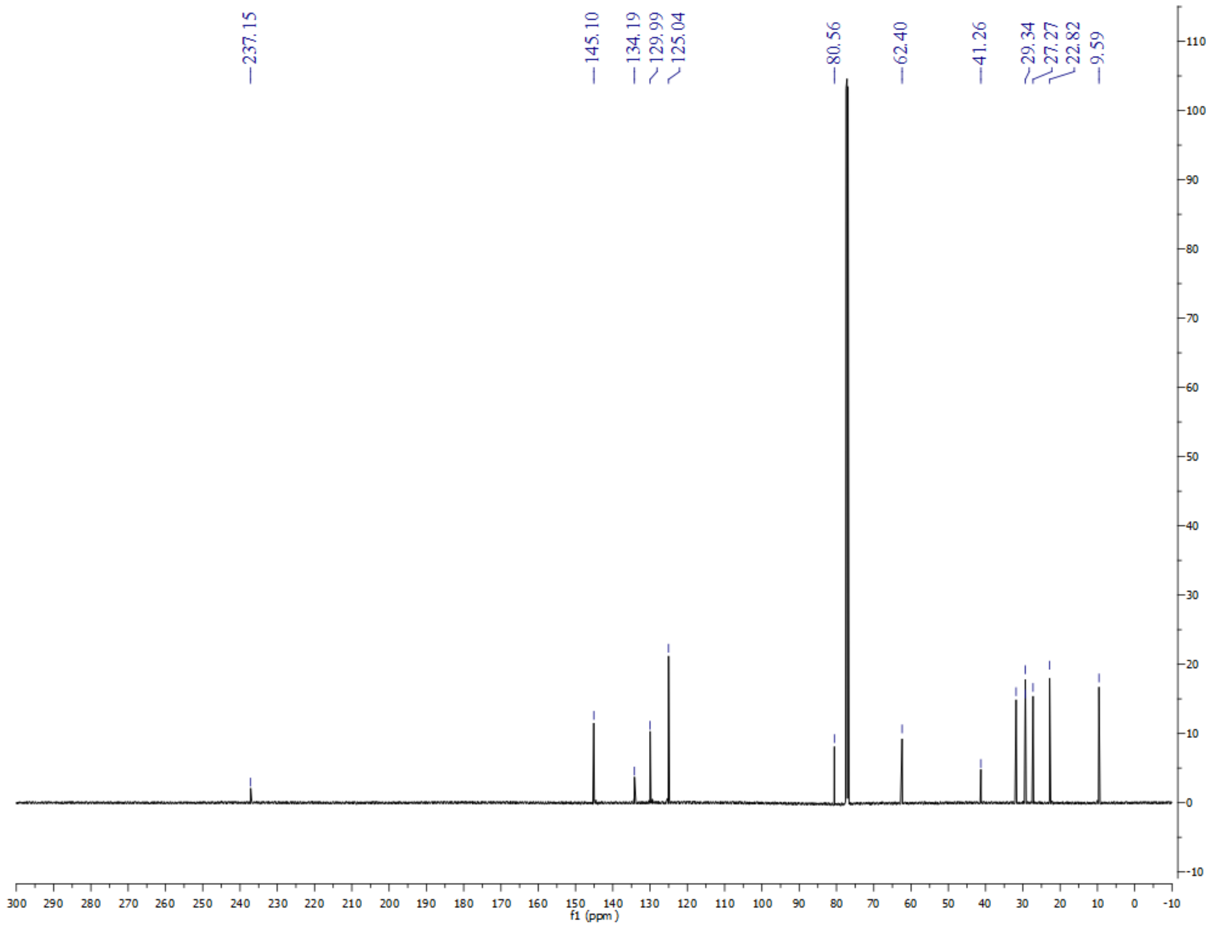

Complex 10: ^{1}H NMR ($CDCl_3$, 500 MHz): δ = 7.50 (t, *J* = 7.8 Hz, 1 H, *p*-H), 7.31 (d, *J* = 7.8 Hz, 2 H, *m*-H), 2.62 (sept, *J* = 6.9 Hz, 2 H, C*H*Me$_2$), 2.27 (s, 2 H, CH_2), 2.01-1.86 (m, 4 H, CH_2CH_3), 1.43 (s, 6 H, CH_3), 1.30 (d, *J* = 6.9 Hz, 6 H, CHCH_3), 1.23 (d, *J* = 6.9 Hz, 6 H, CHCH_3), 1.09 (t, *J* = 7.5 Hz, 6 H, CH$_2$CH_3); ^{13}C NMR ($CDCl_3$, 125 MHz): δ = 177.3 and 173.9 (*C*Au and *C*CCAu), 145.4 (C_q), 131.3 (CH_{Ar}), 129.7 (C_q), 125.5 (CH_{Ar}), 92.3 (C*C*CAu), 75.9 (C_q), 58.2 (C_q), 42.3 (CH_2), 31.7 (CH_2), 30.0, 29.0, 26.7, 23.0, 9.0. HRMS (ESI-TOFMS): m/z calculated for $[C_{24}H_{36}NAuCl]^+$ 570.2196, found 570.2199.

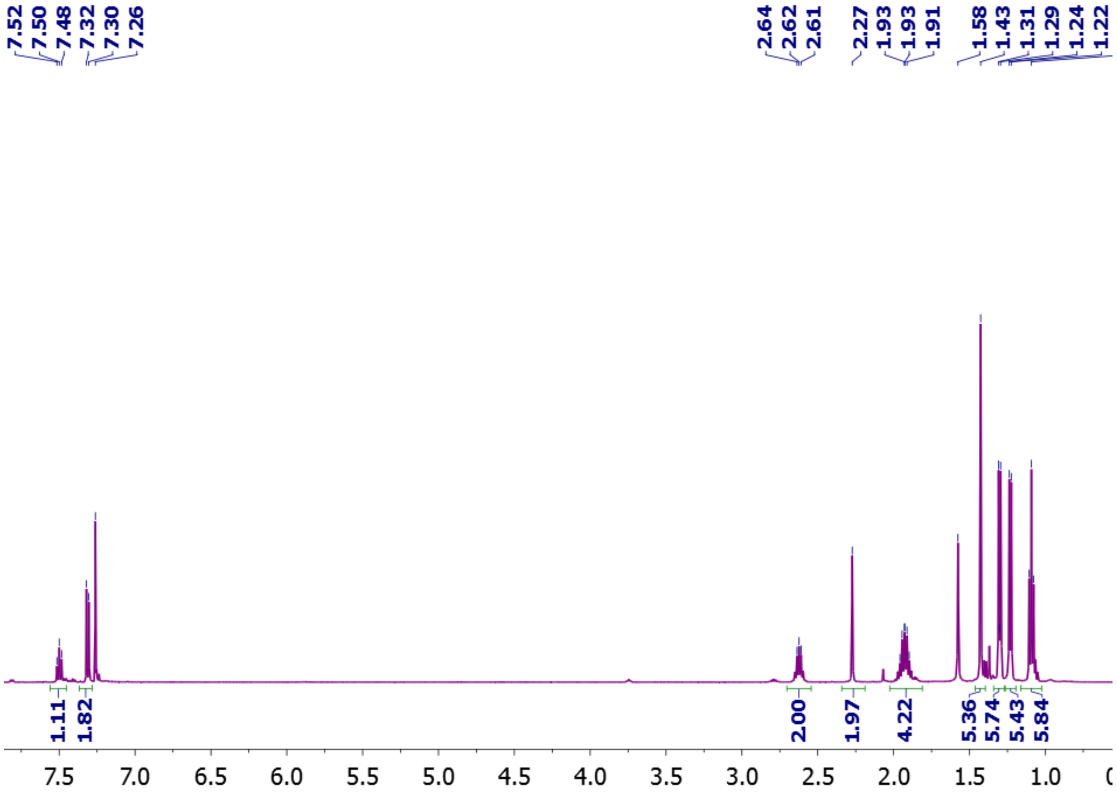

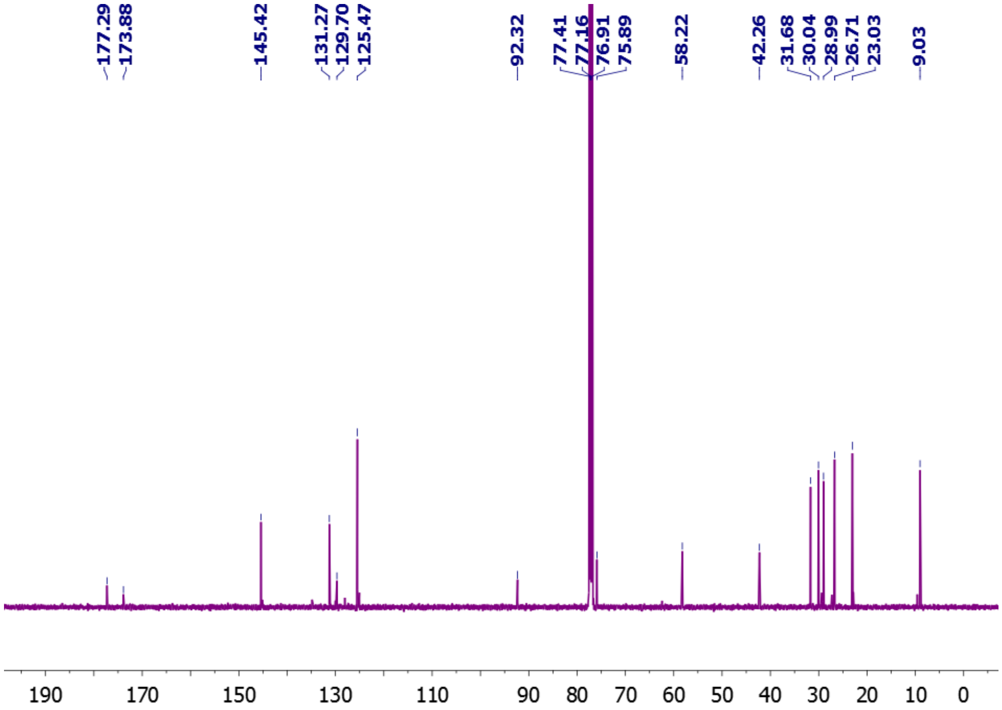

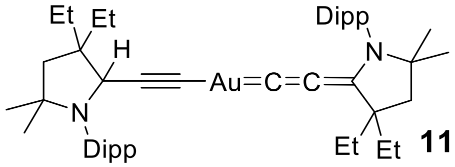

11

Complex **10** (430 mg, 0.75 mmol) was added to a solution of compound **2** (305 mg, 0.9 mmol)

and NaOH (323 mg, 8 mmol) in methanol (10 mL). The mixture was stirred for 12 hours at room temperature while a white solid precipitated. After filtration, the white solid was extracted with dichloromethane (10 mL). After removal of volatiles under vacuum, complex **11** was obtained as a white solid. Yield: 76% (500 mg). m.p. 177.3 °C (dec.)

^{1}H NMR ($CDCl_3$, 500 MHz): δ = 7.39 (t, *J* = 7.8 Hz, 1 H, *p*-H), 7.17 (dd, br, 2 H, *m*-H), 7.09 (t, *J* = 7.8 Hz, 1 H, *m*-H), 6.97 (d, *J* = 7.8 Hz, 2 H, *p*-H), 4.57 (s, 1H, C*H*CC), 3.75 (sept, *J* = 6.9 Hz, 1 H, C*H*Me$_2$), 3.23 (sept, *J* = 6.9 Hz, 1 H, C*H*Me$_2$), 2.76-2.58 (m, 2 H, C*H*Me$_2$), 1.92 (s, 2 H, C*H*$_2$), 1.88-1.65 (m, 6 H, C*H*$_2$CH$_3$, C*H*$_2$), 1.58-1.41 (m, 4 H, C*H*$_2$CH$_3$, C*H*$_2$), 1.28 (br, 6 H, C*H*$_3$), 1.26-1.19 (m, 9 H, CHC*H*$_3$, C*H*$_3$), 1.20-1.08 (m, 12 H, CHC*H*$_3$), 1.02 (s, 3 H, C*H*$_3$), 0.97-0.81 (m, 18 H, C*H*$_3$, CH$_2$C*H*$_3$); ^{13}C{^{1}H} NMR ($CDCl_3$, 125 MHz): δ = 200.7 and 176.5 (*C*CCAu and CC*C*Au), 153.6 (*C*$_q$), 151.8 (*C*$_q$), 145.5 (*C*$_q$), 138.6 (*C*$_q$), 131.0 (*C*H$_{Ar}$), 129.8 (Au*C*), 125.8 (*C*H$_{Ar}$), 125.3 (*C*H$_{Ar}$), 123.6 (*C*H$_{Ar}$), 123.3 (*C*H$_{Ar}$), 122.2 (*C*$_q$), 104.9 (AuC*C*), 96.4 (*C*HCCAu), 75.1 (*C*$_q$), 65.7 (*C*H), 61.0 (*C*$_q$), 58.1 (*C*$_q$), 49.2 (*C*H$_2$), 46.0, 42.1 (*C*H$_2$), 31.7, 30.9, 30.8, 29.9, 29.7, 28.9, 28.6, 27.7, 27.4, 27.1, 26.8, 26.4, 24.1, 23.4, 22.9, 9.7, 9.0. HRMS (ESI-TOFMS): m/z calculated for $[C_{48}H_{72}AuN_2]^+$ 873.5356, found 873.5354.

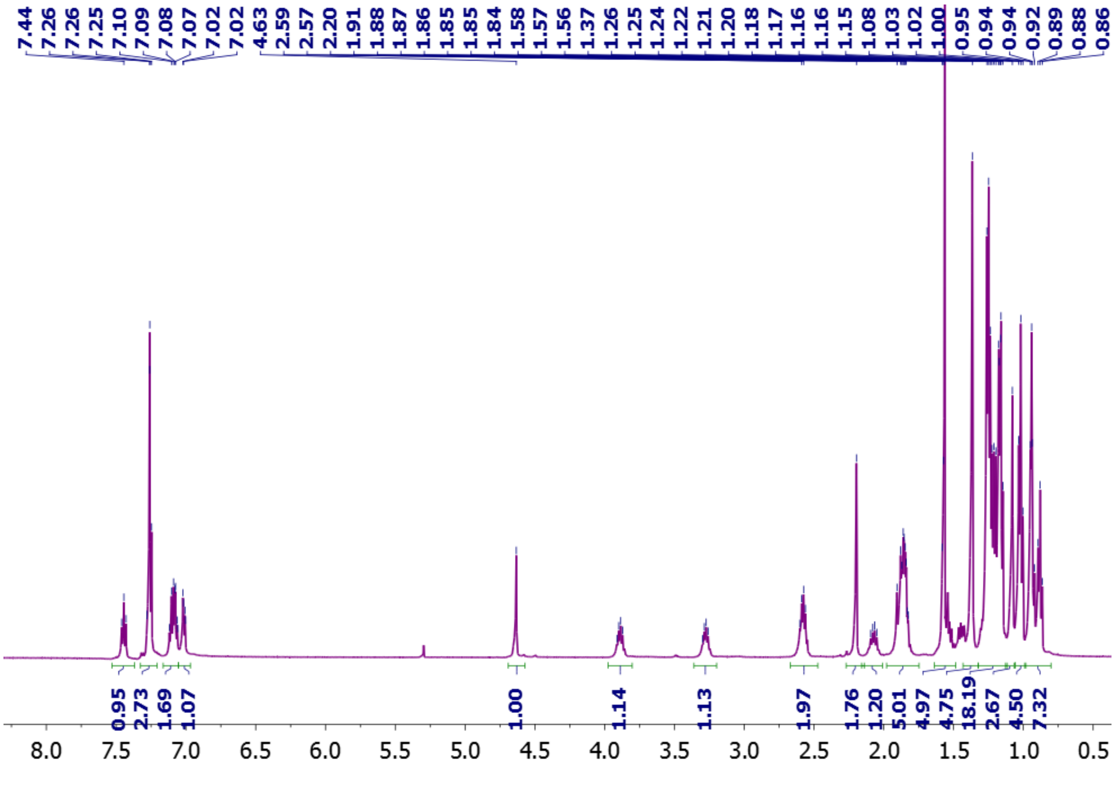

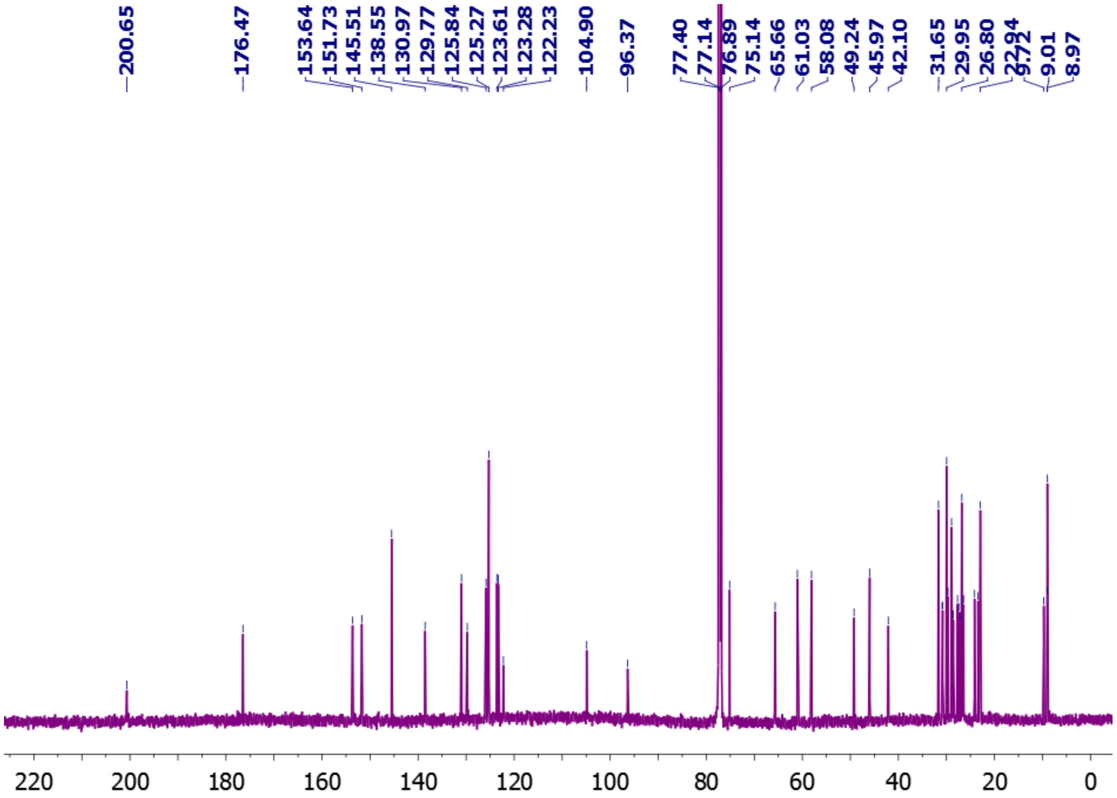

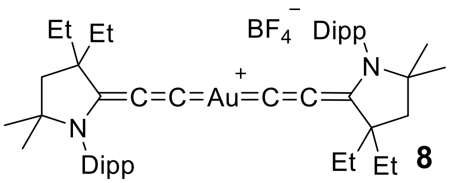

8

Dichloromethane (20 mL) was added to a mixture of **11** (1.0 g, 1.1 mmol) and 2,3-dichloro-5,6-dicyano-1,4-benzoquinone (275 mg, 1.2 mmol). The reaction mixture was stirred for 30 minutes at room temperature. Tetrafluoroboric acid diethyl ether complex was added drop by drop until the dark red color faded to pale yellow. After filtration, the volatiles were removed under vacuum and the residue was washed with benzene (10 mL). The resulting solid was further washed with THF (3 x 10 mL) to afford **8** as an off-white solid. Yield 76 % (830 mg). m.p. 185.1 ^{o}C (dec.). Single crystals suitable for X-ray diffraction analysis were obtained by slow evaporation of a saturated THF solution of **8** at room temperature.

^{1}H NMR ($CDCl_3$, 500 MHz): δ = 7.48 (t, *J* = 7.8 Hz, 2 H, *p*-H), 7.29 (d, *J* = 7.8 Hz, 4 H, *m*-H), 2.59 (sept, *J* = 6.9 Hz, 4 H, C*H*Me$_2$), 2.34 (s, 4 H, C*H*$_2$), 1.96-1.79 (m, 8 H, C*H*$_2$), 1.44 (s, 12 H, C*H*$_3$), 1.29 (d, *J* = 6.9 Hz, 12 H, CHC*H*$_3$), 1.18 (d, *J* = 6.9 Hz, 12 H, CHC*H*$_3$), 1.06 (t, *J* = 7.5 Hz, 12 H, C*H*$_3$); ^{13}C NMR ($CDCl_3$, 125 MHz): δ = 185.8 and 177.7 (*C*Au and *C*CAu), 145.3 (C_q), 131.2 (CH_{Ar}), 129.7 (C_q), 125.4 (CH_{Ar}), 95.9 (C*C*CAu), 77.6 (C_q), 58.6 (C_q), 41.6 (CH_2), 31.7 (CH_2), 29.9, 28.8, 26.7, 22.9, 9.0. HRMS (ESI-TOFMS): m/z calculated for $[C_{48}H_{70}AuN_2]^+$ 871.5199, found 871.5200.

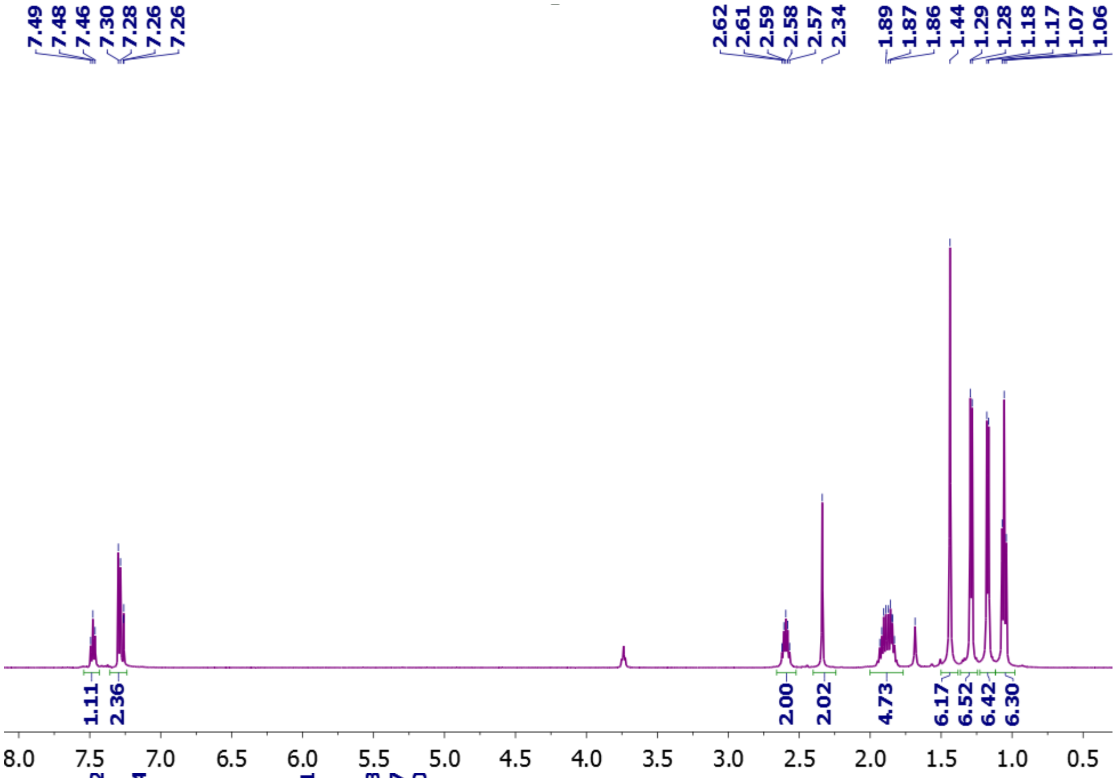
7.49
7.48
7.46
7.30
7.28
7.26
7.26
2.62
2.61
2.59
2.58
2.57
2.34
1.89
1.87
1.86
1.44
1.29
1.28
1.18
1.17
1.07
1.06
1.11
2.36
2.00
2.02
4.73
6.17
6.52
6.42
6.30
8.0 7.5 7.0 6.5 6.0 5.5 5.0 4.5 4.0 3.5 3.0 2.5 2.0 1.5 1.0 0.5

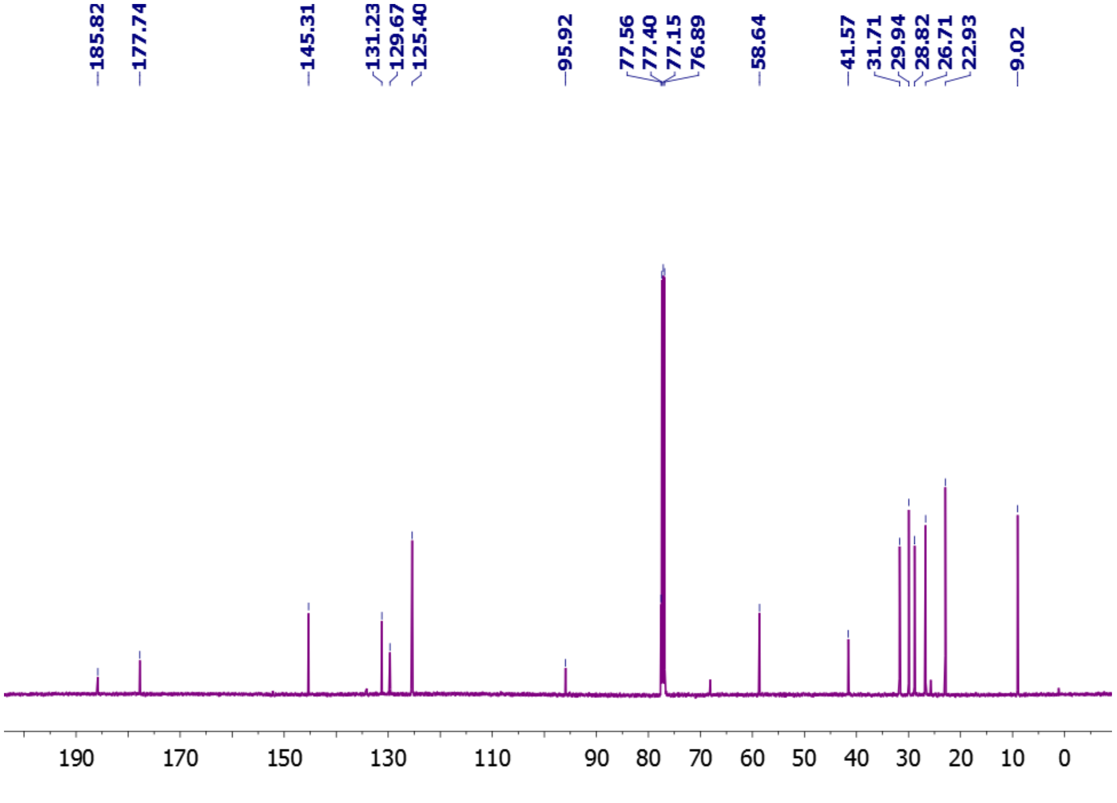
185.82
177.74
145.31
131.23
129.67
125.40
95.92
77.56
77.40
77.15
76.89
58.64
41.57
31.71
29.94
28.82
26.71
22.93
9.02
190 170 150 130 110 90 80 70 60 50 40 30 20 10 0

3. Electrochemistry details

Cyclic voltammetry measurements were performed at room temperature under argon. A freshly polished Pt disk working electrode, a Pt wire as counter electrode, and a Ag/Ag^+ pseudo-reference electrode, with [nBu$_4$N][PF$_6$] (0.1 M) as supporting electrolyte in THF were used. Potentials were calibrated against the Fc/Fc^+ couple.

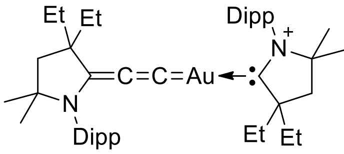

6 (BF$_4$)

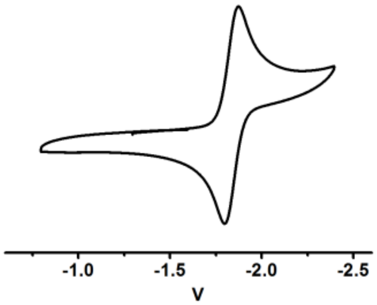

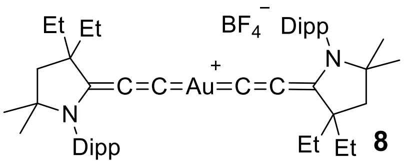

8

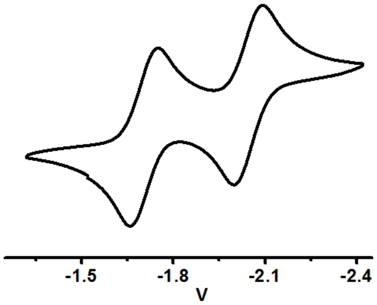

4. EPR Spectroscopy

The EPR spectra were recorded on an X-band spectrometer (Bruker EMX X-band (ν = 9.4 GHz) digital EPR spectrometer) equipped with an NMR gaussmeter and microwave frequency counter. The microwave power and magnetic field modulation amplitude were typically 5.7 mW and 0.32 G, respectively.

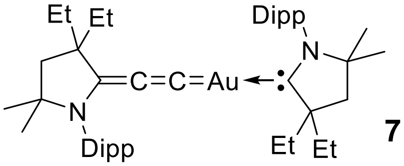

7

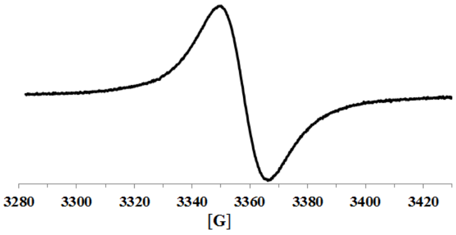

5. Crystallographic details

Table S1. Crystal data and structure refinement for complex **6a (BF_4)**.

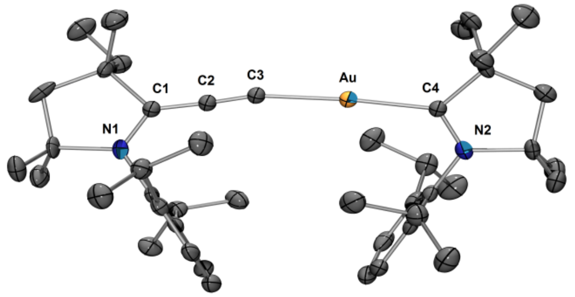

Identification code	**6a (BF_4)** (CCDC 1418631)	
Empirical formula	C58 H94 Au B F4 N2 O3	
Molecular formula	C46 H70 Au N2, B F4, 3(C4 H8 O)	
Temperature	100 K	
Wavelength	0.71073 Å	
Crystal system	Triclinic	
Space group	P-1	
Unit cell dimensions	a = 12.4983(8) Å	a= 98.507(2)°.
	b = 12.6057(9) Å	b= 92.5170(19)°.
	c = 18.1384(12) Å	g = 95.0840(19)°.
Volume	2810.4(3) $Å^3$	
Density (calculated)	1.360 Mg/m^3	
Absorption coefficient	2.674 mm^{-1}	
F(000)	1200	
Crystal size	0.12 x 0.091 x 0.082 mm^3	
Crystal color, habit	Colorless Plate	
Theta range for data collection	1.137 to 25.429°.	
Index ranges	-15<=h<=15, -15<=k<=15, -21<=l<=21	
Reflections collected	52379	
Independent reflections	10333 [R(int) = 0.0466]	
Completeness to theta = 25.000°	100.0 %	
Absorption correction	Semi-empirical from equivalents	
Max. and min. transmission	0.0916 and 0.0674	
Refinement method	Full-matrix least-squares on F^2	
Data / restraints / parameters	10333 / 32 / 624	
Goodness-of-fit on F^2	1.212	
Final R indices [I>2sigma(I)]	R1 = 0.0671, wR2 = 0.1527	
R indices (all data)	R1 = 0.0793, wR2 = 0.1577	
Extinction coefficient	n/a	
Largest diff. peak and hole	1.292 and -1.659 e.$Å^{-3}$	

Table S2 Bond lengths [Å] and angles [°] for **6a (BF_4)**.

Au(1)-C(1)	1.980(6)	Au(1B)-C(1')	2.033(6)	N(1)-C(1)	1.310(8)
Au(1)-C(2)	1.993(15)	Au(1B)-C(2B)	2.08(2)	N(1)-C(7)	1.533(7)

N(1)-C(14)	1.454(8)	C(10)-H(10C)	0.9900	C(17)-H(17)	0.9500
N(1')-C(1')	1.300(8)	C(10)-H(10D)	0.9900	C(17)-C(18)	1.398(9)
N(1')-C(7')	1.539(8)	C(10)-C(11)	1.528(9)	C(17')-H(17')	0.9500
N(1')-C(14')	1.451(8)	C(10')-H(10A)	0.9900	C(17')-C(18')	1.390(10)
C(1)-C(5)	1.576(8)	C(10')-H(10B)	0.9900	C(18)-H(18)	0.9500
C(1)-C(3B)	1.28(2)	C(10')-C(11')	1.515(10)	C(18)-C(19)	1.390(9)
C(1')-C(5')	1.551(8)	C(11)-H(11D)	0.9800	C(18')-H(18')	0.9500
C(1')-C(3)	1.349(16)	C(11)-H(11E)	0.9800	C(18')-C(19')	1.395(9)
C(5)-C(6)	1.596(10)	C(11)-H(11F)	0.9800	C(19)-C(23)	1.529(9)
C(5)-C(10)	1.555(9)	C(11')-H(11A)	0.9800	C(19')-C(23')	1.513(9)
C(5)-C(12)	1.546(9)	C(11')-H(11B)	0.9800	C(20)-H(20)	1.0000
C(5')-C(6')	1.555(9)	C(11')-H(11C)	0.9800	C(20)-C(21)	1.522(9)
C(5')-C(10')	1.553(9)	C(12)-H(12C)	0.9900	C(20)-C(22)	1.539(9)
C(5')-C(12')	1.543(10)	C(12)-H(12D)	0.9900	C(20')-H(20')	1.0000
C(6)-H(6A)	0.9900	C(12)-C(13)	1.514(10)	C(20')-C(21')	1.537(10)
C(6)-H(6B)	0.9900	C(12')-H(12A)	0.9900	C(20')-C(22')	1.535(10)
C(6)-C(7)	1.529(9)	C(12')-H(12B)	0.9900	C(21)-H(21A)	0.9800
C(6')-H(6'A)	0.9900	C(12')-C(13')	1.530(9)	C(21)-H(21B)	0.9800
C(6')-H(6'B)	0.9900	C(13)-H(13D)	0.9800	C(21)-H(21C)	0.9800
C(6')-C(7')	1.546(9)	C(13)-H(13E)	0.9800	C(21')-H(21D)	0.9800
C(7)-C(8)	1.511(9)	C(13)-H(13F)	0.9800	C(21')-H(21E)	0.9800
C(7)-C(9)	1.526(9)	C(13')-H(13A)	0.9800	C(21')-H(21F)	0.9800
C(7')-C(8')	1.527(10)	C(13')-H(13B)	0.9800	C(22)-H(22A)	0.9800
C(7')-C(9')	1.522(10)	C(13')-H(13C)	0.9800	C(22)-H(22B)	0.9800
C(8)-H(8A)	0.9800	C(14)-C(15)	1.421(9)	C(22)-H(22C)	0.9800
C(8)-H(8B)	0.9800	C(14)-C(19)	1.399(8)	C(22')-H(22D)	0.9800
C(8)-H(8C)	0.9800	C(14')-C(15')	1.407(9)	C(22')-H(22E)	0.9800
C(8')-H(8'A)	0.9800	C(14')-C(19')	1.406(9)	C(22')-H(22F)	0.9800
C(8')-H(8'B)	0.9800	C(15)-C(16)	1.392(9)	C(23)-H(23)	1.0000
C(8')-H(8'C)	0.9800	C(15)-C(20)	1.522(9)	C(23)-C(24)	1.534(9)
C(9)-H(9A)	0.9800	C(15')-C(16')	1.386(10)	C(23)-C(25)	1.536(9)
C(9)-H(9B)	0.9800	C(15')-C(20')	1.528(10)	C(23')-H(23')	1.0000
C(9)-H(9C)	0.9800	C(16)-H(16)	0.9500	C(23')-C(24')	1.541(9)
C(9')-H(9'A)	0.9800	C(16)-C(17)	1.359(9)	C(23')-C(25')	1.521(10)
C(9')-H(9'B)	0.9800	C(16')-H(16')	0.9500	C(24)-H(24D)	0.9800
C(9')-H(9'C)	0.9800	C(16')-C(17')	1.372(11)	C(24)-H(24E)	0.9800

C(24)-H(24F)	0.9800	C(6S)-C(7S)	1.495(15)	C(5')-C(1')-Au(1B)	126.2(4)
C(24')-H(24A)	0.9800	C(7S)-H(7SA)	0.9900	C(3)-C(1')-C(5')	127.0(7)
C(24')-H(24B)	0.9800	C(7S)-H(7SB)	0.9900	C(1)-C(5)-C(6)	105.2(5)
C(24')-H(24C)	0.9800	C(7S)-C(8S)	1.547(15)	C(10)-C(5)-C(1)	104.7(5)
C(25)-H(25D)	0.9800	C(8S)-H(8SA)	0.9900	C(10)-C(5)-C(6)	112.3(5)
C(25)-H(25E)	0.9800	C(8S)-H(8SB)	0.9900	C(12)-C(5)-C(1)	109.9(5)
C(25)-H(25F)	0.9800	O(3S)-C(9S)	1.475(17)	C(12)-C(5)-C(6)	113.5(5)
C(25')-H(25A)	0.9800	O(3S)-C(12S)	1.45(2)	C(12)-C(5)-C(10)	110.8(5)
C(25')-H(25B)	0.9800	C(9S)-H(9SA)	0.9900	C(1')-C(5')-C(6')	103.9(5)
C(25')-H(25C)	0.9800	C(9S)-H(9SB)	0.9900	C(1')-C(5')-C(10')	109.4(5)
C(2)-C(3)	1.20(2)	C(9S)-C(10S)	1.443(19)	C(10')-C(5')-C(6')	113.7(6)
C(2B)-C(3B)	1.13(3)	C(10S)-H(10E)	0.9900	C(12')-C(5')-C(1')	104.8(5)
F(1)-B(1)	1.365(12)	C(10S)-H(10F)	0.9900	C(12')-C(5')-C(6')	112.8(6)
F(2)-B(1)	1.381(13)	C(10S)-C(11S)	1.416(14)	C(12')-C(5')-C(10')	111.5(6)
F(3)-B(1)	1.328(13)	C(11S)-H(11G)	0.9900	C(5)-C(6)-H(6A)	110.7
F(4)-B(1)	1.476(15)	C(11S)-H(11H)	0.9900	C(5)-C(6)-H(6B)	110.7
O(1S)-C(1S)	1.414(15)	C(11S)-C(12S)	1.428(16)	H(6A)-C(6)-H(6B)	108.8
O(1S)-C(4S)	1.423(17)	C(12S)-H(12E)	0.9900	C(7)-C(6)-C(5)	105.2(5)
C(1S)-H(1SA)	0.9900	C(12S)-H(12F)	0.9900	C(7)-C(6)-H(6A)	110.7
C(1S)-H(1SB)	0.9900			C(7)-C(6)-H(6B)	110.7
C(1S)-C(2S)	1.493(19)	C(1)-Au(1)-C(2)	176.7(4)	C(5')-C(6')-H(6'A)	110.4
C(2S)-H(2SA)	0.9900	C(1')-Au(1B)-C(2B)	175.7(6)	C(5')-C(6')-H(6'B)	110.4
C(2S)-H(2SB)	0.9900	C(1)-N(1)-C(7)	118.3(5)	H(6'A)-C(6')-H(6'B)	108.6
C(2S)-C(3S)	1.549(18)	C(1)-N(1)-C(14)	118.5(5)	C(7')-C(6')-C(5')	106.7(5)
C(3S)-H(3SA)	0.9900	C(14)-N(1)-C(7)	123.2(5)	C(7')-C(6')-H(6'A)	110.4
C(3S)-H(3SB)	0.9900	C(1')-N(1')-C(7')	116.1(5)	C(7')-C(6')-H(6'B)	110.4
C(3S)-C(4S)	1.52(2)	C(1')-N(1')-C(14')	120.7(5)	C(6)-C(7)-N(1)	102.6(5)
C(4S)-H(4SA)	0.9900	C(14')-N(1')-C(7')	123.2(5)	C(8)-C(7)-N(1)	111.4(5)
C(4S)-H(4SB)	0.9900	N(1)-C(1)-Au(1)	127.8(4)	C(8)-C(7)-C(6)	112.4(6)
O(2S)-C(5S)	1.371(13)	N(1)-C(1)-C(5)	106.2(5)	C(8)-C(7)-C(9)	109.4(6)
O(2S)-C(8S)	1.396(14)	C(5)-C(1)-Au(1)	126.0(4)	C(9)-C(7)-N(1)	109.3(5)
C(5S)-H(5SA)	0.9900	C(3B)-C(1)-N(1)	128.9(9)	C(9)-C(7)-C(6)	111.5(6)
C(5S)-H(5SB)	0.9900	C(3B)-C(1)-C(5)	124.7(9)	N(1')-C(7')-C(6')	101.2(5)
C(5S)-C(6S)	1.463(17)	N(1')-C(1')-Au(1B)	124.6(4)	C(8')-C(7')-N(1')	109.2(5)
C(6S)-H(6SA)	0.9900	N(1')-C(1')-C(5')	109.2(5)	C(8')-C(7')-C(6')	113.0(6)
C(6S)-H(6SB)	0.9900	N(1')-C(1')-C(3)	123.7(7)	C(9')-C(7')-N(1')	110.0(5)

C(9')-C(7')-C(6')	112.9(6)	C(11')-C(10')-H(10A)	108.7	H(13A)-C(13')-H(13C)	109.5
C(9')-C(7')-C(8')	110.1(6)	C(11')-C(10')-H(10B)	108.7	H(13B)-C(13')-H(13C)	109.5
C(7)-C(8)-H(8A)	109.5	C(10)-C(11)-H(11D)	109.5	C(15)-C(14)-N(1)	119.4(5)
C(7)-C(8)-H(8B)	109.5	C(10)-C(11)-H(11E)	109.5	C(19)-C(14)-N(1)	118.7(5)
C(7)-C(8)-H(8C)	109.5	C(10)-C(11)-H(11F)	109.5	C(19)-C(14)-C(15)	121.8(6)
H(8A)-C(8)-H(8B)	109.5	H(11D)-C(11)-H(11E)	109.5	C(15')-C(14')-N(1')	118.3(6)
H(8A)-C(8)-H(8C)	109.5	H(11D)-C(11)-H(11F)	109.5	C(19')-C(14')-N(1')	118.9(5)
H(8B)-C(8)-H(8C)	109.5	H(11E)-C(11)-H(11F)	109.5	C(19')-C(14')-C(15')	122.7(6)
C(7')-C(8')-H(8'A)	109.5	C(10')-C(11')-H(11A)	109.5	C(14)-C(15)-C(20)	123.5(5)
C(7')-C(8')-H(8'B)	109.5	C(10')-C(11')-H(11B)	109.5	C(16)-C(15)-C(14)	117.1(6)
C(7')-C(8')-H(8'C)	109.5	C(10')-C(11')-H(11C)	109.5	C(16)-C(15)-C(20)	119.1(6)
H(8'A)-C(8')-H(8'B)	109.5	H(11A)-C(11')-H(11B)	109.5	C(14')-C(15')-C(20')	123.2(6)
H(8'A)-C(8')-H(8'C)	109.5	H(11A)-C(11')-H(11C)	109.5	C(16')-C(15')-C(14')	117.0(6)
H(8'B)-C(8')-H(8'C)	109.5	H(11B)-C(11')-H(11C)	109.5	C(16')-C(15')-C(20')	119.5(6)
C(7)-C(9)-H(9A)	109.5	C(5)-C(12)-H(12C)	108.5	C(15)-C(16)-H(16)	118.9
C(7)-C(9)-H(9B)	109.5	C(5)-C(12)-H(12D)	108.5	C(17)-C(16)-C(15)	122.1(6)
C(7)-C(9)-H(9C)	109.5	H(12C)-C(12)-H(12D)	107.5	C(17)-C(16)-H(16)	118.9
H(9A)-C(9)-H(9B)	109.5	C(13)-C(12)-C(5)	115.1(6)	C(15')-C(16')-H(16')	119.1
H(9A)-C(9)-H(9C)	109.5	C(13)-C(12)-H(12C)	108.5	C(17')-C(16')-C(15')	121.8(7)
H(9B)-C(9)-H(9C)	109.5	C(13)-C(12)-H(12D)	108.5	C(17')-C(16')-H(16')	119.1
C(7')-C(9')-H(9'A)	109.5	C(5')-C(12')-H(12A)	108.6	C(16)-C(17)-H(17)	120.0
C(7')-C(9')-H(9'B)	109.5	C(5')-C(12')-H(12B)	108.6	C(16)-C(17)-C(18)	119.9(6)
C(7')-C(9')-H(9'C)	109.5	H(12A)-C(12')-H(12B)	107.6	C(18)-C(17)-H(17)	120.0
H(9'A)-C(9')-H(9'B)	109.5	C(13')-C(12')-C(5')	114.7(6)	C(16')-C(17')-H(17')	119.9
H(9'A)-C(9')-H(9'C)	109.5	C(13')-C(12')-H(12A)	108.6	C(16')-C(17')-C(18')	120.1(6)
H(9'B)-C(9')-H(9'C)	109.5	C(13')-C(12')-H(12B)	108.6	C(18')-C(17')-H(17')	119.9
C(5)-C(10)-H(10C)	108.5	C(12)-C(13)-H(13D)	109.5	C(17)-C(18)-H(18)	119.5
C(5)-C(10)-H(10D)	108.5	C(12)-C(13)-H(13E)	109.5	C(19)-C(18)-C(17)	121.1(6)
H(10C)-C(10)-H(10D)	107.5	C(12)-C(13)-H(13F)	109.5	C(19)-C(18)-H(18)	119.5
C(11)-C(10)-C(5)	115.1(6)	H(13D)-C(13)-H(13E)	109.5	C(17')-C(18')-H(18')	119.5
C(11)-C(10)-H(10C)	108.5	H(13D)-C(13)-H(13F)	109.5	C(17')-C(18')-C(19')	121.1(6)
C(11)-C(10)-H(10D)	108.5	H(13E)-C(13)-H(13F)	109.5	C(19')-C(18')-H(18')	119.5
C(5')-C(10')-H(10A)	108.7	C(12')-C(13')-H(13A)	109.5	C(14)-C(19)-C(23)	122.9(5)
C(5')-C(10')-H(10B)	108.7	C(12')-C(13')-H(13B)	109.5	C(18)-C(19)-C(14)	117.8(6)
H(10A)-C(10')-H(10B)	107.6	C(12')-C(13')-H(13C)	109.5	C(18)-C(19)-C(23)	119.1(6)
C(11')-C(10')-C(5')	114.4(6)	H(13A)-C(13')-H(13B)	109.5	C(14')-C(19')-C(23')	124.1(6)

C(18')-C(19')-C(14')	117.0(6)	H(22D)-C(22')-H(22F)	109.5	H(25A)-C(25')-H(25C)	109.5
C(18')-C(19')-C(23')	118.6(6)	H(22E)-C(22')-H(22F)	109.5	H(25B)-C(25')-H(25C)	109.5
C(15)-C(20)-H(20)	108.2	C(19)-C(23)-H(23)	107.9	C(3)-C(2)-Au(1)	163.8(12)
C(15)-C(20)-C(21)	109.8(5)	C(19)-C(23)-C(24)	110.7(5)	C(2)-C(3)-C(1')	173.9(13)
C(15)-C(20)-C(22)	113.2(5)	C(19)-C(23)-C(25)	112.2(5)	C(3B)-C(2B)-Au(1B)	167(2)
C(21)-C(20)-H(20)	108.2	C(24)-C(23)-H(23)	107.9	C(2B)-C(3B)-C(1)	174(2)
C(21)-C(20)-C(22)	109.2(5)	C(24)-C(23)-C(25)	110.0(5)	F(1)-B(1)-F(2)	110.9(9)
C(22)-C(20)-H(20)	108.2	C(25)-C(23)-H(23)	107.9	F(1)-B(1)-F(4)	107.9(9)
C(15')-C(20')-H(20')	108.1	C(19')-C(23')-H(23')	108.1	F(2)-B(1)-F(4)	105.2(10)
C(15')-C(20')-C(21')	109.3(6)	C(19')-C(23')-C(24')	110.0(6)	F(3)-B(1)-F(1)	109.8(9)
C(15')-C(20')-C(22')	112.3(6)	C(19')-C(23')-C(25')	113.3(6)	F(3)-B(1)-F(2)	115.1(9)
C(21')-C(20')-H(20')	108.1	C(24')-C(23')-H(23')	108.1	F(3)-B(1)-F(4)	107.5(9)
C(22')-C(20')-H(20')	108.1	C(25')-C(23')-H(23')	108.1	C(1S)-O(1S)-C(4S)	104.1(13)
C(22')-C(20')-C(21')	110.9(6)	C(25')-C(23')-C(24')	109.2(6)	O(1S)-C(1S)-H(1SA)	109.0
C(20)-C(21)-H(21A)	109.5	C(23)-C(24)-H(24D)	109.5	O(1S)-C(1S)-H(1SB)	109.0
C(20)-C(21)-H(21B)	109.5	C(23)-C(24)-H(24E)	109.5	O(1S)-C(1S)-C(2S)	112.9(13)
C(20)-C(21)-H(21C)	109.5	C(23)-C(24)-H(24F)	109.5	H(1SA)-C(1S)-H(1SB)	107.8
H(21A)-C(21)-H(21B)	109.5	H(24D)-C(24)-H(24E)	109.5	C(2S)-C(1S)-H(1SA)	109.0
H(21A)-C(21)-H(21C)	109.5	H(24D)-C(24)-H(24F)	109.5	C(2S)-C(1S)-H(1SB)	109.0
H(21B)-C(21)-H(21C)	109.5	H(24E)-C(24)-H(24F)	109.5	C(1S)-C(2S)-H(2SA)	112.5
C(20')-C(21')-H(21D)	109.5	C(23')-C(24')-H(24A)	109.5	C(1S)-C(2S)-H(2SB)	112.5
C(20')-C(21')-H(21E)	109.5	C(23')-C(24')-H(24B)	109.5	C(1S)-C(2S)-C(3S)	96.4(14)
C(20')-C(21')-H(21F)	109.5	C(23')-C(24')-H(24C)	109.5	H(2SA)-C(2S)-H(2SB)	110.0
H(21D)-C(21')-H(21E)	109.5	H(24A)-C(24')-H(24B)	109.5	C(3S)-C(2S)-H(2SA)	112.5
H(21D)-C(21')-H(21F)	109.5	H(24A)-C(24')-H(24C)	109.5	C(3S)-C(2S)-H(2SB)	112.5
H(21E)-C(21')-H(21F)	109.5	H(24B)-C(24')-H(24C)	109.5	C(2S)-C(3S)-H(3SA)	111.3
C(20)-C(22)-H(22A)	109.5	C(23)-C(25)-H(25D)	109.5	C(2S)-C(3S)-H(3SB)	111.3
C(20)-C(22)-H(22B)	109.5	C(23)-C(25)-H(25E)	109.5	H(3SA)-C(3S)-H(3SB)	109.2
C(20)-C(22)-H(22C)	109.5	C(23)-C(25)-H(25F)	109.5	C(4S)-C(3S)-C(2S)	102.4(12)
H(22A)-C(22)-H(22B)	109.5	H(25D)-C(25)-H(25E)	109.5	C(4S)-C(3S)-H(3SA)	111.3
H(22A)-C(22)-H(22C)	109.5	H(25D)-C(25)-H(25F)	109.5	C(4S)-C(3S)-H(3SB)	111.3
H(22B)-C(22)-H(22C)	109.5	H(25E)-C(25)-H(25F)	109.5	O(1S)-C(4S)-C(3S)	109.0(13)
C(20')-C(22')-H(22D)	109.5	C(23')-C(25')-H(25A)	109.5	O(1S)-C(4S)-H(4SA)	109.9
C(20')-C(22')-H(22E)	109.5	C(23')-C(25')-H(25B)	109.5	O(1S)-C(4S)-H(4SB)	109.9
C(20')-C(22')-H(22F)	109.5	C(23')-C(25')-H(25C)	109.5	C(3S)-C(4S)-H(4SA)	109.9
H(22D)-C(22')-H(22E)	109.5	H(25A)-C(25')-H(25B)	109.5	C(3S)-C(4S)-H(4SB)	109.9

H(4SA)-C(4S)-H(4SB)	108.3	H(7SA)-C(7S)-H(7SB)	109.3	C(9S)-C(10S)-H(10F)	110.8
C(5S)-O(2S)-C(8S)	107.5(10)	C(8S)-C(7S)-H(7SA)	111.4	H(10E)-C(10S)-H(10F)	108.9
O(2S)-C(5S)-H(5SA)	109.1	C(8S)-C(7S)-H(7SB)	111.4	C(11S)-C(10S)-C(9S)	104.5(13)
O(2S)-C(5S)-H(5SB)	109.1	O(2S)-C(8S)-C(7S)	103.1(9)	C(11S)-C(10S)-H(10E)	110.8
O(2S)-C(5S)-C(6S)	112.7(11)	O(2S)-C(8S)-H(8SA)	111.2	C(11S)-C(10S)-H(10F)	110.8
H(5SA)-C(5S)-H(5SB)	107.8	O(2S)-C(8S)-H(8SB)	111.2	C(10S)-C(11S)-H(11G)	110.5
C(6S)-C(5S)-H(5SA)	109.1	C(7S)-C(8S)-H(8SA)	111.2	C(10S)-C(11S)-H(11H)	110.5
C(6S)-C(5S)-H(5SB)	109.1	C(7S)-C(8S)-H(8SB)	111.2	C(10S)-C(11S)-C(12S)	106.4(16)
C(5S)-C(6S)-H(6SA)	112.3	H(8SA)-C(8S)-H(8SB)	109.1	H(11G)-C(11S)-H(11H)	108.6
C(5S)-C(6S)-H(6SB)	112.3	C(12S)-O(3S)-C(9S)	103.8(13)	C(12S)-C(11S)-H(11G)	110.5
C(5S)-C(6S)-C(7S)	97.4(11)	O(3S)-C(9S)-H(9SA)	110.1	C(12S)-C(11S)-H(11H)	110.5
H(6SA)-C(6S)-H(6SB)	109.9	O(3S)-C(9S)-H(9SB)	110.1	O(3S)-C(12S)-H(12E)	110.1
C(7S)-C(6S)-H(6SA)	112.3	H(9SA)-C(9S)-H(9SB)	108.4	O(3S)-C(12S)-H(12F)	110.1
C(7S)-C(6S)-H(6SB)	112.3	C(10S)-C(9S)-O(3S)	108.1(13)	C(11S)-C(12S)-O(3S)	108.1(16)
C(6S)-C(7S)-H(7SA)	111.4	C(10S)-C(9S)-H(9SA)	110.1	C(11S)-C(12S)-H(12E)	110.1
C(6S)-C(7S)-H(7SB)	111.4	C(10S)-C(9S)-H(9SB)	110.1	C(11S)-C(12S)-H(12F)	110.1
C(6S)-C(7S)-C(8S)	101.8(9)	C(9S)-C(10S)-H(10E)	110.8	H(12E)-C(12S)-H(12F)	108.4

Table S3. Crystal data and structure refinement for complex **7**.

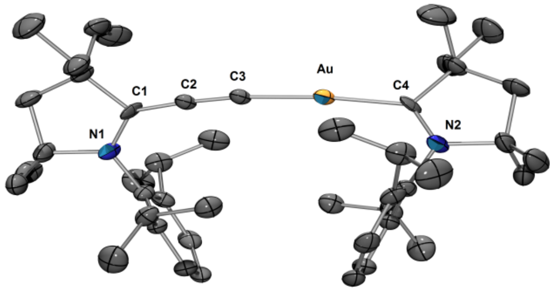

Identification code	**7** (CCDC 1418632)	
Empirical formula	C60 H98 Au N2 O3.50	
Molecular formula	C46 H70 Au N2, 3.5(C4 H8 O)	
Temperature	100 K	
Wavelength	0.71073 Å	
Crystal system	Monoclinic	
Space group	C 1 2/c 1	
Unit cell dimensions	a = 20.0066(6) Å	a= 90°.
	b = 16.2975(5) Å	b= 119.0230(14)°.
	c = 20.6068(6) Å	g = 90°.
Volume	5875.3(3) Å^3	
Density (calculated)	1.244 Mg/m^3	
Absorption coefficient	2.547 mm^{-1}	
F(000)	2316	
Crystal size	0.204 x 0.164 x 0.103 mm^3	
Crystal color, habit	Yellow Block	
Theta range for data collection	2.261 to 25.374°.	
Index ranges	-24<=h<=24, -19<=k<=19, -24<=l<=24	
Reflections collected	35165	
Independent reflections	5391 [R(int) = 0.0557]	
Completeness to theta = 25.374°	99.8 %	
Absorption correction	Semi-empirical from equivalents	
Max. and min. transmission	0.4901 and 0.4123	
Refinement method	Full-matrix least-squares on F^2	
Data / restraints / parameters	5391 / 0 / 231	
Goodness-of-fit on F^2	1.154	
Final R indices [I>2sigma(I)]	R1 = 0.0474, wR2 = 0.1178	
R indices (all data)	R1 = 0.0566, wR2 = 0.1209	
Extinction coefficient	n/a	
Largest diff. peak and hole	0.591 and -1.048 e.Å^{-3}	

Table S4. Bond lengths [Å] and angles [°] for **7**.

Au(1)-Au(1)#1	2.5380(9)	N(1)-C(13)	1.454(5)	C(3)-C(4)	1.543(6)
Au(1)-C(1)#1	0.592(11)	C(1)-Au(1)#1	0.592(11)	C(5)-C(4)	1.549(7)
Au(1)-C(1)	1.951(11)	C(1)-C(1)#1	1.36(2)	C(5)-H(5A)	0.9900
Au(1)-C(2)#1	0.703(11)	C(2)-Au(1)#1	0.703(11)	C(5)-H(5B)	0.9900
Au(1)-C(3)#1	2.003(4)	C(2)-C(1)	1.293(16)	C(5)-C(6)	1.550(6)
N(1)-C(3)	1.308(6)	C(3)-Au(1)#1	2.003(4)	C(6)-C(7)	1.530(7)
N(1)-C(6)	1.533(6)	C(3)-C(2)	1.302(12)	C(6)-C(8)	1.516(8)

C(7)-H(7A)	0.9800	C(24)-H(24B)	0.9800	C(8)-C(6)-C(5)	112.5(4)
C(7)-H(7B)	0.9800	C(24)-H(24C)	0.9800	C(8)-C(6)-C(7)	109.0(4)
C(7)-H(7C)	0.9800			C(6)-C(7)-H(7A)	109.5
C(8)-H(8A)	0.9800	C(1)#1-Au(1)-Au(1)#1	6.7(11)	C(6)-C(7)-H(7B)	109.5
C(8)-H(8B)	0.9800	C(1)-Au(1)-Au(1)#1	2.0(3)	C(6)-C(7)-H(7C)	109.5
C(8)-H(8C)	0.9800	C(1)#1-Au(1)-C(1)	5.6(11)	H(7A)-C(7)-H(7B)	109.5
C(9)-C(4)	1.548(7)	C(1)#1-Au(1)-C(2)#1	174.7(12)	H(7A)-C(7)-H(7C)	109.5
C(9)-H(9A)	0.9900	C(1)#1-Au(1)-C(3)#1	175.0(11)	H(7B)-C(7)-H(7C)	109.5
C(9)-H(9B)	0.9900	C(1)-Au(1)-C(3)#1	174.3(4)	C(6)-C(8)-H(8A)	109.5
C(9)-C(10)	1.532(8)	C(2)#1-Au(1)-Au(1)#1	175.9(8)	C(6)-C(8)-H(8B)	109.5
C(10)-H(10A)	0.9800	C(2)#1-Au(1)-C(1)	177.9(8)	C(6)-C(8)-H(8C)	109.5
C(10)-H(10B)	0.9800	C(2)#1-Au(1)-C(3)#1	3.7(8)	H(8A)-C(8)-H(8B)	109.5
C(10)-H(10C)	0.9800	C(3)#1-Au(1)-Au(1)#1	172.32(12)	H(8A)-C(8)-H(8C)	109.5
C(11)-C(4)	1.554(7)	C(3)-N(1)-C(6)	115.5(3)	H(8B)-C(8)-H(8C)	109.5
C(11)-H(11A)	0.9900	C(3)-N(1)-C(13)	122.4(4)	C(4)-C(9)-H(9A)	108.8
C(11)-H(11B)	0.9900	C(13)-N(1)-C(6)	121.9(3)	C(4)-C(9)-H(9B)	108.8
C(11)-C(12)	1.528(7)	Au(1)#1-C(1)-Au(1)	171.3(15)	H(9A)-C(9)-H(9B)	107.7
C(12)-H(12A)	0.9800	Au(1)#1-C(1)-C(1)#1	172.0(16)	C(10)-C(9)-C(4)	113.8(4)
C(12)-H(12B)	0.9800	Au(1)#1-C(1)-C(2)	2.9(7)	C(10)-C(9)-H(9A)	108.8
C(12)-H(12C)	0.9800	C(1)#1-C(1)-Au(1)	2.4(5)	C(10)-C(9)-H(9B)	108.8
C(14)-C(13)	1.409(6)	C(2)-C(1)-Au(1)	173.3(11)	C(9)-C(10)-H(10A)	109.5
C(14)-C(15)	1.402(6)	C(2)-C(1)-C(1)#1	174.6(10)	C(9)-C(10)-H(10B)	109.5
C(14)-C(19)	1.524(6)	Au(1)#1-C(2)-C(1)	2.4(6)	C(9)-C(10)-H(10C)	109.5
C(15)-H(15)	0.9500	Au(1)#1-C(2)-C(3)	174.2(13)	H(10A)-C(10)-H(10B)	109.5
C(15)-C(16)	1.369(7)	C(1)-C(2)-C(3)	174.8(11)	H(10A)-C(10)-H(10C)	109.5
C(16)-H(16)	0.9500	N(1)-C(3)-Au(1)#1	124.6(3)	H(10B)-C(10)-H(10C)	109.5
C(16)-C(17)	1.385(7)	N(1)-C(3)-C(4)	109.4(4)	C(4)-C(11)-H(11A)	108.6
C(17)-H(17)	0.9500	C(2)-C(3)-Au(1)#1	2.0(4)	C(4)-C(11)-H(11B)	108.6
C(18)-C(13)	1.398(6)	C(2)-C(3)-N(1)	126.5(6)	H(11A)-C(11)-H(11B)	107.5
C(18)-C(17)	1.383(6)	C(2)-C(3)-C(4)	124.2(6)	C(12)-C(11)-C(4)	114.8(5)
C(18)-C(22)	1.527(6)	C(4)-C(3)-Au(1)#1	126.0(3)	C(12)-C(11)-H(11A)	108.6
C(19)-H(19)	1.0000	C(3)-C(4)-C(5)	104.4(4)	C(12)-C(11)-H(11B)	108.6
C(19)-C(20)	1.537(7)	C(3)-C(4)-C(9)	109.9(4)	C(11)-C(12)-H(12A)	109.5
C(19)-C(21)	1.536(7)	C(3)-C(4)-C(11)	105.3(4)	C(11)-C(12)-H(12B)	109.5
C(20)-H(20A)	0.9800	C(5)-C(4)-C(11)	112.8(4)	C(11)-C(12)-H(12C)	109.5
C(20)-H(20B)	0.9800	C(9)-C(4)-C(5)	112.7(4)	H(12A)-C(12)-H(12B)	109.5
C(20)-H(20C)	0.9800	C(9)-C(4)-C(11)	111.1(4)	H(12A)-C(12)-H(12C)	109.5
C(21)-H(21A)	0.9800	C(4)-C(5)-H(5A)	110.5	H(12B)-C(12)-H(12C)	109.5
C(21)-H(21B)	0.9800	C(4)-C(5)-H(5B)	110.5	C(14)-C(13)-N(1)	119.4(4)
C(21)-H(21C)	0.9800	C(4)-C(5)-C(6)	106.3(4)	C(18)-C(13)-N(1)	118.4(4)
C(22)-H(22)	1.0000	H(5A)-C(5)-H(5B)	108.7	C(18)-C(13)-C(14)	122.2(4)
C(22)-C(23)	1.542(6)	C(6)-C(5)-H(5A)	110.5	C(13)-C(14)-C(19)	123.5(4)
C(23)-H(23A)	0.9800	C(6)-C(5)-H(5B)	110.5	C(15)-C(14)-C(13)	116.9(4)
C(23)-H(23B)	0.9800	N(1)-C(6)-C(5)	101.5(4)	C(15)-C(14)-C(19)	119.4(4)
C(23)-H(23C)	0.9800	C(7)-C(6)-N(1)	110.2(4)	C(14)-C(15)-H(15)	119.2
C(24)-C(22)	1.534(7)	C(7)-C(6)-C(5)	113.3(4)	C(16)-C(15)-C(14)	121.7(4)
C(24)-H(24A)	0.9800	C(8)-C(6)-N(1)	110.2(4)	C(16)-C(15)-H(15)	119.2

C(15)-C(16)-H(16)	120.1
C(15)-C(16)-C(17)	119.7(4)
C(17)-C(16)-H(16)	120.1
C(16)-C(17)-H(17)	119.2
C(18)-C(17)-C(16)	121.7(4)
C(18)-C(17)-H(17)	119.2
C(13)-C(18)-C(22)	122.9(4)
C(17)-C(18)-C(13)	117.6(4)
C(17)-C(18)-C(22)	119.3(4)
C(14)-C(19)-H(19)	108.5
C(14)-C(19)-C(20)	112.6(4)
C(14)-C(19)-C(21)	108.9(4)
C(20)-C(19)-H(19)	108.5
C(21)-C(19)-H(19)	108.5
C(21)-C(19)-C(20)	109.9(4)
C(19)-C(20)-H(20A)	109.5
C(19)-C(20)-H(20B)	109.5
C(19)-C(20)-H(20C)	109.5
H(20A)-C(20)-H(20B)	109.5
H(20A)-C(20)-H(20C)	109.5
H(20B)-C(20)-H(20C)	109.5
C(19)-C(21)-H(21A)	109.5
C(19)-C(21)-H(21B)	109.5
C(19)-C(21)-H(21C)	109.5
H(21A)-C(21)-H(21B)	109.5
H(21A)-C(21)-H(21C)	109.5
H(21B)-C(21)-H(21C)	109.5
C(18)-C(22)-H(22)	108.3
C(18)-C(22)-C(23)	112.0(4)
C(18)-C(22)-C(24)	109.8(4)
C(23)-C(22)-H(22)	108.3
C(24)-C(22)-H(22)	108.3
C(24)-C(22)-C(23)	110.3(4)
C(22)-C(23)-H(23A)	109.5
C(22)-C(23)-H(23B)	109.5
C(22)-C(23)-H(23C)	109.5
H(23A)-C(23)-H(23B)	109.5
H(23A)-C(23)-H(23C)	109.5
H(23B)-C(23)-H(23C)	109.5
C(22)-C(24)-H(24A)	109.5
C(22)-C(24)-H(24B)	109.5
C(22)-C(24)-H(24C)	109.5
H(24A)-C(24)-H(24B)	109.5
H(24A)-C(24)-H(24C)	109.5
H(24B)-C(24)-H(24C)	109.5

Symmetry transformations used to generate equivalent atoms:

#1 -x+1,y,-z+1/2

Table S5. Crystal data and structure refinement for complex **8 (BF_4)**.

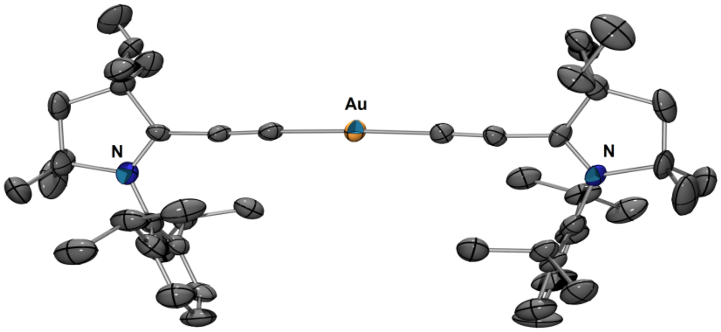

Identification code	**8 (BF_4)** (CCDC 1418633)	
Empirical formula	C50 H74 Au B Cl4 F4 N2	
Molecular formula	C48 H70 Au N2, B F4, 2(C H2 Cl2)	
Temperature	100 K	
Wavelength	0.71073 Å	
Crystal system	Monoclinic	
Space group	P 1 21/c 1	
Unit cell dimensions	a = 15.393(4) Å	a= 90°.
	b = 14.569(4) Å	b= 106.993(5)°.
	c = 24.605(6) Å	g = 90°.
Volume	5277(2) $Å^3$	
Density (calculated)	1.421 Mg/m^3	
Absorption coefficient	3.039 mm^{-1}	
F(000)	2304	
Crystal size	0.147 x 0.135 x 0.054 mm^3	
Crystal color, habit	Yellow Plate	
Theta range for data collection	1.731 to 26.448°.	
Index ranges	-13<=h<=19, -17<=k<=18, -30<=l<=27	
Reflections collected	28966	
Independent reflections	10760 [R(int) = 0.0732]	
Completeness to theta = 25.000°	99.6 %	
Absorption correction	Semi-empirical from equivalents	
Max. and min. transmission	0.0627 and 0.0315	
Refinement method	Full-matrix least-squares on F^2	
Data / restraints / parameters	10760 / 100 / 617	
Goodness-of-fit on F^2	1.007	
Final R indices [I>2sigma(I)]	R1 = 0.0499, wR2 = 0.0979	
R indices (all data)	R1 = 0.1203, wR2 = 0.1179	
Extinction coefficient	n/a	
Largest diff. peak and hole	1.426 and -0.952 e.$Å^{-3}$	

Table S6. Bond lengths [Å] and angles [°] for **8 (BF_4)**.

Au(1)-C(1)	1.988(6)	C(8)-H(8A)	0.9800	C(14')-C(15')	1.380(11)
Au(1)-C(1')	1.982(6)	C(8)-H(8B)	0.9800	C(14')-C(19')	1.498(10)
N(1)-C(3)	1.308(8)	C(8)-H(8C)	0.9800	C(15)-H(15)	0.9500
N(1)-C(6)	1.528(8)	C(8')-H(8'A)	0.9800	C(15)-C(16)	1.363(11)
N(1)-C(13)	1.448(9)	C(8')-H(8'B)	0.9800	C(15')-H(15')	0.9500
N(1')-C(3')	1.298(8)	C(8')-H(8'C)	0.9800	C(15')-C(16')	1.360(11)
N(1')-C(6')	1.507(8)	C(9)-H(9A)	0.9900	C(16)-H(16)	0.9500
N(1')-C(13')	1.463(9)	C(9)-H(9B)	0.9900	C(16)-C(17)	1.384(11)
C(1)-C(2)	1.209(8)	C(9)-C(10)	1.494(10)	C(16')-H(16')	0.9500
C(1')-C(2')	1.176(8)	C(9')-H(9'A)	0.9900	C(16')-C(17')	1.373(13)
C(2)-C(3)	1.409(8)	C(9')-H(9'B)	0.9900	C(17)-H(17)	0.9500
C(2')-C(3')	1.427(8)	C(9')-C(10')	1.528(10)	C(17)-C(18)	1.384(10)
C(3)-C(4)	1.522(10)	C(10)-H(10A)	0.9800	C(17')-H(17')	0.9500
C(3')-C(4')	1.513(10)	C(10)-H(10B)	0.9800	C(17')-C(18')	1.359(11)
C(4)-C(5)	1.530(10)	C(10)-H(10C)	0.9800	C(18)-C(22)	1.536(10)
C(4)-C(9)	1.527(11)	C(10')-H(10D)	0.9800	C(18')-C(22')	1.513(10)
C(4)-C(11)	1.529(10)	C(10')-H(10E)	0.9800	C(19)-H(19)	1.0000
C(4')-C(5')	1.543(10)	C(10')-H(10F)	0.9800	C(19)-C(20)	1.544(11)
C(4')-C(9')	1.550(10)	C(11)-H(11A)	0.9900	C(19)-C(21)	1.496(10)
C(4')-C(11')	1.524(10)	C(11)-H(11B)	0.9900	C(19')-H(19')	1.0000
C(5)-H(5A)	0.9900	C(11)-C(12)	1.531(11)	C(19')-C(20')	1.541(9)
C(5)-H(5B)	0.9900	C(11')-H(11C)	0.9900	C(19')-C(21')	1.523(9)
C(5)-C(6)	1.531(11)	C(11')-H(11D)	0.9900	C(20)-H(20A)	0.9800
C(5')-H(5'A)	0.9900	C(11')-C(12')	1.523(10)	C(20)-H(20B)	0.9800
C(5')-H(5'B)	0.9900	C(12)-H(12A)	0.9800	C(20)-H(20C)	0.9800
C(5')-C(6')	1.519(12)	C(12)-H(12B)	0.9800	C(20')-H(20D)	0.9800
C(6)-C(7)	1.529(11)	C(12)-H(12C)	0.9800	C(20')-H(20E)	0.9800
C(6)-C(8)	1.508(10)	C(12')-H(12D)	0.9800	C(20')-H(20F)	0.9800
C(6')-C(7')	1.538(10)	C(12')-H(12E)	0.9800	C(21)-H(21A)	0.9800
C(6')-C(8')	1.515(12)	C(12')-H(12F)	0.9800	C(21)-H(21B)	0.9800
C(7)-H(7A)	0.9800	C(13)-C(14)	1.410(10)	C(21)-H(21C)	0.9800
C(7)-H(7B)	0.9800	C(13)-C(18)	1.392(10)	C(21)-H(21D)	0.9800
C(7)-H(7C)	0.9800	C(13')-C(14')	1.392(10)	C(21)-H(21E)	0.9800
C(7')-H(7'A)	0.9800	C(13')-C(18')	1.409(10)	C(21)-H(21F)	0.9800
C(7')-H(7'B)	0.9800	C(14)-C(15)	1.399(11)	C(22)-H(22)	1.0000
C(7')-H(7'C)	0.9800	C(14)-C(19)	1.512(10)	C(22)-C(23)	1.552(9)

C(22)-C(24)	1.538(10)	C(2S)-H(2SC)	0.9900	C(4')-C(5')-H(5'A)	110.1
C(22')-H(22')	1.0000	C(2S)-H(2SD)	0.9900	C(4')-C(5')-H(5'B)	110.1
C(22')-C(23')	1.538(11)			H(5'A)-C(5')-H(5'B)	108.4
C(22')-C(24')	1.536(10)	C(1)-Au(1)-C(1')	177.4(3)	C(6')-C(5')-C(4')	108.0(6)
C(23)-H(23A)	0.9800	C(3)-N(1)-C(6)	112.9(6)	C(6')-C(5')-H(5'A)	110.1
C(23)-H(23B)	0.9800	C(3)-N(1)-C(13)	123.2(6)	C(6')-C(5')-H(5'B)	110.1
C(23)-H(23C)	0.9800	C(13)-N(1)-C(6)	123.9(6)	N(1)-C(6)-C(5)	101.5(6)
C(23')-H(23D)	0.9800	C(3')-N(1')-C(6')	112.8(6)	N(1)-C(6)-C(7)	110.8(6)
C(23')-H(23E)	0.9800	C(3')-N(1')-C(13')	124.1(7)	C(7)-C(6)-C(5)	111.5(7)
C(23')-H(23F)	0.9800	C(13')-N(1')-C(6')	123.0(8)	C(8)-C(6)-N(1)	110.1(7)
C(24)-H(24A)	0.9800	C(2)-C(1)-Au(1)	176.5(8)	C(8)-C(6)-C(5)	114.9(7)
C(24)-H(24B)	0.9800	C(2')-C(1')-Au(1)	176.4(8)	C(8)-C(6)-C(7)	108.0(7)
C(24)-H(24C)	0.9800	C(1)-C(2)-C(3)	173.9(7)	N(1')-C(6')-C(5')	102.4(5)
C(24')-H(24D)	0.9800	C(1')-C(2')-C(3')	175.4(7)	N(1')-C(6')-C(7')	109.6(7)
C(24')-H(24E)	0.9800	N(1)-C(3)-C(2)	122.9(6)	N(1')-C(6')-C(8')	111.5(7)
C(24')-H(24F)	0.9800	N(1)-C(3)-C(4)	113.1(5)	C(5')-C(6')-C(7')	111.9(8)
F(1)-B(1)	1.394(11)	C(2)-C(3)-C(4)	124.0(8)	C(8')-C(6')-C(5')	113.0(7)
F(2)-B(1)	1.369(17)	N(1')-C(3')-C(2')	123.4(7)	C(8')-C(6')-C(7')	108.3(7)
F(3)-B(1)	1.321(16)	N(1')-C(3')-C(4')	113.2(6)	C(6)-C(7)-H(7A)	109.5
F(4)-B(1)	1.412(13)	C(2')-C(3')-C(4')	123.4(6)	C(6)-C(7)-H(7B)	109.5
F(1B)-B(1)	1.40(2)	C(3)-C(4)-C(5)	101.4(6)	C(6)-C(7)-H(7C)	109.5
F(2B)-B(1)	1.39(4)	C(3)-C(4)-C(9)	109.4(6)	H(7A)-C(7)-H(7B)	109.5
F(3B)-B(1)	1.44(2)	C(3)-C(4)-C(11)	105.5(6)	H(7A)-C(7)-H(7C)	109.5
F(4B)-B(1)	1.41(3)	C(9)-C(4)-C(5)	114.1(7)	H(7B)-C(7)-H(7C)	109.5
Cl(1S)-C(1S)	1.794(5)	C(9)-C(4)-C(11)	111.7(6)	C(6')-C(7')-H(7'A)	109.5
Cl(2S)-C(1S)	1.797(5)	C(11)-C(4)-C(5)	113.8(6)	C(6')-C(7')-H(7'B)	109.5
Cl(1B)-C(1SB)	1.798(5)	C(3')-C(4')-C(5')	101.3(6)	C(6')-C(7')-H(7'C)	109.5
Cl(2B)-C(1SB)	1.803(5)	C(3')-C(4')-C(9')	105.2(6)	H(7'A)-C(7')-H(7'B)	109.5
C(1S)-H(1SA)	0.9900	C(3')-C(4')-C(11')	111.1(5)	H(7'A)-C(7')-H(7'C)	109.5
C(1S)-H(1SB)	0.9900	C(5')-C(4')-C(9')	113.8(6)	H(7'B)-C(7')-H(7'C)	109.5
C(1SB)-H(1SC)	0.9900	C(11')-C(4')-C(5')	114.0(7)	C(6)-C(8)-H(8A)	109.5
C(1SB)-H(1SD)	0.9900	C(11')-C(4')-C(9')	110.7(7)	C(6)-C(8)-H(8B)	109.5
Cl(3S)-C(2S)	1.674(12)	C(4)-C(5)-H(5A)	109.9	C(6)-C(8)-H(8C)	109.5
Cl(4S)-C(2S)	1.630(13)	C(4)-C(5)-H(5B)	109.9	H(8A)-C(8)-H(8B)	109.5
Cl(3B)-C(2S)	1.778(19)	C(4)-C(5)-C(6)	109.0(6)	H(8A)-C(8)-H(8C)	109.5
Cl(4B)-C(2S)	1.73(2)	H(5A)-C(5)-H(5B)	108.3	H(8B)-C(8)-H(8C)	109.5
C(2S)-H(2SA)	0.9900	C(6)-C(5)-H(5A)	109.9	C(6')-C(8')-H(8'A)	109.5
C(2S)-H(2SB)	0.9900	C(6)-C(5)-H(5B)	109.9	C(6')-C(8')-H(8'B)	109.5

C(6')-C(8')-H(8'C)	109.5	C(12')-C(11')-H(11C)	108.6
C(16)-C(17)-H(17)	119.8		
H(8'A)-C(8')-H(8'B)	109.5	C(12')-C(11')-H(11D)	108.6
C(16)-C(17)-C(18)	120.5(8)		
H(8'A)-C(8')-H(8'C)	109.5	C(11)-C(12)-H(12A)	109.5
C(18)-C(17)-H(17)	119.8		
H(8'B)-C(8')-H(8'C)	109.5	C(11)-C(12)-H(12B)	109.5
C(16')-C(17')-H(17')	118.5		
C(4)-C(9)-H(9A)	108.2	C(11)-C(12)-H(12C)	109.5
C(18')-C(17')-C(16')	123.1(8)		
C(4)-C(9)-H(9B)	108.2	H(12A)-C(12)-H(12B)	109.5
C(18')-C(17')-H(17')	118.5		
H(9A)-C(9)-H(9B)	107.4	H(12A)-C(12)-H(12C)	109.5
C(13)-C(18)-C(22)	122.8(7)		
C(10)-C(9)-C(4)	116.3(7)	H(12B)-C(12)-H(12C)	109.5
C(17)-C(18)-C(13)	117.9(7)		
C(10)-C(9)-H(9A)	108.2	C(11')-C(12')-H(12D)	109.5
C(17)-C(18)-C(22)	119.1(7)		
C(10)-C(9)-H(9B)	108.2	C(11')-C(12')-H(12E)	109.5
C(13')-C(18')-C(22')	124.2(7)		
C(4')-C(9')-H(9'A)	108.8	C(11')-C(12')-H(12F)	109.5
C(17')-C(18')-C(13')	115.5(8)		
C(4')-C(9')-H(9'B)	108.8	H(12D)-C(12')-H(12E)	109.5
C(17')-C(18')-C(22')	119.9(8)		
H(9'A)-C(9')-H(9'B)	107.7	H(12D)-C(12')-H(12F)	109.5
C(14)-C(19)-H(19)	107.8		
C(10')-C(9')-C(4')	113.9(7)	H(12E)-C(12')-H(12F)	109.5
C(14)-C(19)-C(20)	111.9(7)		
C(10')-C(9')-H(9'A)	108.8	C(14)-C(13)-N(1)	117.0(7)
C(20)-C(19)-H(19)	107.8		
C(10')-C(9')-H(9'B)	108.8	C(18)-C(13)-N(1)	119.4(7)
C(21)-C(19)-C(14)	110.2(7)		
C(9)-C(10)-H(10A)	109.5	C(18)-C(13)-C(14)	122.0(7)
C(21)-C(19)-H(19)	107.8		
C(9)-C(10)-H(10B)	109.5	C(14')-C(13')-N(1')	118.0(6)
C(21)-C(19)-C(20)	111.2(7)		
C(9)-C(10)-H(10C)	109.5	C(14')-C(13')-C(18')	124.0(7)
C(14')-C(19')-H(19')	107.8		
H(10A)-C(10)-H(10B)	109.5	C(18')-C(13')-N(1')	117.8(7)
C(14')-C(19')-C(20')	113.4(7)		
H(10A)-C(10)-H(10C)	109.5	C(13)-C(14)-C(19)	124.0(8)
C(14')-C(19')-C(21')	110.3(6)		
H(10B)-C(10)-H(10C)	109.5	C(15)-C(14)-C(13)	116.5(7)
C(20')-C(19')-H(19')	107.8		
C(9')-C(10')-H(10D)	109.5	C(15)-C(14)-C(19)	119.2(7)
C(21')-C(19')-H(19')	107.8		
C(9')-C(10')-H(10E)	109.5	C(13')-C(14')-C(19')	124.4(7)
C(21')-C(19')-C(20')	109.4(6)		
C(9')-C(10')-H(10F)	109.5	C(15')-C(14')-C(13')	115.2(7)
C(19)-C(20)-H(20A)	109.5		
H(10D)-C(10')-H(10E)	109.5	C(15')-C(14')-C(19')	120.2(7)
C(19)-C(20)-H(20B)	109.5		
H(10D)-C(10')-H(10F)	109.5	C(14)-C(15)-H(15)	119.4
C(19)-C(20)-H(20C)	109.5		
H(10E)-C(10')-H(10F)	109.5	C(16)-C(15)-C(14)	121.2(8)
H(20A)-C(20)-H(20B)	109.5		
C(4)-C(11)-H(11A)	108.9	C(16)-C(15)-H(15)	119.4
H(20A)-C(20)-H(20C)	109.5		
C(4)-C(11)-H(11B)	108.9	C(14')-C(15')-H(15')	118.5
H(20B)-C(20)-H(20C)	109.5		
C(4)-C(11)-C(12)	113.3(7)	C(16')-C(15')-C(14')	123.0(8)
C(19')-C(20')-H(20D)	109.5		
H(11A)-C(11)-H(11B)	107.7	C(16')-C(15')-H(15')	118.5
C(19')-C(20')-H(20E)	109.5		
C(12)-C(11)-H(11A)	108.9	C(15)-C(16)-H(16)	119.5
C(19')-C(20')-H(20F)	109.5		
C(12)-C(11)-H(11B)	108.9	C(15)-C(16)-C(17)	121.0(9)
H(20D)-C(20')-H(20E)	109.5		
C(4')-C(11')-H(11C)	108.6	C(17)-C(16)-H(16)	119.5
H(20D)-C(20')-H(20F)	109.5		
C(4')-C(11')-H(11D)	108.6	C(15')-C(16')-H(16')	120.6
H(20E)-C(20')-H(20F)	109.5		
H(11C)-C(11')-H(11D)	107.6	C(15')-C(16')-C(17')	118.8(9)
C(19)-C(21)-H(21A)	109.5		
C(12')-C(11')-C(4')	114.5(7)	C(17')-C(16')-H(16')	120.6
C(19)-C(21)-H(21B)	109.5		

C(19)-C(21)-H(21C)	109.5	C(22')-C(23')-H(23D)	109.5	F(2B)-B(1)-F(4B)	116(2)
H(21A)-C(21)-H(21B)	109.5	C(22')-C(23')-H(23E)	109.5	F(4B)-B(1)-F(3B)	108.0(16)
H(21A)-C(21)-H(21C)	109.5	C(22')-C(23')-H(23F)	109.5	Cl(1S)-C(1S)-Cl(2S)	109.9(7)
H(21B)-C(21)-H(21C)	109.5	H(23D)-C(23')-H(23E)	109.5	Cl(1S)-C(1S)-H(1SA)	109.7
C(19')-C(21')-H(21D)	109.5	H(23D)-C(23')-H(23F)	109.5	Cl(1S)-C(1S)-H(1SB)	109.7
C(19')-C(21')-H(21E)	109.5	H(23E)-C(23')-H(23F)	109.5	Cl(2S)-C(1S)-H(1SA)	109.7
C(19')-C(21')-H(21F)	109.5	C(22)-C(24)-H(24A)	109.5	Cl(2S)-C(1S)-H(1SB)	109.7
H(21D)-C(21')-H(21E)	109.5	C(22)-C(24)-H(24B)	109.5	H(1SA)-C(1S)-H(1SB)	108.2
H(21D)-C(21')-H(21F)	109.5	C(22)-C(24)-H(24C)	109.5	Cl(1B)-C(1SB)-Cl(2B)	106.5(13)
H(21E)-C(21')-H(21F)	109.5	H(24A)-C(24)-H(24B)	109.5	Cl(1B)-C(1SB)-H(1SC)	110.4
C(18)-C(22)-H(22)	108.7	H(24A)-C(24)-H(24C)	109.5	Cl(1B)-C(1SB)-H(1SD)	110.4
C(18)-C(22)-C(23)	111.7(7)	H(24B)-C(24)-H(24C)	109.5	Cl(2B)-C(1SB)-H(1SC)	110.4
C(18)-C(22)-C(24)	110.0(5)	C(22')-C(24')-H(24D)	109.5	Cl(2B)-C(1SB)-H(1SD)	110.4
C(23)-C(22)-H(22)	108.7	C(22')-C(24')-H(24E)	109.5	H(1SC)-C(1SB)-H(1SD)	108.6
C(24)-C(22)-H(22)	108.7	C(22')-C(24')-H(24F)	109.5	Cl(3S)-C(2S)-H(2SA)	106.3
C(24)-C(22)-C(23)	108.9(7)	H(24D)-C(24')-H(24E)	109.5	Cl(3S)-C(2S)-H(2SB)	106.3
C(18')-C(22')-H(22')	107.3	H(24D)-C(24')-H(24F)	109.5	Cl(4S)-C(2S)-Cl(3S)	124.0(9)
C(18')-C(22')-C(23')	112.9(7)	H(24E)-C(24')-H(24F)	109.5	Cl(4S)-C(2S)-H(2SA)	106.3
C(18')-C(22')-C(24')	109.4(7)	F(1)-B(1)-F(4)	106.8(8)	Cl(4S)-C(2S)-H(2SB)	106.3
C(23')-C(22')-H(22')	107.3	F(2)-B(1)-F(1)	106.6(10)	Cl(3B)-C(2S)-H(2SC)	111.9
C(24')-C(22')-H(22')	107.3	F(2)-B(1)-F(4)	105.6(12)	Cl(3B)-C(2S)-H(2SD)	111.9
C(24')-C(22')-C(23')	112.2(7)	F(3)-B(1)-F(1)	110.9(9)	Cl(4B)-C(2S)-Cl(3B)	99.6(10)
C(22)-C(23)-H(23A)	109.5	F(3)-B(1)-F(2)	115.8(10)	Cl(4B)-C(2S)-H(2SC)	111.9
C(22)-C(23)-H(23B)	109.5	F(3)-B(1)-F(4)	110.6(10)	Cl(4B)-C(2S)-H(2SD)	111.9
C(22)-C(23)-H(23C)	109.5	F(1B)-B(1)-F(3B)	100.8(14)	H(2SA)-C(2S)-H(2SB)	106.4
H(23A)-C(23)-H(23B)	109.5	F(1B)-B(1)-F(4B)	111.4(13)	H(2SC)-C(2S)-H(2SD)	109.6
H(23A)-C(23)-H(23C)	109.5	F(2B)-B(1)-F(1B)	110(3)		
H(23B)-C(23)-H(23C)	109.5	F(2B)-B(1)-F(3B)	109.7(18)		

----

6. Computational details

Quantum chemical calculations of **6** and **7** were carried out using the M05-2X [S1] (global hybrid functional with 52% HF exchange) with Ahlrichs basis set def2-SVP [S2] method. The Au relativistic effect was accounted for by the Stuttgart-Dresden ECP [S3]. The method is referred to as M05-2X/SDD/def2-SVP. The g factor of **7** at its optimized geometry was calculated using the B3LYP[23] (hybrid functional with 20% HF exchange) functional with the TZVP basis set[S4] and the zero-order regular approximation (ZORA)[S5] for the relativistic effect in Au. The method is referred to as ZORA/ B3LYP/TZVP. Additionally to M05-2X/SDD/def2-SVP the optimizations of **6** and **7** were carried out using ZORA/B3LYP-D3BJ[S6]/TZVP and ZORA/M06L/TZVP (M06L[S7] - local functional with 0% HF exchange) methods for comparison. All methods yield similar results (Table S7, S8). NBO analysis was done using NBO 6.0 software [S8]. The calculations were performed using the Gaussian 09 [S9] and ORCA 3.0.1 [S10] programs.

[S1] Y. Zhao, N. E. Schultz, D. G. Truhlar, *J. Chem. Theory and Comput.*, 2 (2006) 364-82.

[S2] F. Weigend and R. Ahlrichs, *Phys. Chem. Chem. Phys.*, **2005**, 7, 3297-3305.

[S3] P. Schwerdtfeger, M. Dolg, W. H. E. Schwarz, G. A. Bowmaker, P. D. W. Boyd, *J. Chem. Phys.*, 91 (1989) 1762-74.

[S4] (a) A. Schäfer, C. Huber and R. Ahlrichs, *J. Chem. Phys.*, 1994, **100**, 5829–5835; (b) A. Schäfer, H. Horn and R. Ahlrichs, *J. Chem. Phys.*, 1992, **97**, 2571–2577.

[S5] (a) D. A. Pantazis, X. Y. Chen, C. R. Landis and F. Neese, *J. Chem. Theory Comput.*, 2008, **4**, 908-919. (b) D. A. Pantazis and F. Neese, *J. Chem. Theory Comput*., **2011**, 7, 677-684.

[S6] S.Grimme, S.Ehrlich, L.Goerigk, *J Comput Chem*, **2011**, 32, 1456–1465

[S7] Y. Zhao and D. G. Truhlar, *J. Chem. Phys.*, **2006**, 125, 194101: 1-18.

[S8] NBO 6.0. E. D. Glendening, J, K. Badenhoop, A. E. Reed, J. E. Carpenter, J. A. Bohmann, C. M. Morales, C. R. Landis, and F. Weinhold, Theoretical Chemistry Institute, University of Wisconsin, Madison (2013).

[S9] Gaussian 09, Revision D.01, M. J. Frisch, G. W. Trucks, H. B. Schlegel, G. E. Scuseria, M. A. Robb, J. R. Cheeseman, G. Scalmani, V. Barone, B. Mennucci, G. A. Petersson, H. Nakatsuji, M. Caricato, X. Li, H. P. Hratchian, A. F. Izmaylov, J. Bloino, G. Zheng, J. L. Sonnenberg, M. Hada, M. Ehara, K. Toyota, R. Fukuda, J. Hasegawa, M. Ishida, T. Nakajima, Y. Honda, O. Kitao, H. Nakai, T. Vreven, J. A. Montgomery, Jr., J. E. Peralta, F. Ogliaro, M. Bearpark, J. J. Heyd, E. Brothers, K. N. Kudin, V. N. Staroverov, R. Kobayashi, J. Normand, K. Raghavachari, A. Rendell, J. C. Burant, S. S. Iyengar, J. Tomasi, M. Cossi, N. Rega, J. M. Millam, M. Klene, J. E. Knox, J. B. Cross, V. Bakken, C. Adamo, J. Jaramillo, R. Gomperts, R. E. Stratmann, O. Yazyev, A. J. Austin, R. Cammi, C. Pomelli, J. W. Ochterski, R. L. Martin, K. Morokuma, V. G. Zakrzewski, G. A. Voth, P. Salvador, J. J. Dannenberg, S. Dapprich, A. D. Daniels, Ö. Farkas, J. B. Foresman, J. V. Ortiz, J. Cioslowski, and D. J. Fox, Gaussian, Inc., Wallingford CT, 2009.

[S10] F. Neese, *WIREs Comput. Mol. Sci.*, 2012, **2**, 73–78.

Table S7. Selected parameters of the optimized geometries (at M05-2X/SDD/def2-SVP) of cation **6** and neutral **7**.

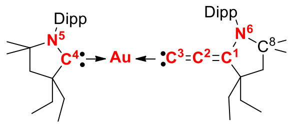

	r(C⁴-Au) (Å)	r(Au-C³) (Å)	r(C³-C²) (Å)	r(C²-C¹) (Å)	r(C⁴-N⁵) (Å)	r(C¹-N⁶) (Å)	C⁴- Au-C³ (deg)	Au- C³-C² (deg)	C³- C²-C¹ (deg)	N⁵- C⁴-C¹-N⁶ (deg)	Σα(N⁶) (deg)
**6**	2.040	2.002	1.229	1.399	1.298	1.304	177.34	172.48	178.82	42.00	359.79
**7**	2.027	1.988	1.243	1.380	1.307	1.386	176.43	170.94	179.59	23.11	359.95

Table S8. Mulliken spin density distribution (%) in **7** (with hydrogens summed up into the heavy atoms).

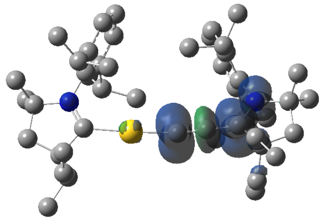

Au	C⁴	C³	C²	C¹	N⁵	N⁶
1.8	1.7	44.5	-23.0	55.9	1.4	16.4

Table S9. NPA charges in **6** and **7**.

	Au	carbene	allenylidene
**6**	0.43	-0.45	1.02
**7**	0.41	-0.55	0.14

Table S10. Comparison of the selected parameters of the optimized geometries of cation **6** at ZORA-M06L /TZVP, ZORA-B3LYP-D3BJ/TZVP and M05-2X/SDD/def2-SVP levels.

Method	r(C⁴-Au) (Å)	r(Au-C³) (Å)	r(C³-C²) (Å)	r(C²-C¹) (Å)	r(C⁴-N⁵) (Å)	r(C¹-N⁶) (Å)	C⁴- Au-C³ (deg)	Au- C³-C² (deg)	C³- C²-C¹ (deg)	N⁵- C⁴-C¹-N⁶ (deg)
ZORA-M06L /TZVP	2.047	1.982	1.230	1.382	1.303	1.314	176.79	172.48	177.77	37.16
ZORA-B3LYP-D3BJ/TZVP	2.038	1.983	1.222	1.382	1.299	1.310	176.84	171.12	177.70	30.02
M05-2X/SDD/def2-SVP	2.040	2.002	1.229	1.399	1.298	1.304	177.34	172.48	178.82	42.00

Table S11. Comparison of the selected parameters of the optimized geometries of neutral **7** at ZORA-M06L /TZVP, ZORA-B3LYP-D3BJ/TZVP and M05-2X/SDD/def2-SVP levels.

Method	r(C⁴-Au) (Å)	r(Au-C³) (Å)	r(C³-C²) (Å)	r(C²-C¹) (Å)	r(C⁴-N⁵) (Å)	r(C¹-N⁶) (Å)	C⁴- Au-C³ (deg)	Au- C³-C² (deg)	C³- C²-C¹ (deg)	N⁵- C⁴-C¹-N⁶ (deg)
ZORA-M06L /TZVP	2.011	1.963	1.244	1.362	1.328	1.374	176.49	171.06	178.77	24.21
ZORA-B3LYP-D3BJ/TZVP	2.011	1.969	1.237	1.362	1.318	1.382	176.70	171.01	178.06	21.12
M05-2X/SDD/def2-SVP	2.027	1.988	1.243	1.380	1.307	1.386	176.43	170.94	179.59	23.11

Table S12. Cartesian coordinates for the optimized structure of **6** at M05-2X/SDD/def2-SVP

Total electronic energy: -2038.33949 E_h

N	4.17403000	0.05700100	0.08822000
C	2.79248100	2.05529300	1.81334300
C	3.49519500	-0.99653900	0.44823300
C	5.77534700	-1.48855600	0.91099100
C	1.28240800	1.97711100	2.09359900
C	5.64217700	-0.02634300	0.42977000
C	4.13086900	-3.41537800	0.41662300
C	2.59971800	-0.42047100	-3.41479700
C	3.92922900	-2.11417800	2.59389300
C	4.34925600	-2.05274000	1.10316500
C	3.89756500	0.16293100	-2.83283600
C	4.86675600	0.54049000	-3.95741000
C	3.41771200	3.17879100	2.64905100
C	6.51093800	0.26563200	-0.78890900
C	5.96753500	0.98412500	1.52789500
C	4.23132900	-3.39091000	-1.10690500
C	4.64128000	-3.19931200	3.39765800
Au	-1.09206300	-1.08311800	-0.09106700
N	-3.76912700	0.19821100	-0.06747500
C	-2.37986700	2.13873600	-1.85113700
C	-3.10301900	-0.87561600	-0.36507700
C	-5.42594300	-1.30044800	-0.84778700
C	-0.87853200	1.99502100	-2.14713900
C	-5.24740300	0.16402700	-0.39954500
C	-3.85057800	-3.25683600	-0.26853200
C	-2.22733400	-0.20545600	3.46024100
C	-3.57625600	-2.02662300	-2.47445800
C	-4.00971600	-1.90532100	-0.99162500
C	-3.50321700	0.39879000	2.85480100
C	-4.46233600	0.84363900	3.96376400
C	-2.97070100	3.26657000	-2.70529700
C	-6.09928600	0.50947300	0.81791800
C	-5.55248600	1.16201100	-1.51447900
C	-3.96722000	-3.18113300	1.25224800
C	-4.31153800	-3.11050000	-3.25960000
C	2.11094500	-1.10923400	0.27750200
C	0.89185700	-1.19830700	0.15062900
H	3.23604000	1.10370800	2.12814400
H	6.32341900	-2.07637900	0.16620500
H	6.34587500	-1.53161200	1.84514400
H	0.79950200	1.19861300	1.49128400
H	1.10779600	1.75842400	3.15563400

H	0.79718400	2.93387500	1.86175000
H	3.14092800	-3.79108900	0.71256100
H	4.87480900	-4.11057600	0.82761600
H	2.82339800	-1.30863400	-4.02057900
H	2.10436500	0.31650500	-4.06133500
H	1.89893900	-0.70321500	-2.61938100
H	2.84295900	-2.27533200	2.63677300
H	4.12122700	-1.13116000	3.04892100
H	4.36224800	-0.62437200	-2.23071100
H	5.14881200	-0.35704500	-4.52253100
H	4.40165800	1.23764300	-4.66578100
H	5.78047500	1.00881100	-3.57264900
H	4.48824300	3.30158200	2.45139600
H	2.92619600	4.13831000	2.44438900
H	3.28621000	2.96516200	3.71748900
H	6.32207100	1.27374000	-1.17792000
H	6.35494100	-0.46634000	-1.58818500
H	7.56124900	0.21006900	-0.47735700
H	5.76304600	2.00553200	1.18642100
H	7.03621600	0.90857800	1.76214700
H	5.40103200	0.79008000	2.44536300
H	4.12750200	-4.40576600	-1.50777900
H	3.43519700	-2.77924200	-1.55175600
H	5.19984400	-3.00389700	-1.45127200
H	5.73309600	-3.12984600	3.30626200
H	4.33547700	-4.20245600	3.08038800
H	4.39038400	-3.09858100	4.45996900
H	-2.86133300	1.19843000	-2.13875900
H	-5.97869700	-1.34732800	-1.79280400
H	-6.01066000	-1.85151300	-0.10238600
H	-0.72394600	1.75941600	-3.20873300
H	-0.34862600	2.93046300	-1.92579500
H	-0.42010900	1.20027700	-1.54379500
H	-4.61132100	-3.94271000	-0.66473700
H	-2.86974900	-3.67683900	-0.53840800
H	-1.54005000	-0.54990500	2.67533600
H	-2.47944200	-1.05860000	4.10394900
H	-1.69868100	0.53983600	4.06987500
H	-3.72866200	-1.05149500	-2.96134500
H	-2.49416700	-2.22238900	-2.50297700
H	-3.99032400	-0.39275900	2.27787100
H	-5.35882900	1.32832100	3.55951000
H	-4.77593800	-0.02610400	4.55532700
H	-3.97765400	1.54765300	4.65204200

H	-2.86082400	3.02365800	-3.77002900
H	-4.03355000	3.43260900	-2.49810500
H	-2.44203100	4.21201100	-2.52875600
H	-7.15283000	0.49193200	0.51261900
H	-5.86875900	1.51453300	1.19247800
H	-5.97036900	-0.21681800	1.62734700
H	-6.62500800	1.11382700	-1.73863800
H	-5.00166600	0.93309800	-2.43343500
H	-5.31629900	2.18433200	-1.19635700
H	-4.92571100	-2.74883300	1.57000400
H	-3.90071700	-4.18331000	1.69183700
H	-3.15824400	-2.57529900	1.68272100
H	-4.03918600	-3.05437300	-4.32010300
H	-5.40154400	-2.99948100	-3.18995500
H	-4.04899800	-4.11315000	-2.90376000
C	3.55804900	1.32815900	-1.91725200
C	3.09466000	2.52117600	-2.47728500
C	2.63633200	3.56045500	-1.67542500
C	2.57515900	3.39874700	-0.29657900
C	3.02484900	2.22653600	0.31913000
C	3.57580600	1.23028400	-0.51180200
C	-2.58982100	2.34890200	-0.35820400
C	-2.10394200	3.52466200	0.22317300
C	-2.14611800	3.72290800	1.59788400
C	-2.62647200	2.71640300	2.42791000
C	-3.12715200	1.52253100	1.90167700
C	-3.15432300	1.38237100	0.49934700
H	-1.67455500	4.29414000	-0.41701100
H	-1.77561800	4.65291400	2.02716200
H	-2.60306900	2.85426600	3.50774000
H	3.08037400	2.63114100	-3.56039300
H	2.29347300	4.48851500	-2.13102200
H	2.15643500	4.19301300	0.31974900

Table S13. Cartesian coordinates for the optimized structure of **7** at M05-2X/SDD/def2-SVP

Total electronic energy: -2038.48289 E_h

N	-4.14607	0.10824	-0.06300
C	-2.84984	1.80867	-2.10862
C	-3.46181	-1.07591	-0.28580
C	-5.79750	-1.52827	-0.42735
C	-1.39043	1.54892	-2.50893
C	-5.59496	0.00274	-0.33178

C	-4.20352	-3.45283	0.15501
C	-2.42908	-0.03027	3.37574
C	-4.19417	-2.50423	-2.18428
C	-4.41834	-2.17902	-0.68779
C	-3.70664	0.57526	2.77749
C	-4.59744	1.15910	3.87814
C	-3.46448	2.87792	-3.01918
C	-6.43451	0.60477	0.79700
C	-5.98758	0.70995	-1.63518
C	-4.11586	-3.22769	1.66226
C	-5.03055	-3.65274	-2.74647
Au	1.12241	-1.16950	0.04077
N	3.73169	0.24008	0.04392
C	2.36494	1.81084	2.17302
C	3.12578	-0.90648	0.20496
C	5.50411	-1.26470	0.44663
C	0.90454	1.51351	2.54559
C	5.21999	0.24199	0.28617
C	3.97958	-3.20612	-0.29050
C	2.02198	0.25538	-3.44085
C	3.82367	-2.31906	2.07978
C	4.13288	-1.96185	0.60419
C	3.28039	0.85138	-2.79400
C	4.15663	1.52935	-3.85292
C	2.94605	2.85344	3.13597
C	5.98223	0.85395	-0.88583
C	5.54750	1.03875	1.54872
C	3.97439	-2.91661	-1.79007
C	4.67851	-3.44491	2.65709
C	-2.09092	-1.20199	-0.18375
C	-0.85648	-1.30967	-0.08514
H	-3.39577	0.87043	-2.24372
H	-6.20863	-1.89471	0.52118
H	-6.52273	-1.77542	-1.21265
H	-0.94516	0.77261	-1.87530
H	-1.34096	1.21948	-3.55655
H	-0.79016	2.46438	-2.40885
H	-3.26944	-3.92384	-0.18632
H	-5.01837	-4.15770	-0.06725
H	-2.68631	-0.81598	4.09984
H	-1.84435	0.73968	3.89903
H	-1.80185	-0.46708	2.58804
H	-3.12516	-2.72904	-2.31008
H	-4.38423	-1.59122	-2.76642

H	-4.24502	-0.23872	2.28285
H	-4.91660	0.36384	4.56525
H	-4.05593	1.90752	4.47232
H	-5.49214	1.63976	3.46367
H	-4.49359	3.12394	-2.73195
H	-2.87621	3.80504	-2.98821
H	-3.47081	2.52707	-4.05999
H	-6.20816	1.67163	0.92675
H	-6.25787	0.08849	1.74661
H	-7.49888	0.50706	0.54608
H	-5.45837	0.29256	-2.49861
H	-5.76147	1.78097	-1.56657
H	-7.06658	0.59695	-1.80475
H	-4.01125	-4.18691	2.18540
H	-3.24708	-2.60641	1.91013
H	-5.01467	-2.73501	2.05812
H	-6.10393	-3.50600	-2.56372
H	-4.74349	-4.61526	-2.30652
H	-4.88763	-3.73146	-3.83179
H	2.92702	0.87862	2.28766
H	6.15393	-1.44481	1.31089
H	6.02842	-1.64084	-0.43961
H	0.85099	1.12293	3.57091
H	0.29400	2.42470	2.49238
H	0.45826	0.77429	1.86823
H	4.79546	-3.90196	-0.04995
H	3.03687	-3.70327	-0.01789
H	1.39137	-0.24314	-2.69298
H	2.30426	-0.47625	-4.21031
H	1.42222	1.04170	-3.91858
H	3.94915	-1.41201	2.68991
H	2.76042	-2.58969	2.14553
H	3.84266	0.01789	-2.36303
H	5.03667	2.01195	-3.41091
H	4.49899	0.78696	-4.58595
H	3.59235	2.29423	-4.40224
H	2.92940	2.46234	4.16176
H	3.97838	3.12484	2.88641
H	2.34449	3.77165	3.12592
H	7.05283	0.83964	-0.64536
H	5.68088	1.89560	-1.05365
H	5.83426	0.28487	-1.80948
H	6.63430	1.02974	1.69853
H	5.07474	0.60885	2.43865

H	5.22353	2.08047	1.43980
H	4.89103	-2.40489	-2.11506
H	3.90244	-3.85164	-2.35880
H	3.11310	-2.29314	-2.06379
H	4.46525	-3.56570	3.72614
H	5.75281	-3.24179	2.55308
H	4.46564	-4.40283	2.16872
C	-3.34122	1.59105	1.70858
C	-2.75926	2.80293	2.09068
C	-2.28429	3.70066	1.14055
C	-2.34712	3.37197	-0.20823
C	-2.92763	2.17292	-0.63562
C	-3.48334	1.30714	0.33171
C	2.44810	2.24119	0.71638
C	1.82558	3.43644	0.34241
C	1.74807	3.82566	-0.98841
C	2.25532	2.99600	-1.98151
C	2.88396	1.78913	-1.66446
C	3.02011	1.45268	-0.30230
H	1.36878	4.05789	1.11106
H	1.25870	4.76154	-1.25561
H	2.13739	3.27583	-3.02740
H	-2.65970	3.03696	3.15047
H	-1.83984	4.64546	1.45401
H	-1.92257	4.05056	-0.94771

Table S14. Cartesian coordinates for the optimized structure of **6** at ZORA/B3LYP-D3BJ/TZVP

Total electronic energy: -21581.18919 E_h

N	3.62995000	6.14787800	4.02452000
C	5.24424600	7.83623300	2.19171500
C	4.25083500	5.01049600	3.83537800
C	1.96466500	4.62056800	3.29942700
C	6.74105300	7.55654500	1.98098300
C	2.17535100	6.12324200	3.58799400
C	3.43674900	2.64660800	4.06879900
C	4.92592600	5.91027400	7.65549300
C	3.84578800	3.70374700	1.78768600
C	3.35713000	3.94903600	3.24281300
C	3.73631800	6.55717300	6.92493000
C	2.73873500	7.14600600	7.92898400
C	4.76811500	8.93282200	1.22971400
C	1.26196700	6.63681500	4.69469500

C	1.99631100	6.99062200	2.34328900
C	3.24125300	2.81482200	5.57440000
C	3.06498300	2.63413100	1.02789300
Au	8.72835400	4.81932200	4.73335200
N	11.35996400	6.15316400	4.98762400
C	9.82158400	7.90973100	6.82013400
C	10.71058800	5.04042300	5.15389000
C	13.01648300	4.62607600	5.71102300
C	8.32681000	7.63695000	7.04646600
C	12.82537500	6.12666800	5.42027000
C	11.52010600	2.67736300	4.93760600
C	10.09792100	5.93006700	1.35064100
C	11.10631500	3.75372500	7.20387600
C	11.60841900	3.98600700	5.75138800
C	11.28612600	6.57060500	2.08527100
C	12.29267200	7.14940500	1.08418100
C	10.30878700	9.01575000	7.76497100
C	13.74088000	6.63089300	4.31109700
C	13.01771100	6.99622300	6.66091800
C	11.72466800	2.83573200	3.43203700
C	11.86208900	2.67637400	7.97886600
C	5.59083600	4.83118900	4.12051900
C	6.78676500	4.71104500	4.34334500
H	4.70948800	6.92051800	1.94605200
H	1.36595000	4.17730800	4.09287200
H	1.41896800	4.48319000	2.36852800
H	7.09958000	6.77989400	2.65313800
H	6.92088400	7.23372200	0.95342100
H	7.33616200	8.45204300	2.16189000
H	4.40871600	2.18405000	3.88099100
H	2.68177500	1.96368200	3.67537300
H	4.57340900	5.12172100	8.32371700
H	5.45844100	6.64876000	8.25776400
H	5.63530600	5.47647800	6.95113200
H	4.90196400	3.42784600	1.82858200
H	3.79455100	4.64830400	1.23875700
H	3.23004200	5.76247400	6.38135200
H	2.34168500	6.35560300	8.56880800
H	3.21393200	7.88167600	8.57987200

H	1.90070300	7.63478100	7.43121400
H	3.71555800	9.17682800	1.37204100
H	5.34195600	9.85127100	1.36430500
H	4.90571600	8.61034000	0.19579700
H	1.50561800	7.66349500	4.96710400
H	1.31208600	6.01288000	5.58407800
H	0.23455000	6.61966900	4.32914700
H	2.60482000	6.64317000	1.51047600
H	2.24371100	8.03023000	2.55236200
H	0.95015500	6.94904900	2.03852700
H	3.25519000	1.84117800	6.06546800
H	4.03684500	3.41507900	6.01798800
H	2.28534000	3.28367900	5.81779400
H	1.99471300	2.84827800	0.99947100
H	3.19862500	1.64537300	1.46643400
H	3.41539100	2.58492500	-0.00401000
H	10.35350900	6.99490000	7.07082000
H	13.55332700	4.48098000	6.64632200
H	13.61685100	4.17324700	4.92369300
H	8.14969800	7.33933800	8.08220900
H	7.72912000	8.52533100	6.84078500
H	7.96616700	6.84057000	6.39488700
H	12.26271700	1.98325500	5.33590600
H	10.54034000	2.22784700	5.12073300
H	9.39274600	5.48484700	2.05487600
H	10.44891200	5.14789700	0.67425900
H	9.55782600	6.67236900	0.76008600
H	11.16487900	4.69994600	7.74941100
H	10.04548200	3.49403200	7.15880600
H	11.78225500	5.77335000	2.63298600
H	13.13003500	7.63568200	1.58526500
H	12.68983100	6.35362700	0.45099900
H	11.82605200	7.88472300	0.42651000
H	10.18485600	8.70305900	8.80369400
H	11.35958500	9.25765400	7.60693400
H	9.73397600	9.93371000	7.62980200
H	14.76881000	6.60923300	4.67538800
H	13.50449500	7.65816900	4.03397100
H	13.68826400	6.00367400	3.42416800

H	14.06568600	6.95000700	6.95901900
H	12.41429100	6.65392300	7.49952300
H	12.77548500	8.03722300	6.45225400
H	12.68596800	3.29563600	3.19256600
H	11.70259500	1.86170000	2.94147400
H	10.93840500	3.44642200	2.98538500
H	11.50073300	2.63646800	9.00759600
H	12.93515500	2.87443800	8.01669100
H	11.71893300	1.68766300	7.54279900
C	4.24468000	7.57668000	5.92004400
C	4.80003300	8.76767100	6.38301100
C	5.41227100	9.65846600	5.51584800
C	5.52306000	9.34914500	4.17085800
C	4.97769400	8.17639000	3.64864200
C	4.28785900	7.33423700	4.53670900
C	10.07796900	8.22912700	5.35619200
C	9.55045800	9.40782100	4.82841300
C	9.65373200	9.70285800	3.47945000
C	10.24589600	8.79407900	2.61694500
C	10.78392900	7.59739900	3.08668900
C	10.73709300	7.35971400	4.47087700
H	9.04059400	10.09743200	5.48775800
H	9.24967200	10.63113100	3.09462500
H	10.27573500	9.00676400	1.55670200
H	4.77195400	8.98743100	7.44174000
H	5.83144500	10.58199400	5.89551900
H	6.05626800	10.02024400	3.51114300

Table S15. Cartesian coordinates for the optimized structure of **7** at ZORA/B3LYP-D3BJ/TZVP

Total electronic energy: -21581.33214 E_h

N	3.64553900	6.19004400	3.95916400
C	5.24439000	7.76618300	2.04757800
C	4.28530000	4.96883000	3.85690000
C	1.95297100	4.62307300	3.50165600
C	6.71470700	7.38905100	1.81404400
C	2.22361100	6.14525200	3.53148700
C	3.38629600	2.64412000	4.29341200
C	4.94647600	6.02320800	7.56272500

C	3.67209800	3.51343000	1.93635600
C	3.31704800	3.89982100	3.39657700
C	3.78488200	6.70171000	6.81967900
C	2.81454400	7.35954800	7.80727700
C	4.80010100	8.84985600	1.05721400
C	1.30566400	6.86156700	4.52295000
C	2.02495400	6.78290400	2.14899000
C	3.32124500	2.90461000	5.79648000
C	2.79530600	2.43629200	1.29805100
Au	8.74909800	4.79218000	4.72773800
N	11.35218700	6.17010400	5.03615500
C	9.78840900	7.80836000	6.95318900
C	10.70636100	5.02502000	5.12858000
C	13.04343900	4.62885800	5.60015300
C	8.30398000	7.49379500	7.18756700
C	12.80577600	6.14405900	5.46137000
C	11.57458300	2.69119200	4.76148000
C	10.10670800	6.09421000	1.37201000
C	11.20073800	3.62347600	7.08955900
C	11.65099700	3.95688900	5.64100100
C	11.26526500	6.72310500	2.16073500
C	12.27196400	7.38276100	1.21013700
C	10.26683300	8.88533200	7.93587300
C	13.71288300	6.78185600	4.41349000
C	12.99081300	6.88532700	6.78657300
C	11.73828900	2.92913700	3.26161700
C	12.00959500	2.53248600	7.78920800
C	5.61251000	4.79469800	4.10993200
C	6.81885600	4.67492800	4.35650700
H	4.65232300	6.87334300	1.86491500
H	1.45241700	4.33396400	4.42563700
H	1.28542000	4.36244100	2.68092900
H	7.02451000	6.59782900	2.49426800
H	6.85523400	7.03822500	0.78814800
H	7.37182500	8.24685500	1.97147400
H	4.32373900	2.12713200	4.06939500
H	2.57719300	1.96740200	4.00427600
H	4.56329000	5.26129900	8.24652500
H	5.51059900	6.75100500	8.15049600

H	5.63213700	5.54687000	6.86208700
H	4.71502400	3.18655000	1.93264100
H	3.63829300	4.41605500	1.32142500
H	3.24999700	5.91845200	6.28864600
H	2.38803400	6.60655400	8.47450800
H	3.31975800	8.09939200	8.43211300
H	1.99499200	7.86331600	7.29240000
H	3.76464000	9.15200500	1.22086700
H	5.42318800	9.74323100	1.14133200
H	4.88929000	8.48352000	0.03159100
H	1.57893000	7.91334000	4.62209500
H	1.34391300	6.39955500	5.50710400
H	0.27550600	6.81212200	4.16461400
H	2.63570900	6.29848100	1.38929200
H	2.27935300	7.84234100	2.17321700
H	0.97789500	6.69416500	1.85075400
H	3.33523500	1.96035700	6.34547800
H	4.17395700	3.49746600	6.12494700
H	2.40948200	3.43324600	6.08530300
H	1.73690900	2.70815200	1.31224700
H	2.89801900	1.47543100	1.80422100
H	3.07916300	2.28772700	0.25370600
H	10.34268700	6.89480200	7.15379400
H	13.63219500	4.40870400	6.48934300
H	13.61158300	4.26943100	4.74278000
H	8.14834600	7.15768400	8.21552200
H	7.68045500	8.37253300	7.01790700
H	7.95727200	6.70894500	6.51604900
H	12.33938100	1.99028200	5.10573800
H	10.60583500	2.21721400	4.93995800
H	9.39986100	5.60297800	2.04147700
H	10.49016600	5.35204000	0.66746800
H	9.56211200	6.85144000	0.80487200
H	11.23884800	4.53934200	7.68561700
H	10.14830800	3.33444300	7.05086100
H	11.77175900	5.91002100	2.67461200
H	13.09153500	7.85722700	1.75140100
H	12.69507400	6.63566900	0.53461400
H	11.79513300	8.14656000	0.59282000

H	10.16029300	8.53006100	8.96338700
H	11.31151000	9.15445500	7.77584700
H	9.67231100	9.79629600	7.84177100
H	14.74238100	6.75232100	4.77464800
H	13.44548100	7.82450000	4.23908800
H	13.67353400	6.24708400	3.46738500
H	14.03998600	6.82752600	7.08145900
H	12.39097700	6.45067300	7.58398900
H	12.72797100	7.93737500	6.68525200
H	12.68643400	3.41656400	3.02129300
H	11.71621600	1.98045200	2.72199500
H	10.92989500	3.55005900	2.87502200
H	11.66927500	2.41898600	8.82037800
H	13.07640200	2.76626100	7.82093400
H	11.89423600	1.56539600	7.29866800
C	4.32587700	7.66913700	5.78277100
C	4.93176200	8.85140400	6.20407500
C	5.56366900	9.69244300	5.30124500
C	5.63170300	9.33831900	3.96413300
C	5.03054300	8.17023900	3.49463400
C	4.32251900	7.36665600	4.40704600
C	10.02121100	8.19574400	5.50263200
C	9.44306700	9.37307000	5.02811700
C	9.51709300	9.72378500	3.69118000
C	10.13972500	8.87522700	2.79019900
C	10.72784200	7.68319800	3.20879100
C	10.70427300	7.38260700	4.58233200
H	8.90177300	10.00840500	5.71623700
H	9.05945600	10.64242800	3.34489500
H	10.14556400	9.12645000	1.73766300
H	4.92964200	9.10354700	7.25703000
H	6.02910200	10.60862900	5.64561700
H	6.17832200	9.96702200	3.27300300

Table S16. Cartesian coordinates for the optimized structure of **6** at ZORA/M06/TZVP

Total electronic energy: -21581.93100 E_h

N	3.61259000	6.13620900	4.00938400
C	5.17717600	7.88051800	2.17820900
C	4.25226400	5.01462000	3.76511700

C	1.96675500	4.60302000	3.26257800
C	6.67382800	7.67298600	1.92804800
C	2.14296100	6.09421500	3.60413900
C	3.48996200	2.63436900	3.91642700
C	5.00608900	5.88560900	7.65747800
C	3.82144500	3.79449900	1.67448800
C	3.36748900	3.96412900	3.14717500
C	3.79990100	6.48399700	6.92706500
C	2.79412500	7.05084300	7.92582900
C	4.64299100	8.97950200	1.26092300
C	1.26738200	6.54606800	4.75957400
C	1.93256800	7.01365300	2.41033000
C	3.34676800	2.76046500	5.42358600
C	3.05731200	2.72993500	0.90859500
Au	8.71551600	4.83759500	4.78371800
N	11.36979900	6.13467800	5.00660600
C	9.86981300	7.95576100	6.81913300
C	10.70270500	5.03809000	5.23013900
C	13.00847500	4.61332200	5.77475100
C	8.37416200	7.77697500	7.08904400
C	12.85157500	6.09771900	5.41584500
C	11.47097400	2.66685600	5.10046300
C	10.05931400	5.88483900	1.34240000
C	11.11256700	3.84357200	7.32418700
C	11.59289700	4.00083000	5.85956000
C	11.25189300	6.49186800	2.08426600
C	12.26669600	7.05827800	1.09425900
C	10.42702300	9.06502100	7.70980800
C	13.73449400	6.53383300	4.26028900
C	13.06565900	7.03062600	6.59720000
C	11.64490400	2.78735400	3.59585600
C	11.85304800	2.78091200	8.11600400
C	5.59729800	4.85659700	4.04071900
C	6.79071200	4.74989900	4.31990000
H	4.66954800	6.94559100	1.92473800
H	1.39159200	4.11313300	4.04924400
H	1.39966200	4.48663300	2.33876300
H	7.10774900	6.94342800	2.61458200
H	6.84657900	7.32963000	0.90668600

H	7.21929200	8.60987200	2.05782600
H	4.45869300	2.18687500	3.67410200
H	2.73011000	1.95501700	3.52186300
H	4.68869600	5.09489600	8.33908800
H	5.51699700	6.64818800	8.24945400
H	5.73247200	5.46605200	6.95902900
H	4.89133700	3.56446200	1.67201400
H	3.71973800	4.76198800	1.16899200
H	3.31652600	5.66918400	6.38141900
H	2.42388200	6.26415300	8.58420000
H	3.25176300	7.81139300	8.56074300
H	1.93672500	7.50939300	7.43255000
H	3.58037100	9.16930300	1.40843000
H	5.16854700	9.92069900	1.43178300
H	4.79437700	8.71166700	0.21444800
H	1.51518100	7.56164100	5.07256300
H	1.35029000	5.88211200	5.61908200
H	0.22650000	6.54422200	4.43574400
H	2.52217700	6.70914600	1.54567800
H	2.18601200	8.04414500	2.66152600
H	0.88081500	6.99152900	2.12485000
H	3.37109100	1.78239200	5.90137600
H	4.15805100	3.35121300	5.85552700
H	2.40290800	3.23102700	5.71019700
H	1.97879100	2.89558200	0.94252700
H	3.25117800	1.73143200	1.29917300
H	3.35088000	2.73085300	-0.14012600
H	10.36376700	7.01746000	7.08371000
H	13.55262600	4.50062600	6.71306700
H	13.59888400	4.10893900	5.00841900
H	8.20494200	7.45792900	8.11894700
H	7.83997700	8.71772000	6.94148200
H	7.92226700	7.03820200	6.42158000
H	12.20991000	1.97294900	5.51003400
H	10.48833000	2.23649300	5.32242300
H	9.33507600	5.44995800	2.03686700
H	10.38834400	5.10127400	0.65790900
H	9.53852700	6.64443400	0.75515800
H	11.20894500	4.81467100	7.82463900

H	10.03992100	3.62155100	7.30973400
H	11.73006700	5.68143900	2.63941800
H	13.11253400	7.52933400	1.59539500
H	12.65386300	6.26868900	0.44910900
H	11.81187100	7.80848600	0.44499500
H	10.28380300	8.81878600	8.76279700
H	11.49000900	9.23942800	7.54789200
H	9.91105100	10.00941500	7.52678300
H	14.77294400	6.54301200	4.59243600
H	13.48633000	7.54347100	3.92800700
H	13.66487300	5.85597100	3.41052000
H	14.11723200	7.00750500	6.88383200
H	12.47557500	6.74029400	7.46636700
H	12.81906500	8.05978500	6.33347200
H	12.59840000	3.24946900	3.32736700
H	11.61752300	1.81082900	3.11444100
H	10.84898000	3.39183400	3.15172300
H	11.53827100	2.79050100	9.15868900
H	12.93301300	2.94032700	8.10253100
H	11.66270000	1.78046600	7.72814800
C	4.27739300	7.51990300	5.92606100
C	4.85568800	8.69445200	6.40017900
C	5.43828700	9.60809700	5.54009600
C	5.50452800	9.33562900	4.18620900
C	4.94186800	8.17989300	3.64787700
C	4.27597100	7.31841800	4.53615600
C	10.09656200	8.22331600	5.34182800
C	9.54660400	9.37886600	4.78920900
C	9.61503600	9.63428300	3.43213000
C	10.19602600	8.70695200	2.58563100
C	10.76042100	7.53153300	3.07504500
C	10.74350300	7.33515700	4.46583100
H	9.04445300	10.08305500	5.44404500
H	9.18933400	10.54584200	3.02820900
H	10.19709200	8.88425500	1.51573400
H	4.86708500	8.87998100	7.46852000
H	5.87392500	10.51993900	5.93232900
H	6.01792900	10.02379500	3.52347100

Table S17. Cartesian coordinates for the optimized structure of **7** at ZORA/M06L/TZVP

Total electronic energy: -21582.07691 E_h

N	3.62502700	6.18640700	3.97241400
C	5.29305000	7.72782200	2.07573800
C	4.27462400	4.98268400	3.83769300
C	1.95909700	4.62076300	3.44291500
C	6.78022400	7.44126300	1.85737200
C	2.19887500	6.14004300	3.54463600
C	3.40892000	2.64689700	4.19450200
C	4.91611800	6.03559400	7.57947700
C	3.71149900	3.59710500	1.87217700
C	3.33012900	3.92180100	3.33406400
C	3.75835800	6.69766000	6.82989100
C	2.79224800	7.36539500	7.80311800
C	4.81035300	8.78042500	1.08026000
C	1.28740800	6.79675500	4.57401900
C	2.01389000	6.85427000	2.20735700
C	3.36255000	2.88484600	5.69358900
C	2.88083700	2.51267400	1.21012400
Au	8.73373300	4.78290300	4.73474500
N	11.35120500	6.14976900	5.03074000
C	9.73669700	7.76787100	6.93293800
C	10.69036700	5.00308700	5.14382800
C	13.01976500	4.61107400	5.64577100
C	8.23764400	7.55428600	7.15252200
C	12.80626600	6.11994000	5.45984800
C	11.55553300	2.67039300	4.82923900
C	10.13437800	6.11771600	1.34602000
C	11.16657600	3.64909200	7.13070400
C	11.62639300	3.94828500	5.68377500
C	11.28418400	6.71555700	2.15795100
C	12.30062800	7.38446200	1.23607700
C	10.26014800	8.80389000	7.92615000
C	13.70963700	6.71670500	4.39181500
C	12.97899500	6.91231600	6.74888600
C	11.71235500	2.89975400	3.33577500
C	11.95739900	2.56813900	7.84510400
C	5.60276800	4.82032400	4.09150600
C	6.81199500	4.68731900	4.34478400

H	4.74842100	6.79798800	1.89468200
H	1.43956300	4.28153700	4.34189400
H	1.30964600	4.38418600	2.59766600
H	7.16371700	6.72409500	2.58540600
H	6.94880800	7.03715900	0.85678600
H	7.37015600	8.35756000	1.94722600
H	4.34068400	2.12865300	3.94431700
H	2.59563000	1.97727200	3.89570100
H	4.54205500	5.28291300	8.27701000
H	5.47619700	6.77528300	8.15729100
H	5.61154100	5.55138300	6.89013600
H	4.76927800	3.31602500	1.86449000
H	3.64910800	4.52013100	1.28546600
H	3.22480800	5.90537800	6.30073200
H	2.36439100	6.63104900	8.48808600
H	3.29716400	8.11677700	8.41433200
H	1.97070400	7.86367200	7.28626100
H	3.75588700	9.02521200	1.21101000
H	5.37614800	9.70891100	1.18811200
H	4.94974500	8.43478800	0.05419800
H	1.57582300	7.83525700	4.75158300
H	1.30420000	6.26761500	5.52628200
H	0.25881600	6.79647900	4.20953500
H	2.63381900	6.42116200	1.42239400
H	2.27017200	7.91110500	2.29941900
H	0.97162600	6.79231000	1.88810800
H	3.33995600	1.94160600	6.23990900
H	4.23817800	3.44274900	6.02776300
H	2.47595500	3.45056900	5.99383900
H	1.81151200	2.73405900	1.25103600
H	3.03127100	1.54140900	1.68274200
H	3.14671100	2.40480400	0.15820900
H	10.23495700	6.81367500	7.12313700
H	13.59498300	4.40990400	6.55131000
H	13.60539800	4.22036300	4.81115700
H	8.05300500	7.13578800	8.14383800
H	7.69580700	8.50073300	7.08540300
H	7.80898400	6.87619000	6.41118800
H	12.32234100	1.97395800	5.18345700

H	10.58900100	2.19150600	5.02015600
H	9.41210800	5.61312800	1.99248600
H	10.51255400	5.39329000	0.62171700
H	9.60201600	6.89583800	0.79384900
H	11.21027300	4.58142500	7.70608100
H	10.10653700	3.37902900	7.09278800
H	11.77613700	5.88716900	2.67229900
H	13.11696000	7.84924600	1.79055300
H	12.73201000	6.65719400	0.54607100
H	11.83353300	8.16315500	0.62951200
H	10.09472900	8.47048600	8.95224800
H	11.32499800	9.00279600	7.80582000
H	9.73604700	9.75497300	7.80785100
H	14.73811800	6.73117300	4.75581300
H	13.42629600	7.74574400	4.16100200
H	13.69170000	6.13633000	3.47001700
H	14.02136800	6.86975300	7.06868800
H	12.36161700	6.52239300	7.55813800
H	12.72321900	7.96168000	6.59453700
H	12.64303800	3.42116100	3.09428600
H	11.72373000	1.95619600	2.79036700
H	10.88670200	3.49865400	2.94536000
H	11.63834600	2.47778100	8.88343500
H	13.02900400	2.78098000	7.85380200
H	11.82144600	1.59196700	7.37880000
C	4.30655000	7.65935900	5.79546700
C	4.92733200	8.83146900	6.21861900
C	5.57863900	9.66022500	5.32160400
C	5.66309500	9.29764000	3.98978700
C	5.05121800	8.13894500	3.51408300
C	4.31176700	7.35984300	4.42109500
C	9.99813500	8.16248300	5.49167900
C	9.42719900	9.34162200	5.01566900
C	9.52184300	9.70334800	3.68472500
C	10.15094400	8.85889900	2.78751200
C	10.73750000	7.66697500	3.20505500
C	10.70179200	7.36114400	4.57628000
H	8.87791300	9.97336600	5.70590800
H	9.07245000	10.62762300	3.33860000

H	10.16672200	9.11277000	1.73288500
H	4.92418700	9.08134000	7.27470900
H	6.05343400	10.57174200	5.66909500
H	6.23174000	9.91389500	3.30052900
